# The Glutathione Peroxidase Gene Family in *Gossypium hirsutum*: Genome-Wide Identification, Classification, Gene Expression and Functional Analysis

**DOI:** 10.1038/srep44743

**Published:** 2017-03-16

**Authors:** Mingyang Chen, Kun Li, Haipeng Li, Chun-Peng Song, Yuchen Miao

**Affiliations:** 1 Institute of Plant Stress Biology, State Key Laboratory of Cotton Biology, Department of Biology, Henan University, 85 Minglun Street, Kaifeng 475001, China

## Abstract

The plant glutathione peroxidase (GPX) family consists of multiple isoenzymes with distinct subcellular locations, tissue-specific expression patterns and environmental stress responses. In this study, 13 putative *GPXs* from the genome of *Gossypium hirsutum (GhGPXs*) were identified and a conserved pattern among plant *GPXs* were exhibited, besides this they also responded to multiple environmental stresses and we predicted that they had hormone responsive *cis*-elements in their promoter regions. Most of the GhGPXs on expression in yeast can scavenge H_2_O_2_. Our results showed that different members of the *GhGPX* gene family were co-ordinately regulated under specific environmental stress conditions, and suggested the importance of GhGPXs in hormone treatments and abiotic stress responses.

The generation of reactive oxygen species (ROS) is particularly enhanced when animals and plants are subjected to abiotic and/or biotic stresses. ROS are chemical species that are produced by incomplete reduction of oxygen; they include the superoxide anion (O_2_^−^), hydrogen peroxide (H_2_O_2_), singlet oxygen (^1^O_2_) and the hydroxyl radical (HO**·**). ROS participate in a wide range of important signaling processes including growth, development, the cell cycle, acclimation to stress and programmed cell death^1^,^2^,^3^,^4^. A set of H_2_O_2_-decomposing enzymes like, catalases (CATs) and peroxidases cope with cells to uncontrolled oxidation status^1^,^5^,^6^. Peroxidases may be heme or non-heme peroxidises, the heme peroxidases having a cofactor in their active site (such as ascorbate peroxidases, APXs) or, a redox active cysteine (Cys) or selenocysteine (Sec) residues respectively^7^. Thiol peroxidases such as thioredoxin (Trx) peroxidases or peroxiredoxins (Prxs) and glutathione peroxidases (GPXs) are belongs to family of non-heme peroxidases. Moreover, GPXs, glutathione S-transferases (GSTs) with GPX activity and Prxs are also able to decompose alkyl hydroperoxides in addition to H_2_O_2_^8^,^9^. For decades, GPXs by using glutathione (GSH) or other reducing equivalents as a reductant, have been known to catalyse the reduction of H_2_O_2_ or other organic hydroperoxides in to water or the corresponding alcohols^10^.

The GPXs were the first solenoenzyme that was discovered from mammals^11^,^12^. The reaction takes place at a single redox centre with Sec as the redox-active residue in selenocysteine-GPXs (Sec-GPXs). The catalytic centre of Sec-GPXs was first characterised as a triad composed of Sec or Cys, glutamine (Gln) and tryptophan (Trp)^13^, but later turned out to be a tetrad with an additional asparagine (Asn)^14^,^15^. In contrast, the peroxidative Sec was replaced by a Cys and function via a second redox centre that contains a resolving Cys in GPXs of most non-vertebrates. The former type of enzyme is more or less specific for GSH, while the latter is reduced by “redoxins”. The common denominator of the GPX family is the first redox centre comprising (seleno) Cys, Trp, Asn and Gln^15^,^16^; this kind of GPXs are shown to be reduced by redoxins, namely, thioredoxins or related proteins with a CXXC motif in plants, yeast, insects and protists^15^,^17^.

GPX genes from a range of plant species, such as *Nicotiana sylvestris*^18^, *Citrus sinensis*^19^,^20^, *Avena fatua*^21^, *Spinacia oleracea*^22^, *Brassica campestris*^23^, *Helianthus annuus*^24^, *Pisum sativum*^25^, *Lycopersicon esculentum*^26^, *Arabidopsis thaliana*^27^,^28^,^29^, *Oryza sativa*^30^, *Triticum aestivum*^31^, *Panax ginseng*^32^, *Populus trichocarpa*^33^,^34^ and *Thellungiella salsuginea*^35^, have been isolated and characterised. Unfortunately very few reports on genome-wide identification and characterisation of GPX family proteins to date have been reported. Furthermore it has been reported that the plant GPX family not only consists of multiple GPXs with distinct subcellular localisations and functions but also exhibited differential tissue-specific expression patterns, responses to environmental stress, functioning in co-ordination against ROS, immune responses, regulating the growth and development of plant root, and stomatal movement^27^,^28^,^36^,^37^ based on studies of four GPX family proteins from *A. thaliana*^36^,^37^, *Lotus japonicus*^38^, *P. trichocarpa*^5^ and *T. salsuginea*^35^. Therefore, these results also highlighting the importance of GPX study at the genome-wide level.

Cotton has a long cultivation history and is currently the main source of natural textile fibre and an important crop for oil and biofuel^39^. There are four commercially grown cotton species, *Gossypium hirsutum, Gossypium barbadense, Gossypium herbaceum* and *Gossypium arboretum*^40^. *G. hirsutum* and *G. barbadense* are the two most commonly cultivated species and producing ~98% of the textile fibre worldwide. Moreover, it requires to mention that there are no reports providing insight on the expression profiling of GPXs in *G. hirsutum* under abiotic stress conditions. In the present study, we have identified 13 GPX genes from *G. hirsutum* and not only their gene structure and promoter sequences were analysed, but also their potential subcellular locations were predicted. We examined the expression of *GhGPX* transcripts from the leaves and roots of *G. hirsutum* under short-term exposure to salt, osmotic and abscisic acid (ABA)-induced stresses to address their role in these stresses. Further exploring their role under abiotic stresses, *gpx3Δ* (H_2_O_2_-sensitive mutant) of *Saccharomyces cerevisiae* were complemented with the GhGPXs, and it suggests that GhGPXs have similar function to GPX3 in yeast, revealing their participation in the oxidative stress response.

## Results

### Identification and characterisation of *GhGPX* genes

We searched the *GhGPX* sequences from the Cotton Genome Project (CGP) database (http://cgp.genomics.org.cn/page/species/index.jsp)^39^, which is the recent release of the first version of the *G. hirsutum* genome by using the coding sequences (CDSs) of *GPX1*–*8* from *A. thaliana (AtGPX*). 12 *GhGPX* genes had been identified and which were represented putative *GhGPX1, GhGPX2, GhGPX3, GhGPX4, GhGPX5, GhGPX6, GhGPX7, GhGPX8, GhGPX11, GhGPX12, GhGPX13* and *CotAD_39521*. Moreover, we cloned CDSs of the 12 *GhGPXs* in at least three independent experiments and it was found that a nucleotide sequence of 45-bp inserted between nucleotides 373 and 374 bp of the *GhGPX2* CDS, leading to a 15-amino-acid (GFLGSRIKWNFTKFL) insertion between 124S (Ser) and 125 V (Val). However, this 45-bp nucleotide sequence was considered as part of an intron on CGP database^39^. Thus it could be possible that *GhGPX2* has two transcripts in young seedlings. We also found a 39-bp nucleotide sequence insertion behind the 441-bp of the CDS of *GhGPX4* (marked with yellow in the Supplementary Data 1), and this 39-bp nucleotide sequence was not exist in the intron of *GhGPX4* from the database. Therefore, we had cloned a part of the DNA sequence of *GhGPX4* and found an error in the sequence of *GhGPX4* that was reported on the database (the sequence between two yellow marked sequences of the cloned *GhGPX4* in Supplementary Data 1). 72% identity with AtGPX6 to the 43-225-amino-acid sequence of the N terminus of CotAD_39521 realized it was a specific GPX, however, its amino-acid sequence (226–545) had 72% identity with a cysteinyl-tRNA synthetase (AT5G38830). Moreover, the cloned gene showed different CDS (Supplementary Data 1) to *CotAD_39521*, when we cloned *CotAD_39521* by using two sets of primers (Supplementary Table S1); Gh_A12G2084 and Gh_D12G2260 were found to have high homology with CotAD_39521, when the protein sequence of CotAD_39521 was used for a Blast search with another genome database of the *G. hirsutum*^41^. This suggested us an error in the gene splicing of *CotAD_39521*. The CDSs of *Gh_A12G2084* and *Gh_D12G2260* were also cloned by PCR and we found that there was a deletion of 13-bp nucleotide sequence behind the 675-bp nucleotide (from the start codon, marked with yellow in Supplementary Data 1) of *Gh_A12G2084* and *Gh_D12G2260*, this frame shift error evoked the emergence of a termination codon (marked with red in Supplementary Data 1). Thus it is possible that both *Gh_A12G2084* and *Gh_D12G2260* have two transcripts, *Gh_A12G2084* and *Gh_D12G2260* represented putative *GhGPX10* and *GhGPX9*, respectively. Therefore, the renewed *GhGPX2, GhGPX4, GhGPX9* and *GhGPX10* sequences were used in the following bioinformatic analysis of the *GhGPX* sequences. The CDSs of 13 *GhGPXs* were shown in the Supplementary data 2, and the genomic locations of 13 *GhGPXs* were shown in Table 1.

Notably, twice of some *GPX* genes were found in allotetraploid than those of diploid cotton. Three genes had one copy in the diploid, while five homologous genes, from the A and D subgenomes (*GhGPX2, 4, GhGPX3, 5, GhGPX7, 11, GhGPX8, 12* and *GhGPX9, 10*) were found in the tetraploid *G. hirsutum*. The sequences of homologues genes were inconsistent, which might have been resulted from loss of gene during their individual evolutionary processes or from assembly error in partial chromosomal regions, although this requires confirmation. Although *GhGPX6* and *GhGPX9* were located in chromosome 8 and 12, respectively, the same part sequence of *GhGPX9* (190–702 bp) was shared by CDS of *GhGPX6* (513 bp) and the promoter sequence of *GhGPX6* was also similar to that of *GhGPX9*. This data vividly suggested that *GhGPX6* is another copy of *GhGPX9* and they may have two different transcripts.

To confirm the potential functions of the 13 putative GhGPXs, we analysed the homology between GhGPX and AtGPX protein sequences using WU-BLAST2 (http://www.arabidopsis.org/wublast/index2.jsp). Using the protein sequences of GhGPXs, we found the highest homology of AtGPX to each GhGPX protein (Table 2), for example, the amino acid sequences of GhGPX1 and AtGPX1 had 79% identity, while GhGPX6 and AtGPX6 had 78% identity. This suggested that GhGPXs may have similar functions to those of their homologues in *Arabidopsis*.

### Identification of intron-exon from *GhGPX* family genes

**Figure 1:**
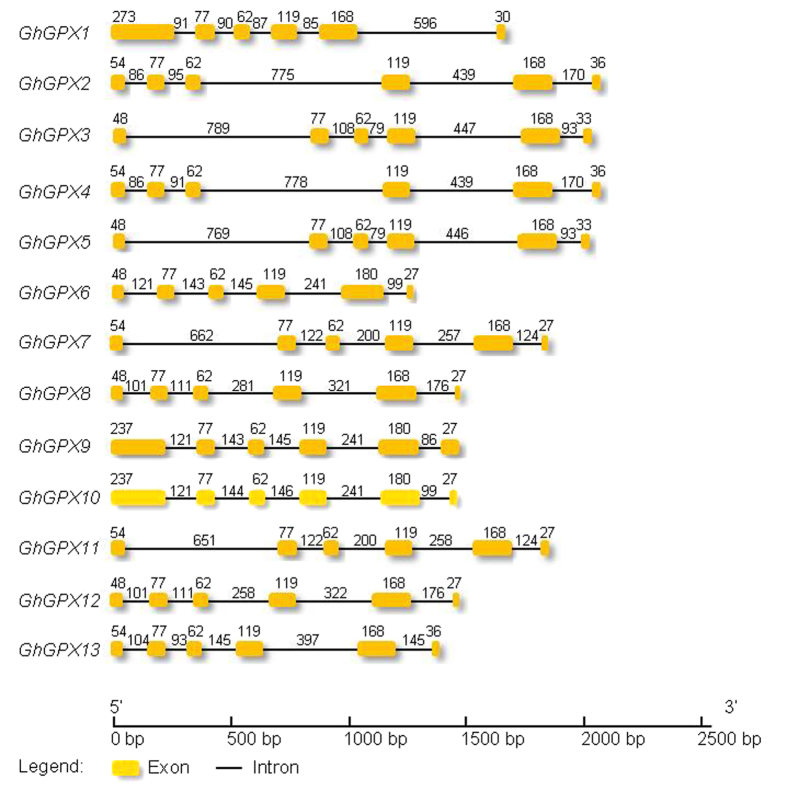
Genomic organisation of the *GhGPX* gene family. The exons are depicted in yellow boxes and introns by black lines. All exon and intron lengths are drawn to scale.

Open reading frames (ORFs) of all 13 *GhGPX* genes consisted of six exons and five introns (Fig. 1, and the sequences were shown in Supplementary data 3). Similar exon-intron organisations in GPX genes were also reported from various plants like eight *AtGPXs*^36^, the six GPX genes from *P. trichocarpa (PtGPX*)^5^, the five GPX genes from *L. japonicus (LjGPX*)^38^ and the eight GPX genes from *T. salsuginea (TsGPX*)^35^, as well as some GPX genes from citrus^20^ and wheat^31^, suggesting a high degree of conservation in the exon-intron organisation among plant species. The second, third and fourth exons showed same length were of 77, 62 and 119-bp respectively, showed a same length in all 13 *GhGPXs*. The length of the fifth exon of 13 *GhGPXs* was also similar, with a length of 180 bp in *GhGPX6, GhGPX9* and *GhGPX10*, and other *GhGPXs* were 168 bp.

### Analysis of putative *cis*-acting regulatory elements from *GhGPXs* promoters

The plant *GPX* genes are well known to be involved in responses to various environmental stresses^29^,^31^,^32^,^36^. Therefore, we analysed the promoter regions of all 13 *GhGPX* genes to find their putative *cis*-acting regulatory elements involved in stress and hormone signaling (Table 3) by using PlantCARE software^42^. Multiple different *cis*-acting regulatory elements were found in each *GhGPX* promoter region and their number varied with the individual promoter (Table 3 and Supplementary data 4). Almost all *GhGPX* promoters contained *cis*-acting elements that were responsive to hypoxia (*GhGPX1*–*3, 5*–*11*), methyl jasmonate (MeJA) (*GhGPX1*–*5, 7, 8, 11, 12*) and drought (*GhGPX1*–*3, 5*–*12*); many promoters of *GhGPX* contained elements that were responsive to salicylic acid (SA) (*GhGPX1, 3, 5, 7, 8, 10*–*13*), gibberellin (*GhGPX1, 2, 5, 6, 9*–*11, 13*) and auxin (*GhGPX1, 4*–6, *8*–*10*); whereas fewer *GhGPX* promoters contained elements that were responsive to ABA (*GhGPX6, 7, 9, 10, 11*), ethylene (*GhGPX3, 8, 12, 13*) and low temperature (*GhGPX2, 4, 8, 13*). These findings indicate that *GhGPX* genes may also be involved in responses to various environmental stresses and plant hormones.

### Predicted properties of GhGPX proteins

**Figure 2:**
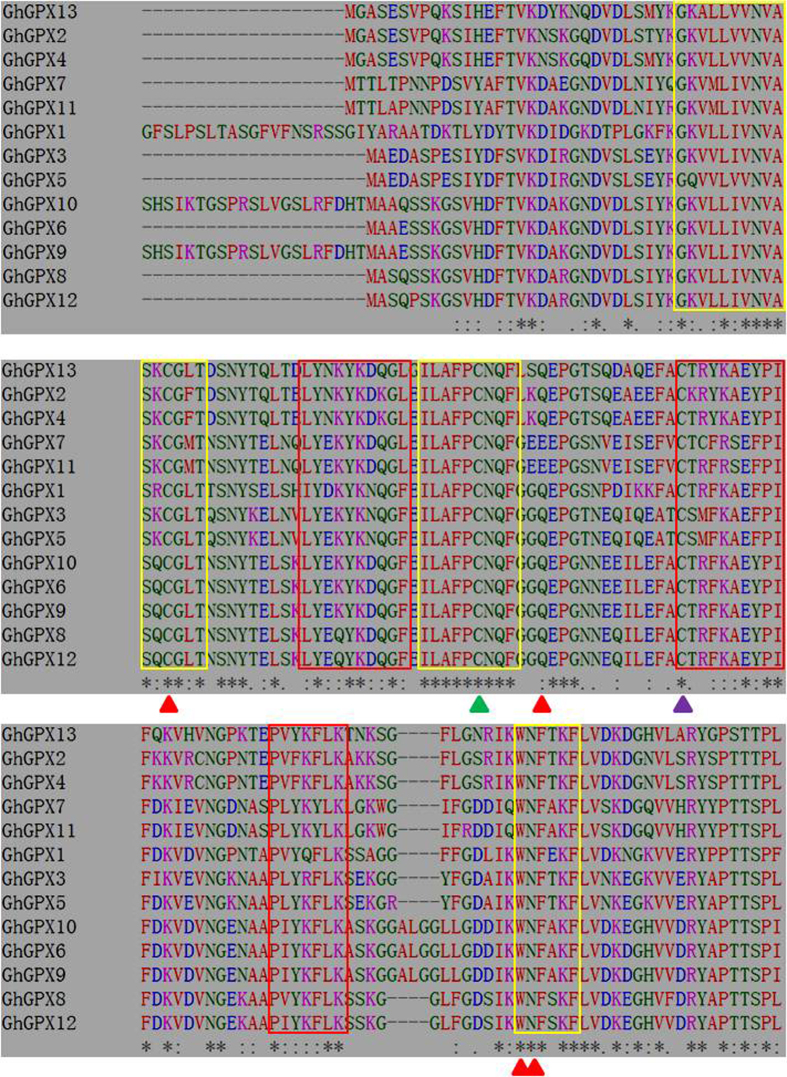
Amino acid sequences of GHGPX proteins. Multiple GhGPX sequences alignment using Clustal Omega (http://www.ebi.ac.uk/Tools/msa/clustalo/). The three conserved domains found in most animal and plant GPXs are marked in yellow boxes; the amino acid residues that form part of the proposed catalytic site of the GPXs are marked with red triangles, including the strictly conserved Cys (C), Gln (Q), Asn (N) and Trp (W); other conserved domains are marked with red boxes. The cysteine residue marked with a red triangle is replaced by Sec in mammalian phospholipid hydroperoxide GPXs; the other two conserved Cys residues are marked with green and purple triangles. Amino acid residues that are identical (or have similar properties) in all 13 sequences are marked with a black asterisk below the sequences. Amino acid residues that have similar properties are marked with tandem points below the sequences. Amino acid residues that are identical at least in five sequences are marked with a point below the sequences.

To analyse the properties of all 13 GhGPX proteins, the amino acid sequences of GhGPXs were subjected to alignment. The alignment result revealed that all the protein sequences contained three conserved Cys residues (Fig. 2, marked with triangles) present in most of the plant GPXs, as well as three conserved domains: GPX signature 1, GPX signature 2 and a domain WNF(S/T)KF (Fig. 2, marked with yellow boxes) found in GPXs of most plant and mammalian^43^, besides this the other conserved sequences were also presented, such as (L/I)Y(E/D/N)(K/Q)YK(D/N)(Q/K)G(F/L), C(T/S/K)(R/C/M)(F/Y)(K/R)(A/S)E(Y/F) PIF and P(V/L/I)(Y/F)(K/Q)(Y/F)LK (Fig. 2, marked with red boxes). The comparing between the amino acid sequences of GhGPXs and previously reported GPXs of other plant species revealed that redox centre embraced with a Cys residue (known to be a catalytic site) as well as Trp, Asn and Gln conserved residues^15^ (Fig. 2, marked with red triangles).

**Figure 3:**
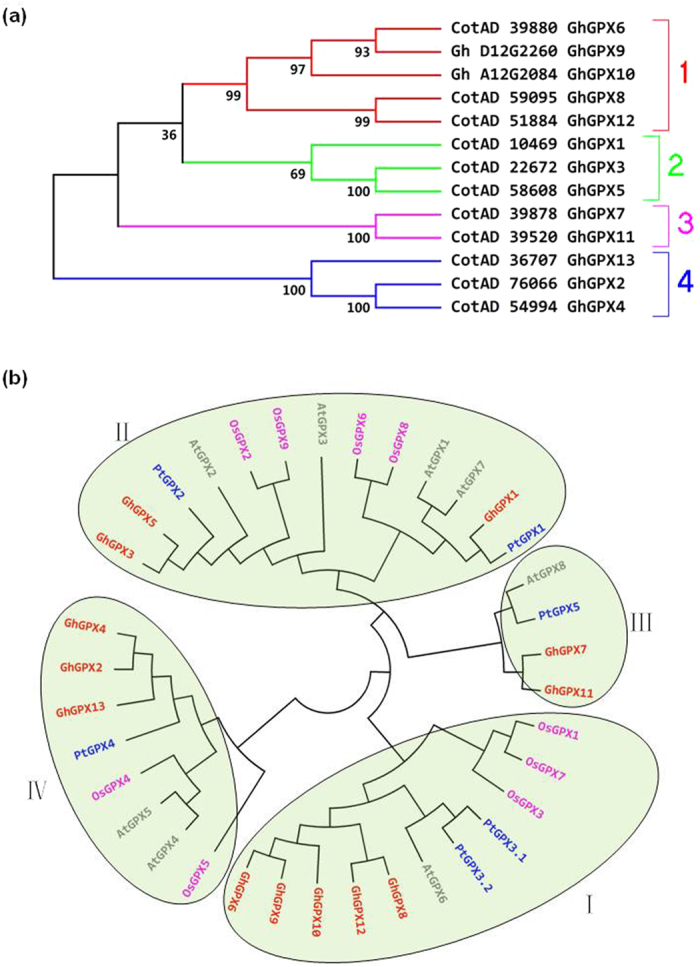
Phylogenetic analysis of GPX proteins in plants. Only complete sequences were considered. (**a**) Phylogenetic analysis of GhGPXs. (**b**) Phylogenetic analysis of GPX in *G. hirsutum, A. thaliana, O. sativa* and *P. trichocarpa*. The tree was constructed using the neighbour-joining method of ClustalW, with 1000 bootstraps, and the bar indicates 0.1 substitutions per site. Different species of GPX proteins are denoted by different colours: red, *G. hirsutum*; blue, *P. trichocarpa*; pink, *O. sativa*; grey, *A. thaliana*. Abbreviations of plant species: Gh, *G. hirsutum*; At, *A. thaliana*; Os, *O. sativa*; Pt, *P. trichocarpa*. Accession numbers for *G. hirsutum* genes are indicated in Table 1 and those of other plant species are as follows (in brackets): AtGPX1 (At2g25080); AtGPX2 (At2g31570); AtGPX3 (At2g43350); AtGPX4 (At2g48150); AtGPX5 (At3g63080); AtGPX6 (At4g11600); AtGPX7 (A4g31870); AtGPX8 (At1g63460); OsGPX1 (Os04g0556300); OsGPX2 (Os06g0185900); OsGPX3 (Os02g0664000); OsGPX4 (Os03g0358100); OsGPX5 (Os11g0284900); OsGPX6 (P0568D10.7); OsGPX7 (Os04g0556300); OsGPX8 (Os06g0185900); OsGPX9 (LOC_Os11g18170); PtGPX1 (ABK96776); PtGPX2 (DT518382); PtGPX3.1 (ABK96047); PtGPX3.2 (ABK94488); PtGPX4 (ABK95195); PtGPX5 (2P5Q_A).

The 13 GhGPX proteins can be divided into four major groups: GhGPX6, 8–10, 12 were classified into group 1; GhGPX1, 3, 5 into group 2; GhGPX7, 11 into group 3 and GhGPX2, 4, 13 into group 4 (Fig. 3a). Moreover, based on the phylogenetic tree analysis of GPXs from *A. thaliana, G. hirsutum, O. sativa* and *P. trichocarpa*, the GPXs could also be divided into four clades. Specifically, the GPXs of *A. thaliana, G. hirsutum, O. sativa* and *P. trichocarpa* were divided into each clade, which indicated that each GPX has a homologue in the other three kinds of plant, and thus they might have similar functions in different kinds of plant. These data suggested that the conserved nature of evolution of GPX family protein among the four plant species, it is consistent with the same number of exons and introns found in GPXs of different plants species (Fig. 1). GhGPX6, 8–10, 12 were grouped into clade I, GhGPX1, 3, 5 into clade II and the GPXs in sub-classification of clade II, including GhGPX1, PtGPX1, AtGPX1, 7 and OsGPX6, 8 were all predicted to have the subcellular localisation of the chloroplast or mitochondria (Table 4). Clade III composed of GhGPX7, 11, while GhGPX2, 4, 13 were in clade IV (Fig. 3b). These results suggested that GhGPXs have similar characteristics like AtGPXs, OsGPXs and PtGPXs.

The GhGPX proteins can be divided into two categories based on the difference between the length of amino acid sequences: the first category comprised GhGPX1 (242 amino acids, 26.65 kDa), GhGPX9 (233 amino acids, 25.49 kDa), and GhGPX10 (233 amino acids, 25.56 kDa); while the second category comprised with the other GhGPXs having 166–171 amino acids (Table 4). Protein subcellular localisation prediction programmes suggested most of the GhGPX proteins localised in the cytoplasm, while GhGPX1 in chloroplasts and GhGPX9, 10 in chloroplasts or mitochondria (Table 4). The putative homologues of GhGPXs in *Arabidopsis* and poplar are also shown in Table 4.

Although GhGPX9 and GhGPX10 (consisted of ~233 amino acids) were clustered in clade I along with GhGPX6, 8, 12, which each had about 166–170 amino acids. The GhGPX1 comprised of 242 amino acids and a chloroplast transit peptide signal (1–30 amino acids) was at the N-terminal; in contrast, the 1–30 amino acids residues of GhGPX9, 10 may be a mitochondrial or chloroplastic targeting peptide, as predicted by iPSORT and TargetP, which were used to predict the subcellular localisation of eukaryotic proteins (http://ipsort.hgc.jp/index.html#predict; http://www.cbs.dtu.dk/services/TargetP/).

### Differential expression pattern and subcellular localisation of *GhGPX* products

To determine which tissues normally express *GhGPX* genes, gene-specific primers were used for transcription analysis in roots, stems, flowers, cotyledons and leaves of *G. hirsutum*. We had designed six specific primers for real-time quantitative PCR (qRT-PCR): *GhGPX1*–*3, 5, 8, 13*, owing to the high homology between *GhGPX* genes.

**Figure 4:**
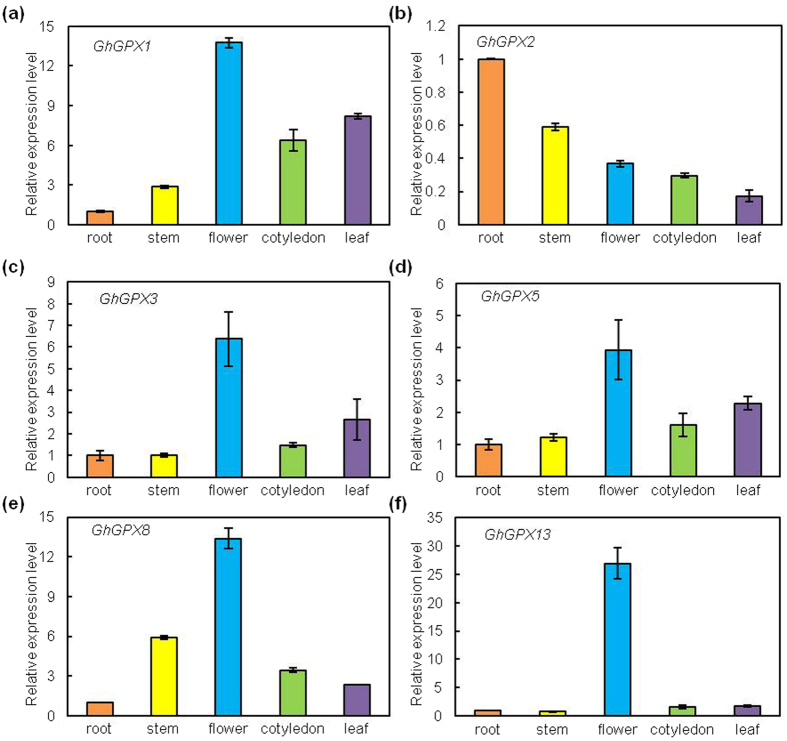
Expression of *GhGPX* genes in leaves, flowers, roots, stems and cotyledons. Relative expression of each gene in organs with respect to leaves, flowers, roots, stems and cotyledons. (**a**) *GhGPX1*, (**b**) *GhGPX2*, (**c**) *GhGPX3*, (**d**) *GhGPX5*, (**e**) *GhGPX8*, (**f**) *GhGPX13*. Cotton seedlings were grown in sterile culture pots under long-day conditions (16-h photoperiod) with 26/20 °C day/night temperatures for 25 days. The leaves were the first true leaves. Steady-state mRNA levels were normalised with respect to *Ubiquitin7* (UBQ7, DQ116441) and are expressed relative to the values found in the roots, which were given an arbitrary value of 1. All data are means ± SD of six replicates.

As shown in Fig. 4a, the *GhGPX1* gene encoding a putative chloroplast protein not only showed expression pattern in roots, stems, flowers, leaves and cotyledons, but was also with most strongly expression level in flowers, and high transcript level in leaves. However, it was nearly absent in roots; the possible explanation to this may be due to the predicted subcellular localisation of *GhGPX1* being the chloroplast. The *Arabidopsis* chloroplastic GPXs (AtGPX1 and AtGPX7) play a role in scavenging of active oxygen species under photo-oxidative stress^28^, and AtGPX7 also shows the highest expression level in flowers (http://bar.utoronto.ca/). In addition, the evolutionary relationship among *AtGPX1, AtGPX7* and *GhGPX1* is close (Fig. 3b), indicating the role of GhGPX1 in protection against ROS in response to photosynthesis. *GhGPX2* transcript was high in roots, and with the lowest expression level in leaves (Fig. 4b). *GhGPX3* and *GhGPX5* were homologous from the A and D subgenomes, the transcripts of them showed similar patterns, with low levels in stems and high expression level in flowers and leaves (Fig. 4c,d), which is consistent with their close evolutionary relationship (Fig. 3a). *GhGPX8* transcripts were present at high levels in flowers and stems, and the region of the promoter had many *cis*-elements, including SA, gibberellins and ethylene-responsive elements. This suggested that *GhGPX8* may regulate the seedling growth and development (Fig. 4e). The level of *GhGPX13* transcript was high in flowers and low in other tissues; compared with roots, the transcript in flowers was found to be nearly 30 times (Fig. 4f). This data indicates that GhGPXs play roles in specific tissues and in different growth periods.

**Figure 5:**
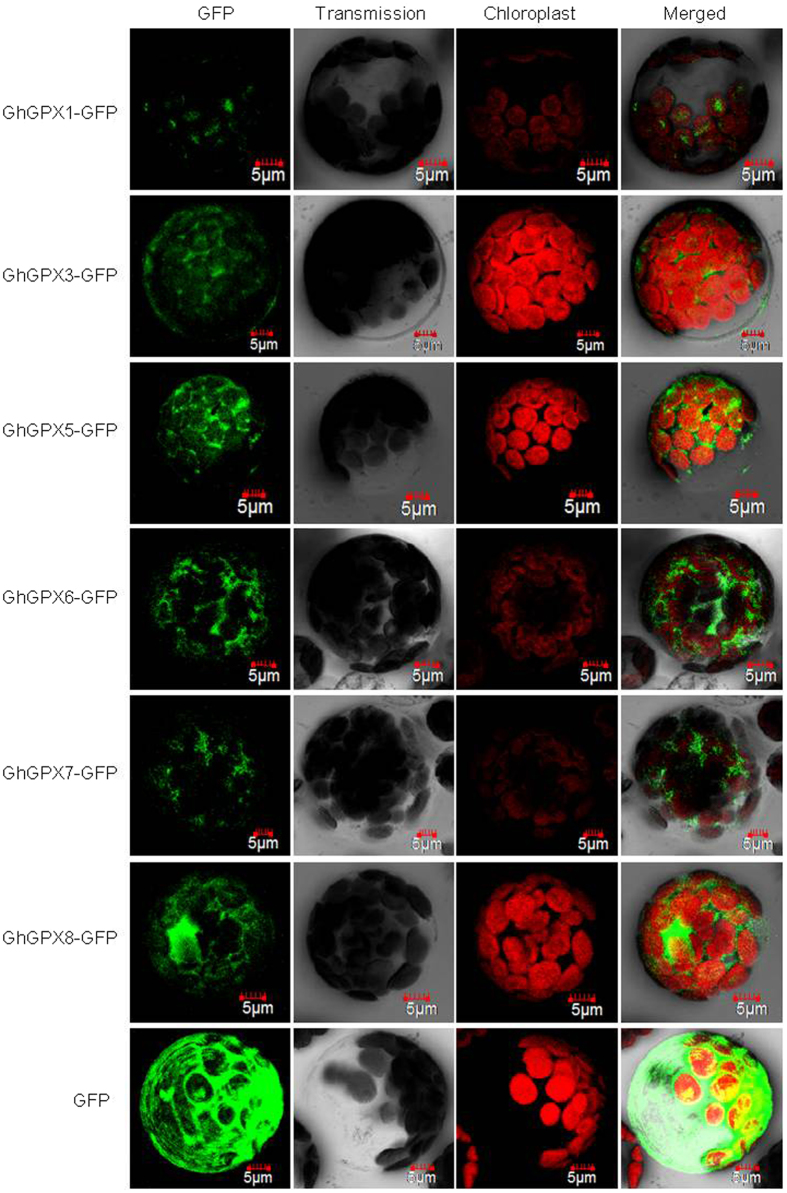
The subcellular localisation of GhGPX proteins in mesophyll protoplast of *Arabidopsis*. Localisation of GFP signals from GhGPX proteins fused with GFP. Epifluorescence, chloroplast autofluorescence, bright field and merged images of *Arabidopsis* mesophyll protoplasts transfected with constructs expressing different fusion proteins. Bars = 5 μm.

To test the subcellular localisations of GhGPXs, N-terminal GFP-fused GhGPX proteins were expressed in the protoplast of *Arabidopsis* leaves. As shown in Fig. 5, GhGPX1 was expressed in chloroplasts, while GhGPX3, 5–8 were located in the cytoplasm. This result is consistent with the prediction of the subcellular localisation of GhGPXs as shown in Table 4.

### Expression analyses of *GhGPX* genes in response to various stress conditions

It is widely accepted that diverse environmental conditions, such as exposure to high concentrations of salt and metals, or high temperatures, induce oxidative stress in plants. Many plant species have been shown to respond to environmental stress by increasing the expression of *GPXs*, which might protect the cells from damage^19^,^27^,^28^,^29^,^35^,^44^. However, there were no studies of whether *GhGPXs* could response to the multiple environmental stresses. Considering that multiple stress-response *cis*-acting elements were predicted in the promoters of *GhGPX* genes (Table 2), we used qRT-PCR to investigate the expression profiles of *GhGPXs* when the plants were under high-temperature, high-salinity, osmotic and heavy-metal stress.

**Figure 6:**
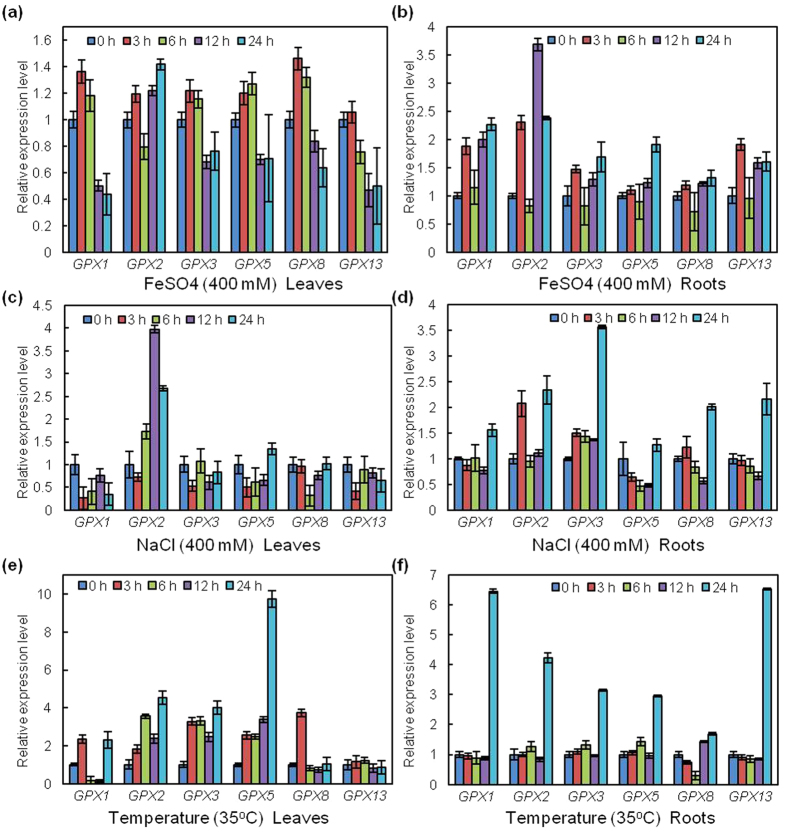
Effect of various stresses on the expression of *GhGPX* genes in leaves and roots. *GhGPX* gene transcripts in response to various stress conditions were analysed by qRT-PCR. Cotton seedlings were grown in sterile culture pots under long-day conditions (16-h photoperiod) with 26/20 °C day/night temperatures for 25 days and then treated with 400 mM FeSO_4_ (**a**,**b**), 400 mM NaCl (**c**,**d**) and a high temperature (35 °C) (**e**,**f**). mRNA levels were normalised with respect to *GhUBQ7* and are expressed relative to the values at 0 h (control), which were given an arbitrary value of 1 for each gene. Data represent the means ± SD of at least three replicates.

Short-term exposure of *G. hirsutum* plants to heavy metal stress (400 mM ferrous sulphate) induced or suppressed the expression of some members of the *GhGPX* genes at different processing times and the effect varied with the tissue, such as leaves and roots (Fig. 6a,b). Compared with the control samples, the *GhGPX1*–*3, 5, 8, 13* expression levels changed not more than four times. In leaves, the increased of transcript level could be seen within 3–6 h of treatment for *GhGPX1, 3, 5, 8*; however, within 12–24 h of treatment, the expression level decreased. However, the transcript level of *GhGPX2* decreased when the seedlings were treated with ferrous sulphate for 6 h but increased after 12 h of treatment. In addition, the transcript level of *GhGPX13* increased with ferrous sulphate treatment for 3 h, and then decreased after 6 h. In roots, the increased in transcript level could be seen within 3 h of treatment for *GhGPX1*–*3, 5, 8, 13*, with a decline after 6 h of treatment and a further increased after 12 h of treatment (Fig. 6b).

The expression patterns of *GhGPX* genes were also analysed in detail following the treatments of salt (Fig. 6c,d) and high-temperature (Fig. 6e,f). Salt stress with 400 mM NaCl treatment for 24 h increased the transcript levels for *GhGPX1*–*3, 5, 8, 13* in roots. The expression level of *GhGPX2* was induced in leaves by 24 h treatment with 400 mM NaCl, and the transcript levels of *GhGPX1, 3, 5, 8, 13* decreased in leaves after treatment with 400 mM NaCl for 3 h (Fig. 6c). A high temperature (35 °C) induced expression of *GhGPX1*–*3, 5, 13* after treatment for 24 h in leaves and roots (Fig. 6e,f); however, the transcript level of *GhGPX8* increased in roots after 24 h treatment and in leaves at 3 h treatment (Fig. 6f).

### Induced expression patterns of *GPX* genes under multiple phytohormone treatments in *G. hirsutum*

**Figure 7:**
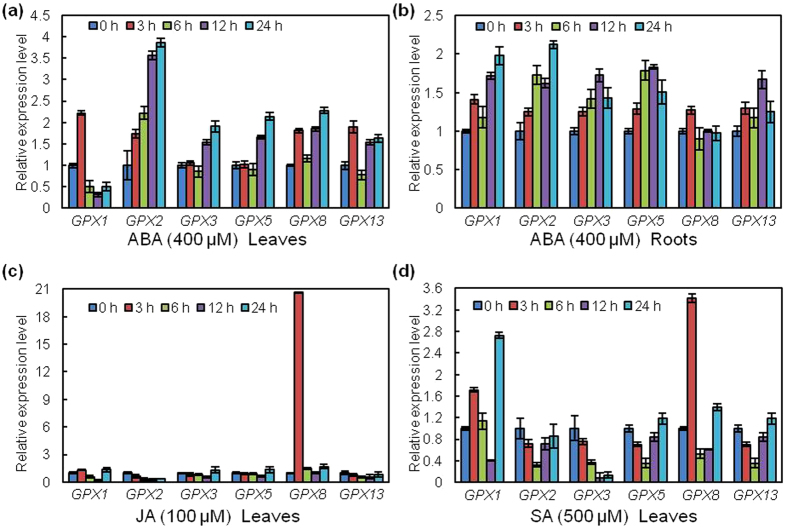
The effects of different plant hormones on the steady-state transcript level of *GhGPX* genes by qRT-PCR. Leaves or roots of *G. hirsutum* exposed to 400 μM ABA (**a**,**b**), 100 μM JA (**c**) and 500 μM SA (**d**). mRNA levels were normalised with respect to *GhUBQ7* and are expressed relative to the values at 0 h (control), which were given an arbitrary value of 1. Data represent the means ± SD of at least three replicates.

Previous results indicated that plant hormones play a direct role in regulation of a rice GPX gene^45^. AtGPX3 might mediate ABA and oxidative signaling through physical interaction with the 2C-type protein phosphatase ABA INSENSITIVE2 (ABI2) and, to a lesser extent, with ABI1^27^. AtGPX1 and AtGPX7 were also shown to regulate ROS and SA pathways, which affect leaf development, light acclimation, basal defence and cell death programmes^28^. RNA was isolated from roots and leaves of 10-day-old *G. hirsutum* plants for further study of the effects of signaling molecules on regulation of the expression level of the *GhGPX* gene family. The leaves were then sprayed with SA, jasmonic acid (JA) and ABA, and the roots were dipped in ABA. We performed qRT-PCR analyses to examine the expression levels of the *GhGPX* genes in response to the three phytohormones. ABA induced the expression of *GhGPX2* in both leaves and roots with respect to controls (Fig. 7a,b). A slight increased in expression of *GhGPX3* and *GhGPX5* were observed in leaves and roots, while *GhGPX3* and *GhGPX5* showed similar expression patterns under ABA treatment. The expression of *GhGPX1, GhGPX8* and *GhGPX13* were also induced by ABA in leaves; however, in roots, ABA induced the expression of *GhGPX1* and *GhGPX13*, while no obvious change was found for *GhGPX8*. We previously reported that the enhanced production of H_2_O_2_ in guard cells on treatment with ABA, resulted stomatal closure^46^. In addition, it was shown that AtGPX3 might play dual and distinctive roles in H_2_O_2_ homeostasis, acting as a general scavenger and specifically relaying the H_2_O_2_ signal as an oxidative signal transducer in ABA and drought stress signaling^27^. Hence, ABA induced expression of *GhGPX* genes may involve enzymatic responses to oxidative stress. JA treatment specifically increased the expression levels of *GhGPX8* among the six analysed *GhGPX* genes (Fig. 7c). SA treatment induced slight up-regulation of the expression level of *GhGPX1* and *GhGPX8*, and down-regulation of *GhGPX3* (Fig. 7d). These results indicated that individual members of the *GhGPX* gene family respond differentially against abiotic stress and plant hormones, and these findings may provide a clue to explain the differential regulation of transcripts under stress conditions.

### Functional complementation of the H_2_O_2_-sensitive yeast growth by transforming GhGPXs in *gpx3Δ* mutant yeasts

**Figure 8:**
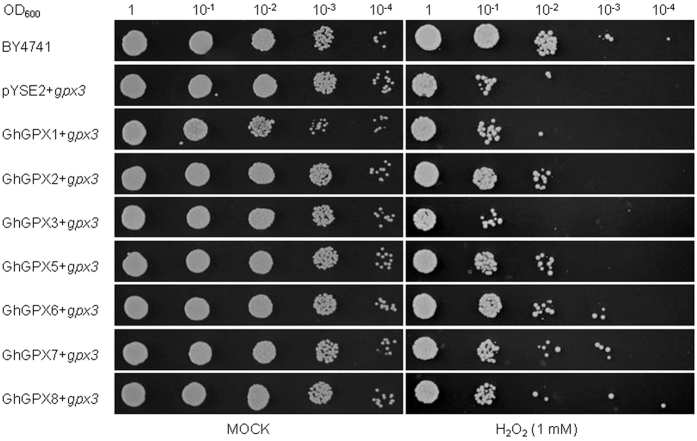
Some GhGPXs can functionally complement the H_2_O_2_-sensitive morphology in *gpx3Δ*. Growth of yeast strains on solid minimal medium containing 1 mM H_2_O_2_ (MOCK represents equal volume of H_2_O). Strain BY4741 (*gpx3Δ*, WT) transformed with plasmid *pYSE2* and *pYSE2*-*GhGPXs* was incubated at 30 °C. Pictures were taken 4 d later.

The GPX homolog *YIR037W*/*HYR1*/*GPX3* was found in the *Saccharomyces* Genome Database, and a previous study indicated that *gpx3Δ* mutant was hypersensitive to peroxides, and with decreased ~57% GPX activity in *gpx3Δ* mutants with respect to wild type (BY4741)^47^. Plant GPXs contain Cys instead of Sec at their active site and some of them have both GPX and thioredoxin peroxidase activities, and the thioredoxin regenerating system is much more efficient *in vitro* than the GSH system. At present, the functions of these enzymes in plants are not addressed. To test whether the GhGPXs can detoxify H_2_O_2_, the CDSs of *GhGPX1, GhGPX2, GhGPX3, GhGPX5, GhGPX6, GhGPX7* and *GhGPX8* were cloned into the *pYES2* vector and the recombinant plasmids were transformed into *gpx3Δ* cells. As shown in Fig. 8, the *gpx3Δ* cells were sensitive to H_2_O_2_ when OD_600_ was <0.01, and few yeasts were seen under 1 mM H_2_O_2_ treatment compared with that of controls, in terms of the number of wild-type BY4741 yeast cells. The *gpx3Δ* cells, which had been transformed with *GhGPX1, GhGPX3, GhGPX5, GhGPX6, GhGPX7* and *GhGPX8*, were insensitive to H_2_O_2_ compared with the *gpx3Δ* cells when OD_600_ was 0.1; however, the *GhGPX2*-transformed *gpx3Δ* was found to be sensitive to H_2_O_2_. This suggested that GhGPX1, GhGPX3, GhGPX5, GhGPX6, GhGPX7 and GhGPX8 can scavenge H_2_O_2_ in yeast, with functions similar to that of GPX3 in yeast. The *gpx3Δ* cells, which had been transformed with *GhGPX6, GhGPX7* and *GhGPX8*, could even grow in medium containing 1 mM H_2_O_2_, although their OD_600_ were 0.001 (Fig. 8). This indicated that GhGPX6, GhGPX7 and GhGPX8 could be more effective scavengers of H_2_O_2_ than other GhGPXs, which could not grow in medium containing 1 mM H_2_O_2_ when the OD_600_ was 0.001.

## Discussion

Most plant defence proteins are encoded by families of closely related genes that usually display specific structural and regulatory features^48^. In this study, we performed genome-wide identification of the GPX family genes in *G. hirsutum*. The gene expression profiles of the leaves and roots of *GhGPX*s were investigated under high-salinity and hormone treatments to analyse their differences. Our results showed that the different members of the *GPX* gene family were co-ordinately regulated in different tissues under specific environmental stress conditions. The complementation analysis using the *gpx3Δ* cells in this study therefore provided evidence that the GhGPX1, GhGPX3, GhGPX5, GhGPX6, GhGPX7 and GhGPX8 play crucial role in scavenging of H_2_O_2_ in response to different stresses.

### Characterisation of GPXs from *G. hirsutum* and their evolution

Based on the scans of several plant genomes, GPX family genes have been systematically investigated from *A. thaliana*^36^,^37^, *L. japonicas*^38^, *P. trichocarpa*^5^ and *T. salsuginea*^35^. In this study, a total of 13 GPXs from *G. hirsutum* were identified and classified into four groups based on their phylogenetic clades (Fig. 3a). These 13 GhGPX proteins contained not only the three motifs present in most plant GPXs, but also three conserved cysteines (Fig. 2). GhGPX1 was shown to belong to clade 2, and is an enzyme localised to plastids (Table 4, Fig. 5), with 88% identity in its amino acid sequence compared with chloroplast-localised AtGPX1. The expression level of *GhGPX1* was also high in flowers and leaves, and it is consistent with the high concentrations of GSH in chloroplasts^1^ and its low levels in roots and stems, which suggested an important role for GPXs. This matches with previous report describing the involvement of AtGPX1 and AtGPX7 in the protection against photosynthesis-induced oxidative damage^28^. The plant GPXs are ubiquitous enzymes that have been shown to be present in different plant tissues, subcellular compartments and developmental stages^5^,^35^,^36^,^37^,^38^. ROS were produced at high levels in endoplasmic reticulum and peroxisome, surprisingly, no GhGPX was reported to target in these cellular compartments, it could be a possibility that the APX, CAT and SOD are the main antioxidant enzymes in these organs. Most of the 13 GhGPX proteins in terms of the evolutionary relationships were found to be closer to those of *P. trichocarpa*, revealed by phylogenetic analysis and in the same clade, each GhGPX was closer to PtGPX (Fig. 3b, marked in red and blue).

### The role of GPXs during plant growth and development

It had been reported that GPX genes were involved in not only plant growth and development, but also play very crucial role in the control of plant responses to abiotic stresses, biotic stresses and phytohormones^37^,^49^. For example, AtGPX1 and AtGPX7 contribute to cross-talk between photo-oxidative stress and immune responses, therefore they might be involved in defence against virulent pathogen infection^28^. AtGPX2 was also found to be part of a cytosolic protein complex involved in stress defence^50^. In addition, AtGPX3 plays a dual role in response to stresses: on one hand, it contributes to the maintenance of H_2_O_2_ homeostasis by the elimination of H_2_O_2_ and organic hydroperoxides; while on the other hand it works as a switch in the ABA and H_2_O_2_ signaling pathway in guard cells^27^; AtGPX8 plays an important role in the protection of cellular components including nuclear DNA against oxidative stress^29^. Moreover, *Atgpx1, Atgpx4, Atgpx6, Atgpx7* and *Atgpx8* mutants have been shown to have significantly larger lateral root density than the wild type, while the *Atgpx2* and *Atgpx3* mutants were embraced with significantly lower. The role of GPXs in the hormone-mediated (auxin, strigolactone and ABA) control of lateral root development has been documented^37^. In this study, we observed similar characters between GhGPXs and AtGPXs. First, the same number of exon-intron organisation, conserved Cys residues and GPX domains of GhGPXs and AtGPXs. Second, the different expression patterns of GPXs were also observed in cotton. The genes *GhGPX1, 3, 5, 8, 13* were expressed at high levels in leaves, of which the expression of *GhGPX1* being the highest; in addition, *GhGPX8* was expressed at a high level in stems and cotyledons. High expression levels of *GhGPX2, 3, 13*, were also found in roots. The tissue- or organ-specific GPX expression patterns indicated their functional divergence during plant development and growth. Moreover, the expression of all *GhGPX* genes induced by stress- or phytohormone-related signal treatment (Figs 6 and 7). Taken together, these results showed that GhGPXs are generally responsive to stress-related signals and phytohormone treatments, and play an important role in the responses to environmental stresses and the regulation of plant growth and development.

### The level of GhGPXs has antioxidant function

The plant GPX isoenzymes were demonstrated to detoxify H_2_O_2_ and organic hydroperoxides, and could be involved in maintenance of the thiol/disulphide or NADPH/NADP^+^ balance. Our results showed that the amount of GhGPXs could functionally complement a yeast mutant defective of *GPX3* gene (Fig. 8). Besides GHGPX2, the other GhGPXs such as GhGPX1, GhGPX3, GhGPX5, GhGPX6, GhGPX7 and GhGPX8 could functionally complement the H_2_O_2_-sensitive morphology in *gpx3Δ* yeast. AtGPX isoenzymes were able to utilize Trx as a sole electron donor for the reduction of H_2_O_2_ or hydroperoxides^51^, thiol-dependent redox regulation is more diverse in plants than in animals, bacteria or fungi^52^. These findings are consistent with the function of GPXs in catalysing reduction of H_2_O_2_. Therefore, further work to clone Trxs in *G. hirsutum*, which may act as downstream components of GhGPXs to regulate redox homostasis, is crucial and of great interest.

## Materials and Methods

### Plant materials and stress treatments

The seeds of cotton were wrapped in moist absorbent cotton and placed in a Petri dish, which were placed in an incubator for germinate with illumination at 25 °C for 3 days to germinate. The seedlings were grown in a sterile culture pots under long-day conditions (16 h/8 h light/dark photoperiod) with 26/20 °C day/night temperatures until the second true leaf appeared. Cotton seedlings were inoculated using the root dip method^53^. The root tissues were harvested at 24 h post-inoculation and >10 plants were collected per treatment.

### Isolation and cloning of *GhGPX* genes

We searched most of the *G. hirsutum* GPX gene sequences in the Cotton Genome Project database (http://cgp.genomics.org.cn/page/species/index.jsp) using the CDS of *GPX1–8* from *A. thaliana* with the BLAST tool, and searched GhGPX9, 10 from another database (http://mascotton.njau.edu.cn/html.Data/Genomefhsequence/2015/05/05/16ab0945-19e9-49f7-a09e-8e956ec866bf.html). The GhGPX genes were amplified by PCR (Fast Pfu DNA polymerase, Novoprotein) and the primers used for cloning and analysing the full-length CDS of *GhGPX* genes were designed by using Primer Premier 5 and their sequences were listed in Supplementary Table S1.

### Sequence alignments, phylogenetic analysis and other bioinformatic analyses

Isoelectric point (pI) and molecular weight (MW) predictions for each deduced *GhGPX* were performed using the Compute pI/MW tool: EXPASY (http://web.expasy.org/compute_pi/). The deduced sequences of 13 GhGPXs were aligned with the DNAMAN program (version 4.0; Lynnon Corporation, Pointe-Claire, Quebec, Canada) to identify conserved domains. These GhGPXs sequences were also used together with the sequences of the homologous proteins from *A. thaliana, O. sativa* and *P. trichocarpa*, to construct an unrooted phylogenetic tree by the neighbour-joining method using the ClustalW2.0 (http://www.ebi.ac.uk/Tools/msa/clustalw2/) and MEGA 6.0.5^54^ programs. For promoter analysis, 2 kb upstream region from translation start code ATG of each *GhGPXs* was from database of CGP^39^ and another database^41^, and then predicted *cis*-acting elements in (+) strand and (−) strand of each promoter of *GhGPXs* was found using the database of PlantCARE^42^ (Plant *Cis*-Acting Regualtory Element, http://bioinformatics.psb.ugent.be/webtools/plantcare/html/).

### Gene expression analysis

Total RNA was extracted by using TRIzol reagent (Invitrogen) from *G. hirsutum* seedlings. The cDNA was synthesised using M-MLV Reverse Transcriptase (Promega) and was 30-fold diluted with deionised water before use as a template in qRT-PCR. The quantitative reaction was performed on a 7500 Fast Real-Time PCR System (Applied Biosystems) using GoTaq^®^ qPCR Master Mix kit (Promega). Three independent biological and technical replicates were subjected to qRT-PCR analysis for each sample. The expression levels of *GhGPXs* were normalised against that of an internal reference gene, *GhUBQ7 (ubiquitin 7*, accession number: DQ116441). All primers used in this study are listed in Supplementary Table S1.

### Subcellular localisation of GhGPXs

For green fluorescent protein (GFP) analysis, the final construct *pHBT-GFP-GhGPXs* and empty vector *pHBT-GFP* were transiently expressed in *Arabidopsis* mesophyll protoplasts^55^. Transgenic cells were examined under an FV1000 (Olympus) confocal microscope.

### Fluorescence microscopy

Fluorescence was observed by using the laser scanning confocal microscope LSM FV1000 (Olympus). The fluorescence was excited at 488 nm and detected at 500–560 nm for GFP, while autofluorescence of chloroplasts was detected at 650–750 nm. Transmitted fields were collected simultaneously for use in merging images.

### Yeast complementation analysis

Full-length complementary DNA of *GhGPXs* was cloned into the vector *pYES2* and transformed into the wild type and *gpx3Δ* of *Saccharomyces cerevisiae* (genotype BY4741). Successfully transformed colonies were screened by complementation of leucine autotrophy. The presence of the correct plasmid was confirmed by PCR. The cell density of cultures was measured during exponential growth and was adjusted to OD_600_ 1.0, was analysed with the Biophotometer No. 6131–21283 (eppendorf, Germany). The growth assays were conducted at 30 °C. Yeast strains were precultured in liquid and solid yeast peptone dextrose medium. Yeast colonies grew on medium that containing 10% galactose and 10% raffinose. Primer sequences used in the PCR are presented in Supplementary Table S1.

## Additional Information

**How to cite this article:** Chen, M. *et al*. The Glutathione Peroxidase Gene Family in *Gossypium hirsutum*: Genome-Wide Identification, Classification, Gene Expression and Functional Analysis. *Sci. Rep.* **7**, 44743; doi: 10.1038/srep44743 (2017).

**Publisher's note:** Springer Nature remains neutral with regard to jurisdictional claims in published maps and institutional affiliations.

## Supplementary Material

Supplementary Data

## Figures and Tables

**Table 1:** The genomic locations of *GhGPX* genes.

Number	Sequence ID	Name	Location in genome
1	CotAD_10469	*GhGPX1*	Dt_chr1 42211918-42213928 (−)
2	CotAD_76066	*GhGPX2*	Dt_chr4 19846771-19848886 (−)
3	CotAD_22672	*GhGPX3*	Dt_chr4 21898946-21900968 (−)
4	CotAD_54994	*GhGPX4*	At_chr4 82962124-82964255 (−)
5	CotAD_58608	*GhGPX5*	At_chr4 89986498-89988499 (+)
6	CotAD_39880	*GhGPX6*	Dt_chr8 52575640-52576901 (+)
7	CotAD_39878	*GhGPX7*	Dt_chr8 52591449-52593319 (+)
8	CotAD_59095	*GhGPX8*	At_chr10 93484710-93486200 (+)
9	Gh_D12G2260	*GhGPX9*	D12 55725155-55726643 (+)
10	Gh_A12G2084	*GhGPX10*	A12 83692525-83694015 (+)
11	CotAD_39520	*GhGPX11*	At_chr13 80456382-80458243 (+)
12	CotAD_51884	*GhGPX12*	scaffold1637.1 220256-221724 (+)
13	CotAD_36707	*GhGPX13*	scaffold1048.1 502185-503584 (+)

**Table 2:** Homology analysis between GhGPXs and AtGPXs.

GHGPX	ATGPX	Identities	Positives
GHGPX1	ATGPX1	79%	88%
GHGPX2	ATGPX4	64%	77%
GHGPX3	ATGPX2	73%	85%
GHGPX4	ATGPX4	73%	86%
GHGPX5	ATGPX2	73%	85%
GHGPX6	ATGPX6	78%	85%
GHGPX7	ATGPX8	73%	89%
GHGPX8	ATGPX6	84%	93%
GHGPX9	ATGPX6	67%	76%
GHGPX10	ATGPX6	72%	80%
GHGPX11	ATGPX8	74%	90%
GHGPX12	ATGPX6	83%	93%
GHGPX13	ATGPX5	68%	82%

http://www.arabidopsis.org/wublast/index2.jsp.

**Table 3:** Putative *cis*-acting regulatory elements related to stress and hormone response in *GhGPXs*.

Environmental stress or hormone	*Cis*-acting regulator elements	Sequence	GhGPX
Abscisic acid (ABA)	ABRE	TACGTGCACGTG	GhGPX6,7,9,10GhGPX6,7,9,11
Auxin	TGA-element AuxRR-core	AACGACGGTCCAT	GhGPX4,5,6,8,9,10GhGPX1
Salicylic acid (SA)	TCA-element	CAGAAAAGGACCATCTTTTTGAGAAGAATATCAGAAGAGG	GhGPX1,7,8,11,12GhGPX1,8,10,13GhGPX3,5,11,13GhGPX11
Gibberellins (GA)	GARE-motifP-box	TCTGTTGAAACAGAGCCTTTTGAGTCCTTTTG	GhGPX2,5,13GhGPX1,2,6,9,10,11GhGPX2,6,10GhGPX2,6,9,10
Methyl Jasmonate(MeJA)	CGTCA/TGACG-motif	CGTCA/TGACG	GhGPX1,2,3,4,5,7,8,11,12
Ethylene	ERE	ATTTCAAA	GhGPX3,8,12,13
Drought inducibility	MBS	TAACTGCAACTGCGGTCA	GhGPX2,3,5,6,8,9,10,11,12GhGPX1,6,7,8,9,10,12GhGPX8
Low-temperature	LTR	CCGAAA	GhGPX2,4,8,13
Anaerobic induction	ARE	TGGTTT	GhGPX1,2,3,5,6,7,8,9,10,11

The tool to analyse cis-elements in the promoter of GhGPXs: Plant CARE (http://bioinformatics.psb.ugent.be/webtools/plantcare/html).

**Table 4:** Predicted properties of GhGPXs.

Protein	Length (aa)	MW (Da)	pI	Subcellular localisation	Putative homologues
*GhGPX1*	242	26645.51	9.29	Chlo	*AtGPX1*/*7, PtGPX1*
*GhGPX2*	171	19167.99	9.24	Cyt	*AtGPX4, PtGPX4*
*GhGPX3*	168	18847.42	5.66	Cyt	*AtGPX2, PtGPX2*
*GhGPX4*	171	19171.01	9.16	Cyt	*AtGPX4, PtGPX4*
*GhGPX5*	168	18948.44	5.36	Cyt	*AtGPX2, PtGPX2*
*GhGPX6*	170	18667.19	5.46	Cyt	*AtGPX6, PtGPX3.1/3.2*
*GhGPX7*	168	19129.61	4.59	Cyt	*AtGPX8, PtGPX5*
*GhGPX8*	166	18525.00	6.73	Cyt	*AtGPX6, PtGPX3.1/3.2*
*GhGPX9*	233	25489.14	8.62	Mito/Chlo	*AtGPX6, PtGPX3.1/3.2*
*GhGPX10*	233	25561.26	8.98	Mito/Chlo	*AtGPX6, PtGPX3.1/3.2*
*GhGPX11*	168	19292.91	5.10	Cyt	*AtGPX8, PtGPX5*
*GhGPX12*	166	18501.02	6.73	Cyt	*AtGPX6, PtGPX3.1/3.2*
*GhGPX13*	171	19187.91	8.84	Cyt	*AtGPX5, PtGPX4*

The tool to analyse the isoelectric point (pI) and molecular weight (MW) of *Gh*GPXs: EXPASY (http://web.expasy.org/compute_pi/). The tools to predict the subcellular localisation of *GhGPXs*: TargetP (http://www.cbs.dtu.dk/services/TargetP/) and SubLoc (http://www.bioinfo.tsinghua.edu.cn/SubLoc/).
