# Supplementary material for: The Glutathione Peroxidase Gene Family in Gossypium hirsutum: Genome-Wide Identification, Classification, Gene Expression and Functional Analysis
Source: Sci Rep. 2017 Mar 16;7:44743. doi: 10.1038/srep44743 (PMC5353742; doi:10.1038/srep44743)
Supplement: Supplementary Data [file srep44743-s1.pdf]

Title: The Glutathione Peroxidase Gene Family in *Gossypium hirsutum*: Genome-Wide Identification, Classification, Gene Expression and Functional Analysis

Authors: Mingyang Chen[1], Kun Li[1], Haipeng Li, Chun-Peng Song*, Yuchen Miao*

Affiliation: Institute of Plant Stress Biology, State Key Laboratory of Cotton Biology, Department of Biology, Henan University, 85 Minglun Street, Kaifeng 475001, China

*Corresponding authors

E-mail addresses: songcp@henu.edu.cn and miaoych@henu.edu.cn

Tel.: 86-371-23897139, Fax: 86-371-23881387

[1]These authors contributed equally to this work.

Author contributions: YM and C-PS conceived and supervised the study; YM designed the experiments; MC, KL and HL performed the experiments; YM provided new tools and reagents; MC, KL and YM analysed the data; KL and YM wrote the manuscript.

Supplementary data 1: the cloned gene sequences or gene sequences in database.

The cloned sequence of *CotAD_39521*

ATGGATCCCTCAAAGCAAGAAACAAACCAAAAAAAAGCTTCTAAACCGATGGCTAAGAAGGAAACCC
AAGAATTCCAATTTGTTGTTTACAATACCATGACTCAGCAAAAAGAGGTTTTCAAGCCCAAAACCCCT
GGAAAAGTTCGCATGTATGTCTGCGGTGTCACTGCCTATGATTTCAGTCACCTTGGCCATGCTCGTGCT
GCCGTTTCCTTTGATGTCCTTTACAGGTACCTTAAGCATTTGGGTTATGAGGTTACTTATGTCAGGAATT
TTACTGATGTTGATGATAAGATAATTCGTCGAGCCAATGAGACTGGAGAAGATCCAATAAGTTTGAGTG
ACCGGTACTGTAAAGAATATAATATAGATATGAGTGATCTACAGTGCCTTTCTCCAACACATGAGCCAC
GTGTTTCTGATCACATGGAGCAGATCAAAGATATGATAACTCAGATTATAAACAAAGACTTTGGATATG
TTGTTGACGGCGATGTGTTTTTTGCTGTTGATAAATTCCCAAATTATGGCAAGTTATCTGGGCAGAAGC
TGGAAAACAATAGAGCAGGTGAACGTGTTGCTGTTGACTCAAGGAAGCGTAATCCTTCCGACTTTGCA
TTATGGAAGGCTGCAAAGCCAGGTGAGCCATCTTGGGACAGCCCTTGGGGACCTGGACGACCTGGGT
GGCACATAGAATGTAGTGCAATGAGTGCACATTATTTAAGCTTCAAATTTGACATTCATGGTGGTGGGC
TTGATTTGATTTTTCCTCACCATGAAAATGAAATTGCTCAAAGTTGTGCTGCATGCGAAGAGAGTGACG
TAAGTTATTGGATGCACAATGGGCATGTTACTAATAACAATGAGAAGATGTCAAAATCTTTGGGTAACT
TTTTCACAATTCGCCAGATTACTGAGAGATATCATCCACTGGCTTTGCGATACTTCTTGATAAATGCACA
CTATCGTTCTCCTCTCAATTACTCCGTTGTTCAGTTGGAAGGTGCATCAGACGCTGTTTTTTACATATAT
CAGACTTTGAAAGATTGTCAAGATGCTCTATTGCAACTACAAGAAGAAATGCCCAACGATGGCAAACC
AGCTCGGATTACTCCGGACGCCAAGGAATGCATTAGCAAGCTCCGCAATGAGTTCCAGGTGAAAATGT
CAGATGACTTGAGCACTTCACTTATACTGACTGGAGCCTTTCTAGAAGCATTGAAGTTGGTAAACAATT
TGTTGACCATGCTAAAGAAGAAGCAGCAAAAGCAACAAAGATTATTGGTAATTCAATCCCTTAAAGAG
ATTGAGAACGAAGTTACAAAAGTTTTGGATGTTCTTGGATTGCAGCCACCTTGCTCTTATAACGAGGTT
TTGCTGCAATTAAAGGAGAAAGCATTAACGAGAGCCGGGTTGGTGGAAGATGATGTGATTCGTCTAAT
TAACGAGCGAGCTGAAGTGAGGAGAAACAAAGACTTCTTGAAAAGTGATCAGATGAGAGCTCATTTG
CAAGCAAAGGGCATTGCACTCATGGATGTAGGCACGGAAACAATTTGGAGACCTTGTGTTCCTGTTCA
ACAAGACTCGGAAATAGTGCCATCAGAGGGCCAGAAGGTCCCTCCCAAGCCTGAAAGTGCATAA

The sequence of *CotAD_39521* in a database

ATGCTCGTTCGACGAAATCTAAGTGCACTTGCTGTGTCTGCTTCTCTACTTTTAGGAAAGCGTTCCTCA
CCGAGTTTCAACCAAACCCTTTTGAGTTTTCCACAGATTTCACGAGTTTATCTGGTATCTCACTCGATC
AAAACAGGGTCTCCAAGGTCTCTCGTGGGTAGTTTGAGATTTGATCATACTATGGCTGCTCAGTCCTCC
AAGGGGTCAGTTCATGACTTCACTGTTAAGGATGCAAAAGGAAATGACGTCGATTTAAGCATTTACAA
GGGCAAGGTCCTCTTGATTGTCAATGTTGCATCACAATGTGGCTTGACCAACTCCAACTACACTGAGC
TGAGTAAATTGTACGAGAAATATAAAGATCAAGGTTTCGAGATTCTGGCATTTCCATGTAACCAGTTTG
GGGGTCAGGAGCCAGGGAACAATGAAGAAATCTTGGAGTTTGCTTGCACTCGCTTTAAAGCTGAATA
CCCCATATTTGATAAGGTGGATGTGAATGGCGAGAACGCAGCTCCGATATACAAATTCCTGAAGGCTAG
CAAAGGTGGAGCTCTGGGTGGACTTCTGGGGGACGACATCAAGTGGAATTTTGCCAAGTTCCTGGTG
GATAAAGACGGACATGTTGTGGATCGTTATGCCCCCACTACTTCCCCTATTAGCATCGAGGCTGCAAAG
CCAGGTGAGCCATCTTGGGACAGCCCATGGGGACCTGGACGACCTGGGTGGCACATAGAATGTAGTG
CAATGAGTGCACATTATTTAAGCTTCAAATTTGACATTCATGGTGGTGGGCTTGATTTGATTTTTCCTCA
CCATGAAAATGAAATTGCTCAAAGTTGTGCTGCATGCGAAGAGAGTGACGTAAGTTATTGGATGCACA
ATGGGCATGTTACTAATAACAATGAGAAGATGTCAAAATCTTTGGGTAACTTTTTCACAATTCGCCAGA
TTACTGAGAGATATCATCCACTGGCTTTGCGATACTTCTTGATAAATGCACACTATCGTTCTCCTCTCAA
TTACTCCGTTGTTCAGTTGGAAGGTGCATCAGACGCTGTTTTTTACATATATCAGACTTTGAAAGATTGT

CAAGATGCTCTATTGCAACTACAAGAAGAAATGCCCAACGATGGCAAACCAGCTCGGATTACTCCGGA
CGCCAAGGAATGCATTAGCAAGCTCCGCAATGAGTTCCAGGTGAAAATGTCAGATGACTTGAGCACTT
CACTTATACTGACTGGAGCCTTTCTAGAAGCATTGAAGTTGGTAAACAATTTGTTGACCATGCTAAAGA
AGAAGCAGCAAAAGCAACAAAGATTATTGGTAATTAAATCCCTTAAAGAGATTGAGAACGAAGTTACA
AAAGTTTTGGATGTTCTTGGATTGCAGCCACCTTGGTCTTATAACGAGGTTTTGCTGCAATTAAAGGAG
AAAGCATTGACGAGAGCCGGGTTGGTGGAGGATGATGTGATTCGTCTAATTAACGAGCGAGCTGAAGT
GAGGAGAAACAAAGACTTCTTGAAAAGTGATCAGATGAGAGCTCATTTGCAAGCAAAGGGCATTGCA
CTCATGGATGTAGGCACGGAAACAATTTGGAGACCTTGTGTTCCTGTTCAACAAGACTCGGAAATAGT
GCCATCAGAGGGCCAGAAGGTCCCTCCCAAGCCTGAAAGTGCATAA

The cloned sequence of *CotAD_54994* (*GhGPX4*)

Exon: — Intron: —

ATGGGTGCTTCTGAATCAGTTCCTCAGAAATCAATCCATGAATTCACTGTCAAGGTTCTTCTCTACCCTT
TACATTTCTTTGATAATTCCTCTTTCTTTTGATTGGGTTTTATGCTTTTGTTCTGTTGTTTGTGTTGCAGAA
TAGCAAAGGCCAGGACGTGGACCTTAGCATGTACAAAGGAAAGGTTCTCCTTGTGGTTAATGTTGCTT
CTAAATGGTCCTTTTCTACCATATCTTCTCTTCTTTGAACTCGTCGTCTTTGTGTTCTTTCCCGATTCTCA
ATTGTTTACCTTCATGATTTTAAACAGTGGGTTTACGGATTCAAATTACACCCAGTTGACTGAGCTTTAC
AACAAATACAAGGACAAAGGTTTGTTTCTCTTTGCTTAGCTGTGTATTTCATTTTTTTTTTTAGTTGCTT
AAAGCATATGAAATCAGCAACCATGACTGCGTAGTCTCCATAGCTAGTTCAAAGCTAACTCTTCATCTT
TTCTTAACACTCGAAAGCTCAAACCTTAGCTAAAATTGGTCGTTATTGAAAAAAGTTGCCGCTTCAAAT
TATGTCGAATATAACTTTATTTTAACCCTTTAAGAAAGGGTACAAATTACATAAGGAAACAAATTAATTT
TTATGCCAAAATCTACTTAAAAATCCCAATTGTGGAGGTCTAGTTTGGTTTATATCGTAGAGGGTAGTTT
GTAATGGGAACTAGCAGCTGGAACAGGACTGTGGAAGAAGAAAGGGAATGGGATTAGGGTTTTCTTT
TTGAGGAAAAATTTGCATTGAAAATGGACTTTAAACGGAAATTACATGTGCAGACCTGTGGAGCCAGA
TGACATTTAATTCACAATGATGGAAACTTTGACGCTAGATCAATGATTCAGAACTCTGTTATTAGTTTAG
TACTTGTGGAAGACCTTTCTTAAATTCAAGCATCCCTTTTGAGGCTTTTATTATTACTCTACATTTATTTT
TTCTTTTACCTAGTGGCTTTGTTCTTTTTCTCATAGCTCCGTAAACAAGTACCTTTTCAATTATCAGTTTA
GCCAATTTGATCATATGAAGCAAATGGTCGAACTATAGTTTTGCTTTCGGTTTTCTTCCCTAGATATGTTA
GGACTTGATGTTTTTTATTTTCTGATTGAAGGGTTGGAGATCTTGGCATTTCCTTGCAATCAGTTTTTGA
AGCAAGAACCAGGTACCAGCCAAGAGGCAGAAGAGTTTGCTTGCACAAGATACAAGGCTGAATATCC
TATTTTCAAAAAGGTAATGGGTTATATTTAGCATTGGATAATGGATCTCTTTTCTTAGAAGTAGCGCAAG
ATGTTTTTTCATTGAATATGATCATTGATTGGGTTAATTGTGATTGTAGTTGTTGATTGGATTTTGCCTTTT
TCGGATTAATTTAACAACTCTGTCGGGACTGCTACCATAAGAAGATAGAAATAGGTGGAACTATTGATA
GAACTCTGCTTCTTCATGAATAATATATGAACTTATTTGAGACTTTTCGTACATGTTGATGTTCCTTATGA
ATGTTTGTGATTGTTGTTGACTGGGATGGAGCTCAGCTCATTTGCTGACTATTTGTTTTCTATGTACATA
AACTAGCTGCATTTGGTTTGCATTCATTGCATCTTTAATGCCAGGGGGACTTTGCATTCTAACTGGTTAC
TGTTTGCTGCTAAAAATTTTATATATATAGGTTCGTTGCAACGGGCCGAATACAGAACCCGTCTTCAAGT
TCTTAAAAGCAAAGAAATCTGGTTTCTTGGGATCTAGGATAAAGTGGAACTTCACTAAGTTTTTAGTTG
ATAAGGATGGTAACGTCCTCTCTCGCTATAGCCCAACCACCACACCACTGGCCATAGAGGTATTCCCTA
CACCCTACTGGTGCCATTTTATCCTTGTTTCTTTTTAAATTTTTGAGGAGGGGGAATTGAACCCGAAGG
TGACCGTGCACCAACCGAGTCAAATGCTCAACTGCGGTGCATGTGTGCGAGACATTAATTTCGTGTTG
TGCTGCTTTGGTTGCTGTTGCAGGGTGACATCAAGAAAGCATTGGGAGTGGATACATGA

The sequence of *CotAD_*54994 (*GhGPX4*) in a database

Exon: — Intron: —

ATGGGTGCTTCTGAATCAGTTCCTCAGAAATCAATCCATGAATTCACTGTCAAGGTTCTTCTCTACCCTT
TACATTTCTTTGATAATTCCTCTTTCTTTTGATTGGGTTTTATGCTTTTGTTCTGTTGTTTGTGTTGCAGAA
TAGCAAAGGCCAGGACGTGGACCTTAGCATGTACAAAGGAAAGGTTCTCCTTGTGGTTAATGTTGCTT
CTAAATGGTCCTTTTCTACCATATCTTCTCTTCTTTGAACTCGTCGTCTTTGTGTTCTTTCCCGATTCTCA
ATTGTTTACCTTCATGATTTTAAACAGTGGGTTTACGGATTCAAATTACACCCAGTTGACTGAGCTTTAC
AACAAATACAAGGACAAAGGTTTGTTTCTCTTTGCTTAGCTGTGTATTTCATTTTTTTTTTTAGTTGCTT
AAAGCATATGAAATCAGCAACCATGACTGCGTAGTCTCCATAGCTAGTTCAAAGCTAACTCTTCATCTT
TTCTTAACACTCGAAAGCTCAAACCTTAGCTAAAATTGGTCGTTATTGAAAAAAGTTGCCGCTTCAAAT
TATGTCGAATATAACTTTATTTTAACCCTTTAAGAAAGGGTACAAATTACATAAGGAAACAAATTAATTT
TTATGCCAAAATCTACTTAAAAATCCCAATTGTGGAGGTCTAGTTTGGTTTATATCGTAGAGGGTAGTTT
GTAATGGGAACTAGCAGCTGGAACAGGACTGTGGAAGAAGAAAGGGAATGGGATTAGGGTTTTCTTT
TTGAGGAAAAATTTGCATTGAAAATGGACTTTAAACGGAAATTACATGTGCAGACCTGTGGAGCCAGA
TGACATTTAATTCACAATGATGGAAACTTTGACGCTAGATCAATGATTCAGAACTCTGTTATTAGTTTAG
TACTTGTGGAAGACCTTTCTTAAATTCAAGCATCCCTTTTGAGGCTTTTATTATTACTCTACATTTATTTT
TTCTTTTACCTAGTGGCTTTGTTCTTTTTCTCATAGCTCCGTAAACAAGTACCTTTTCAATTATCAGTTTA
GCCAATTTGATCATATGAAGCAAATGGTCGAACTATAGTTTTGCTTTCGGTTTTCTTCCCTAGATATGTTA
GGACTTGATGTTTTTTATTTTCTGATTGAAGGGTTGGAGATCTTGGCATTTCCTTGCAATCAGTTTTTGA
AGCAAGAACCAGGTACCAGCCAAGAGGCAGAAGAGTTTGCTTGCACAAGATACAAGGCTGAATATCC
TATTTTCAAAAAGGTAATGGGTTATATTTAGCATTGGATAATGGATCTCTTTTCTTAGAAGTAGCGCAAG
ATGTTTTTTCATTGAATATGATCATTGATTGGGTTAATTGTGATTGTAGTTGTTGATTGGATTTTGCCTTTT
TCGGATTAATTTAACAACTCTGTCGGGACTGCTACCATAAGAAGATAGAAATAGGTGGAACTATTGATA
GAACTCTGCTTCTTCATGAATAATATATGAACTTATTTGAGACTTTTCGTACATGTTGATGTTCCTTATGA
ATGTTTGTGATTGTTGTTGACTGGGATGGAGCTCAGCTCATTTGCTGACTATTTGTTTTCTATGTACATA
AACTAGCTGCATTTGGTTTGCATTCATTGCATCTTTAATGCCAGGGGGACTTTGCATTCTAACTGGTTAC
TGTTTGCTGCTAAAAATTTTATATATATAGGTTCGTTGCAACGGGCCGAATACAGAACCCGTCTTCAAGT
TCTTAAAAGCAAAGAAATCTGGTTTCTTGGGATCTAGGATAAAGTGGAACTTCACTAAGTTTTTAGTTG
ATAAGGATGGTAACGTCCTCATTTTTGAGGAGGGGGAATTGAACCCGAAGGTGACCGTGCACCAACC
GAGTCAAATGCTCAACTGCGGTGCATGTGTGCGAGACATTAATTTCGTGTTGTGCTGCTTTGGTTGCTG
TTGCAGGGTGACATCAAGAAAGCATTGGGAGTGGATACATGAAAATATAGGAGGTGGAGGGAAAATG
GAAACTAGAAACAATGTAAATAGGATGAAAGCGGTAGGCGTGGCCCATAATAATGTGTACCACTGTGA
GACCTCATGTGTAATGATAATCGCTGCTCGAGTTTGTAAAAAGTAG

The sequence of *Gh_A12G2084* (*GhGPX10*) in a database

ATGCTCGTTCGACGAAATCTAAGTGCACTTGCTGTGTCTGCTTCTCTACTTTTAGGAAAGCGTTCCTCA
CCGAGTTTCAACCAAACCCTTTTGAGTTTTCCACAGATTTCACGAGTTTATCTGGTATCTCACTCGATC
AAAACAGGGTCTCCAAGGTCTCTCGTGGGTAGTTTGAGATTTGATCATACTATGGCTGCTCAGTCCTCC
AAGGGGTCAGTTCATGACTTCACTGTTAAGGATGCAAAAGGAAATGACGTCGATTTAAGCATTTACAA
GGGCAAGGTCCTCTTGATTGTCAATGTTGCATCACAATGTGGCTTGACCAACTCCAACTACACTGAGC
TGAGTAAATTGTACGAGAAATATAAAGATCAAGGTTTCGAGATTCTGGCATTTCCATGTAACCAGTTTG
GGGGTCAGGAGCCAGGGAACAATGAAGAAATCTTGGAGTTTGCTTGCACTCGCTTTAAAGCTGAATA
CCCCATATTTGATAAGGTGGATGTGAATGGCGAGAACGCAGCTCCGATATACAAATTCCTAAAGGCTAG
CAAAGGTGGAGCTTTGGGTGGACTTCTGGGGGACGACATCAAGTGGAATTTTGCCAAGTTCCTGGTG
GATAAAGAGGGACATGTTGTGGATCGTTATGCCCCCACTACTTCCCCTATTAGCATCGAGTTGATATTTG

CAGAAGGATATAAAGAAACTGCTTGGCTGATGTTTTGGGCTTCGTCACCTCAGGCTTTGAGTGAATAA

The sequence of *Gh_D12G2260* (*GhGPX9*) in a database

ATGCTCGTTCGACGAAATCTAAGTGCACTTGCTGTGTCTGCTTCTCTACTTTTAGGAAAGCGTTCCTC
ACCGAGTTTCAACCAAACCCTTTTGAGTTTTCCACAGATTTCACCAGTTTATCTGGTATCTCACTCGAT
CAAAACAGGGTCTCCAAGGTCTCTCGTGGGTAGTTTGAGATTTGATCATACTATGGCTGCTGAGTCCT
CCAAGGGGTCAGTTCATGACTTCACTGTTAAGGATGCAAAAGGAAATGACGTCGATTTAAGCATTTA
CAAGGGCAAGGTCCTCTTGATTGTCAATGTTGCATCTCAATGTGGCTTGACCAACTCCAACTACACTG
AGCTGAGTAAATTGTATGAGAAATATAAAGATCAAGGTTTCGAGATTCTGGCATTTCCATGTAACCA
GTTTGGGGGTCAGGAGCCAGGGAACAATGAAGAAATCTTGGAGTTTGCTTGCACTCGCTTTAAAGCT
GAATACCCCATATTTGATAAGGTGGATGTGAATGGCGAGAACGCAGCTCCGATATACAAATTCCTGA
AGGCTAGCAAAGGTGGAGCTCTGGGTGGACTTCTGGGGGACGACATCAAGTGGAATTTTGCCAAGTT
CCTGGTGGATAAAGACGGACATGTTGTGGATCGTTATGCCCCCACTACTTCCCCTATTAGCATCGAGT
TGATATTTGCAGAAGGATATAAAGAAACTGCTTGGCTGATGTTTTGGGCTTCGTTACCTCAGGCTTTG
AGTGAATAA

Supplementary data 2: the CDS of *GhGPXs*.

>CotAD_10469 GhGPX1 (729 bp)
ATGGCTTCCATGTCTTTCTCTGCAACTATTCCGTCTCCTCTTCTTGATTTTTCCCAAACC
AAAAAGAACCCAGTTTTTTCTTCATCATGGCCTTCCATGTCTTTCTCAATTCCTTCCAT
CAAATCCTCGCTTGGGTCTTCAAAATCAGCCTTTTTCCAAAATGGGTTCTCCTTGCCAT
CGCTTACTGCTTCTGGGTTTGTCTTTAACTCTAGATCTTCAGGCATTTATGCAAGAGCA
GCTACGGATAAAACCCTATACGACTACACAGTAAAGGATATTGATGGGAAGGATACC
CCTCTTGGCAAATTTAAGGGAAAAGTTCTTTTGATTGTTAATGTTGCTTCAAGATGTG
GTTTGACAACATCAAATTACTCTGAGCTTTCCCACATATATGACAAGTACAAGAATCA
AGGATTTGAGATTCTAGCTTTCCCTTGCAATCAATTCGGGGGACAAGAGCCTGGATCA
AATCCTGATATTAAAAAATTTGCTTGTACCAGGTTCAAGGCAGAGTTTCCTATATTTG
ATAAGGTTGATGTGAATGGACCAAATACGGCTCCCGTTTACCAGTTTCTGAAGTCGAG
TGCTGGTGGTTTTTTCGGTGACCTGATCAAGTGGAACTTTGAGAAGTTCTTGGTGGAT
AAAAATGGCAAAGTTGTTGAGAGGTATCCACCAACGACATCGCCTTTCCAAATTGAG
AAGGACATTCAGAAGCTCCTCGCGACATGA

>CotAD_76066 GhGPX2 (516)
ATGGGTGCGTCTGAATCAGTTCCTCAGAAATCAATCCATGAATTCACTGTCAAGAATA
GCAAAGGCCAGGACGTGGACCTTAGCACGTACAAAGGAAAGGTTCTCCTTGTGGTTA
ATGTTGCTTCTAAATGTGGGTTTACGGATTCAAATTACACCCAGTTGACTGAGCTTTA
CAACAAATACAAGGACAAAGGGTTGGAGATCTTGGCATTTCCTTGCAATCAGTTTTTG
AAGCAAGAACCAGGTACCAGCCAAGAGGCAGAAGAGTTTGCTTGCAAAAGATACAA
GGCTGAATATCCTATTTTCAAAAAGGTTCGTTGCAACGGGCCGAATACCGAACCTGTC
TTCAAGTTCTTAAAAGCAAAGAAATCTGGTTTCTTGGGATCTAGGATAAAGTGGAACT
TCACTAAGTTTTTAGTTGATAAGGATGGTAACGTCCTCTCTCGCTATAGCCCAACCAC
CACACCACTGGCCATAGAGGGTGACATCAAGAAAGCATTGGGAGTGGATACGTGA

>CotAD_22672 GhGPX3 (507 bp)
ATGGCTGAAGACGCCTCCCCAGAATCCATTTACGACTTCAGTGTCAAGGACATTCGTG
GAAATGATGTAAGTTTAAGTGAATACAAAGGGAAAGTCGTTCTTATAGTGAATGTTG
CTTCAAAATGTGGTTTAACCCAATCAAACTATAAGGAACTCAATGTTTTGTATGAAAA
ATACAAAAACCAAGGATTTGAGATTTTAGCATTTCCTTGCAACCAGTTTGGGGGGCA
AGAACCAGGAACCAATGAACAAATTCAGGAAGCTACATGCTCAATGTTCAAAGCAGA
ATTTCCAATTTTTATCAAGGTCGAGGTAAATGGAAAGAATGCTGCACCTCTATACAGA
TTTCTAAAATCAGAAAAAGGCGGATACTTTGGAGATGCAATCAAGTGGAACTTCACA
AAGTTTTTGGTAAATAAAGAAGGCAAAGTTGTGGAGAGATATGCTCCAACCACATCA
CCCCTCAATATCGAGAAGGACATACGGGACCTACTCGGATCTTCTTAA

>CotAD_54994 GhGPX4 (516 bp)
ATGGGTGCTTCTGAATCAGTTCCTCAGAAATCAATCCATGAATTCACTGTCAAGAATA
GCAAAGGCCAGGACGTGGACCTTAGCATGTACAAAGGAAAGGTTCTCCTTGTGGTTA
ATGTTGCTTCTAAATGTGGGTTTACGGATTCAAATTACACCCAGTTGACTGAGCTTTA
CAACAAATACAAGGACAAAGGGTTGGAGATCTTGGCATTTCCTTGCAATCAGTTTTTG
AAGCAAGAACCAGGTACCAGCCAAGAGGCAGAAGAGTTTGCTTGCACAAGATACAA

GGCTGAATATCCTATTTTCAAAAAGGTTCGTTGCAACGGGCCGAATACAGAACCCGT
CTTCAAGTTCTTAAAAGCAAAGAAATCTGGTTTCTTGGGATCTAGGATAAAGTGGAAC
TTCACTAAGTTTTTAGTTGATAAGGATGGTAACGTCCTCTCTCGCTATAGCCCAACCAC
CACACCACTGGCCATAGAGGGTGACATCAAGAAAGCATTGGGAGTGGATACATGA

>CotAD_58608 GhGPX5 (507 bp)
ATGGCTGAAGACGCCTCCCCAGAATCCATTTACGACTTCACTGTCAAGGACATTCGTG
GAAATGATGTAAGTTTAAGTGAATACAGAGGGCAAGTTGTTCTTGTAGTGAATGTTGC
TTCAAAATGTGGTTTAACCCAATCAAACTATAAGGAACTCAATGTTTTGTATGAAAAA
TACAAAAACCAAGGATTTGAGATTTTAGCATTTCCTTGCAACCAGTTTGGGGGGCAA
GAACCAGGAACCAATGAACAAATTCAGGAAGCTACATGCTCAATGTTCAAAGCAGAA
TTTCCAATTTTTGATAAGGTCGAGGTAAATGGTAAGAATGCTGCACCTCTATACAAAT
TTCTAAAATCAGAAAAAGGCAGATACTTCGGAGATGCAATCAAGTGGAACTTCACAA
AGTTTTTGGTGAATAAAGAAGGCAAAGTTGTGGAGAGATATGCCCCAACCACATCAC
CCCTCAATATCGAGAAGGACATACGAGACCTACTCGGATCTTCTTAA

>CotAD_39880 GhGPX6 (513 bp)
ATGGCTGCTGAGTCCTCCAAGGGGTCAGTTCATGACTTCACTGTTAAGGATGCAAAA
GGAAATGACGTCGATTTAAGCATTTACAAGGGCAAGGTCCTCTTGATTGTCAATGTTG
CATCTCAATGTGGCTTGACCAACTCCAACTACACTGAGCTGAGTAAATTGTATGAGAA
ATATAAAGATCAAGGTTTCGAGATTCTGGCATTTCCATGTAACCAGTTTGGGGGTCAG
GAGCCAGGGAACAATGAAGAAATCTTGGAGTTTGCTTGCACTCGCTTTAAAGCTGAA
TACCCCATATTTGATAAGGTGGATGTGAATGGCGAGAACGCAGCTCCGATATACAAA
TTCCTGAAGGCTAGCAAAGGTGGAGCTCTGGGTGGACTTCTGGGGGACGACATCAAG
TGGAATTTTGCCAAGTTCCTGGTGGATAAAGACGGACATGTTGTGGATCGTTATGCCC
CCACTACTTCCCCTATTAGCATCGAGAAGGATATAAAGAAACTGCTTGGCTGA

>CotAD_39878 GhGPX7 (507 bp)
ATGACGACCCTAACTCCAAATAACCCAGACTCCGTCTATGCTTTTACTGTTAAGGATG
CTGAGGGAAACGATGTGGATCTCAATATTTACCAAGGAAAAGTGATGTTGATTGTTA
ATGTTGCTTCCAAATGTGGAATGACAAATTCCAACTACACAGAACTGAACCAATTAT
ACGAGAAGTATAAAGATCAAGGCTTGGAGATTCTTGCTTTTCCGTGCAATCAATTTGG
AGAGGAGGAACCAGGATCAAATGTTGAGATTTCTGAATTTGTTTGCACCTGCTTTAGA
TCGGAATTCCCTATCTTTGATAAGATTGAAGTAAATGGTGATAATGCCTCTCCACTGT
ACAAGTACTTAAAGTTGGGGAAATGGGGAATTTTCGGAGATGATATCCAATGGAACT
TCGCCAAGTTCTTGGTCAGTAAGGATGGTCAAGTTGTTCATCGTTATTACCCCACCAC
TTCTCCACTTAGTCTTGAGTATGATATAAAGAAGTTACTGGAATAA

>CotAD_59095 GhGPX8 (501 bp)
ATGGCTTCTCAATCTTCTAAGGGATCAGTTCATGATTTCACTGTTAAGGATGCAAGAG
GGAATGATGTTGATTTAAGTATTTACAAGGGCAAGGTTTTGTTGATTGTCAATGTTGC
ATCACAATGTGGCTTGACCAATTCCAACTACACTGAGCTAAGTAAATTGTATGAGCA
ATATAAAGATCAAGGTTTTGAGATTCTTGCATTCCCATGTAACCAGTTTGGAGGACAG
GAGCCAGGGAACAATGAGCAAATCTTAGAGTTTGCTTGCACTCGCTTTAAAGCTGAA

TACCCCATATTTGATAAGGTTGATGTGAATGGTGAGAAGGCTGCTCCCGTATACAAAT
TCCTCAAGTCTAGTAAAGGTGGACTTTTTGGGGACAGCATCAAGTGGAATTTTTCCAA
GTTCTTGGTCGATAAGGAGGGCCATGTCTTCGATCGTTATGCTCCCACTACTTCTCCTC
TTAGCATTGAGAAGGATATTAAGAAACTGCTGGCTTGA

>Gh_D12G2260 GhGPX9 (702 bp)
ATGCTCGTTCGACGAAATCTAAGTGCACTTGCTGTGTCTGCTTCTCTACTTTTAGGAA
AGCGTTCCTCACCGAGTTTCAACCAAACCCTTTTGAGTTTTCCACAGATTTCACCAGTT
TATCTGGTATCTCACTCGATCAAAACAGGGTCTCCAAGGTCTCTCGTGGGTAGTTTGA
GATTTGATCATACTATGGCTGCTGAGTCCTCCAAGGGGTCAGTTCATGACTTCACTGT
TAAGGATGCAAAAGGAAATGACGTCGATTTAAGCATTTACAAGGGCAAGGTCCTCTT
GATTGTCAATGTTGCATCTCAATGTGGCTTGACCAACTCCAACTACACTGAGCTGAGT
AAATTGTATGAGAAATATAAAGATCAAGGTTTCGAGATTCTGGCATTTCCATGTAACC
AGTTTGGGGGTCAGGAGCCAGGGAACAATGAAGAAATCTTGGAGTTTGCTTGCACTC
GCTTTAAAGCTGAATACCCCATATTTGATAAGGTGGATGTGAATGGCGAGAACGCAG
CTCCGATATACAAATTCCTGAAGGCTAGCAAAGGTGGAGCTCTGGGTGGACTTCTGG
GGGACGACATCAAGTGGAATTTTGCCAAGTTCCTGGTGGATAAAGACGGACATGTTG
TGGATCGTTATGCCCCCACTACTTCCCCTATTAGCATCGAGAAGGATATAAAGAAACT
GCTTGGCTGA

>Gh_A12G2084 GhGPX10 (702 bp)
ATGCTCGTTCGACGAAATCTAAGTGCACTTGCTGTGTCTGCTTCTCTACTTTTAGGAA
AGCGTTCCTCACCGAGTTTCAACCAAACCCTTTTGAGTTTTCCACAGATTTCACGAGT
TTATCTGGTATCTCACTCGATCAAAACAGGGTCTCCAAGGTCTCTCGTGGGTAGTTTG
AGATTTGATCATACTATGGCTGCTCAGTCCTCCAAGGGGTCAGTTCATGACTTCACTG
TTAAGGATGCAAAAGGAAATGACGTCGATTTAAGCATTTACAAGGGCAAGGTCCTCT
TGATTGTCAATGTTGCATCACAATGTGGCTTGACCAACTCCAACTACACTGAGCTGAG
TAAATTGTACGAGAAATATAAAGATCAAGGTTTCGAGATTCTGGCATTTCCATGTAAC
CAGTTTGGGGGTCAGGAGCCAGGGAACAATGAAGAAATCTTGGAGTTTGCTTGCACT
CGCTTTAAAGCTGAATACCCCATATTTGATAAGGTGGATGTGAATGGCGAGAACGCA
GCTCCGATATACAAATTCCTAAAGGCTAGCAAAGGTGGAGCTTTGGGTGGACTTCTG
GGGGACGACATCAAGTGGAATTTTGCCAAGTTCCTGGTGGATAAAGAGGGACATGTT
GTGGATCGTTATGCCCCCACTACTTCCCCTATTAGCATCGAGAAGGATATAAAGAAAC
TGCTTGGCTGA

>CotAD_39520 GhGPX11 (507 bp)
ATGACGACCCTAGCTCCAAATAACCCAGACTCCATCTATGCTTTTACTGTTAAGGATG
CTAAGGGAAACGATGTGGATCTCAATATTTACCGAGGAAAAGTGATGTTGATTGTTA
ATGTTGCTTCCAAATGTGGAATGACAAATTCCAACTACACAGAACTGAACCAATTAT
ACGAGAAGTATAAAGATCAAGGCTTGGAGATTCTGGCTTTTCCGTGCAATCAATTTGG
GGAGGAGGAACCAGGATCAAATGTTGAGATTTCTGAATTTGTTTGCACCCGCTTTAGA
TCGGAATTTCCTATCTTTGATAAGATTGAAGTAAATGGTGATAATGCCTCTCCACTGT
ACAAGTACTTAAAGTTGGGGAAATGGGGAATTTTCAGAGATGATATCCAATGGAACT
TCGCCAAGTTCTTGGTCAGTAAGGATGGCCAAGTTGTTCATCGTTATTACCCCACCAC

TTCTCCACTTAGTCTTGAGTATGATATAAAGAAGTTACTGGAATAA

>CotAD_51884 GhGPX12 (501 bp)
ATGGCTTCTCAACCTTCTAAGGGATCAGTTCATGATTTCACTGTTAAGGATGCAAGAG
GGAATGATGTTGATTTAAGTATTTACAAGGGCAAGGTTTTGTTGATTGTCAATGTTGC
ATCACAATGTGGCTTGACCAATTCCAACTACACTGAGCTGAGTAAATTGTATGAGCA
ATATAAAGATCAAGGTTTTGAGATTCTTGCATTCCCATGTAACCAGTTTGGAGGACAG
GAGCCAGGGAACAATGAGCAAATCTTAGAGTTTGCTTGCACTCGCTTTAAAGCTGAA
TACCCCATATTTGATAAGGTCGATGTGAATGGTGAGAAGGCTGCTCCCATATACAAAT
TCCTCAAGTCTAGTAAAGGTGGACTTTTTGGGGACAGCATCAAGTGGAATTTTTCCAA
GTTCTTGGTCGATAAGGAGGGCCATGTCGTCGATCGTTATGCTCCCACTACTTCTCCT
CTTAGCATTGAGAAGGATATAAAGAAACTGCTGGCTTGA

>CotAD_36707 GhGPX13 (516 bp)
ATGGGTGCTTCTGAATCAGTTCCTCAAAAATCAATCCATGAATTTACAGTCAAGGATTA
CAAAAACCAAGATGTTGACCTTAGTATGTACAAAGGAAAAGCTCTTCTTGTTGTTAATG
TTGCCTCTAAATGTGGACTCACTGATTCAAATTATACCCAGTTGACTGATCTTTACAACA
AATACAAGGACCAAGGACTGGGGATTTTGGCATTCCCATGCAATCAGTTTTTGAGCCA
AGAACCTGGTACCAGTCAAGATGCACAAGAATTTGCTTGTACAAGATACAAGGCTGAA
TACCCTATTTTCCAAAAGGTTCATGTCAATGGTCCAAAGACAGAGCCCGTCTACAAGTT
CTTAAAGACAAACAAATCTGGGTTCTTGGGAAATAGGATAAAGTGGAACTTCACTAAG
TTTTTAGTTGACAAGGATGGCCATGTCCTTGCTCGCTATGGCCCTAGCACTACGCCATT
GGCCATTGAGGCTGACATCAAGAAGACTTTGGGAGTGGACATGTGA

Supplementary data 3: the open reading frames of the 13 *GhGPXs*.
Exon: — Intron: —

>GhGPX1 CotAD_10469 (1678 bp)
ATGGCTTCCATGTCTTTCTCTGCAACTATTCCGTCTCCTCTTCTTGATTTTTCCCAAACG
AAAAAGAACCAAGTTTTTTCTTCATCATGGCCTTCCATGTCTTTCTCAATCCCTTCCATC
AAATCCTCGCTTGGGTCTTCAAAATCAGCCTTTTTCCAAAATGGGTTCTCGTTGCTATC
GCTTACTGCTTCTGGGTTTGTCTTTAACTCTAGATCTTCAGGCATTTATGCAAGAGCAGC
TACGGATAAAACCCTATACGACTACACAGTAAAGGTACAAGCTTGGGGAATATTACGTT
GAACAAAAGTTCCAATTTTGTTACTGTATTTTGCTAAAGGATATCTCTCTCTATCTTTTTG
AGCAGGATATTGATGGGAAGGATACCCCTCTTAGCAAATTTAAGGGAAAAGTTCTTTTG
ATTGTTAATGTTGCTTCAAGATGGTATATCATCACAATATTTTCTAGGGCTGCTTTGAGCT
TTTGTCTTTTTTTTTGTTGGTCTAATTGAAATTTTTTTCATTTTGATTCATAGTGGTTTGAC
AACATCAAATTACTCTGAGCTTTCCCACATATATGACAAGTACAAGAATCAAGGTCAGT
TAAGCATTCTTGCCTTGATATTTTGTTCCTTAATAATGTCATCATGTTTTTGTAAGATTTG
TTGATAAAACTCTATCACAGGATTTGAGATTCTAGCTTTCCCTTGCAATCAATTCGGGGG
ACAAGAGCCTGGATCAAATCCTGATATTAAAAAATTTGCTTGTACCAGGTTCAAGGCAG
AGTTTCCTATATTTGATAAGGTGAGTCAAGCTGCTCTTCTATTTGATTATAGCATTTACGG
TAGAGATAAGTTCTTGAAGGACTAAAGGAAGTTTTATGCATCAGGTTGATGTGAATGGA
CCGAATACGGCTCCCGTTTACCAGTTTCTGAAGTCGAGTGCTGGTGGATTTTTCGGTGA
CCTGATCAAGTGGAACTTTGAGAAGTTCTTGGTGGATAAAAATGGCAAAGTTGTTGAG
AGGTATCCACCAACGACATCGCCTTTCCAAATTGAGGTATGCAAGTTAATCTCAACACT
TGAAGCACTAGGGTTTTGATAACATGAAATCAAGTATGATTTAAGATTCTTTGTCTTAAG
GCTTGTAATGAAAGATCAATGTTGATACTTATAAAATTTATCATTTGTTTGCTGTTTGATT
GCTATATATCTTGAGAAAGAACTGGTGGCTTTTTGGATTGTGGATTGATATCAAACATAT
TTTTCTACTATATTCGATGCTCTTCTCTTTTCGGGGCATTGAAATGCCGACATTGTATGCA
ACTCTCTTGTCTGATAGAACATCCAGAACAGTTATTATCTTACTGGTTTATTCTGGTATC
AGATTTGGTGTTGCAATCAAACTCAAAAGCTGTGTTACTGTCAAGTAATACCACTATGT
TGGATTTGAAGTGGTTTGTTCTAGTCTTCCCTGTTTTCACCGCTAGGTCGAATCTTTTTT
TGTACTCGGTGCAATAGATACCGAACATTGACAACGAAAACATGTCTCTTTGTTTTGAT
TTGGTCGTTTTATTCTTTTGTTATTTTGGCCCTGAATCTCAGAATATGATTCAACGGACTA
ATTCTTGTTCTTATCATGTGATGGTGTTGCAGAAGGACATTCAGAAGCTCCTCGCGACA
TGA

GhGPX1	E1	I1	E2	I2	E3	I3	E4	I4	E5	I5	E6
bp	273	91	77	90	62	87	119	85	168	596	30

>GhGPX2 CotAD_76066 (2081 bp)
ATGGGTGCGTCTGAATCAGTTCCTCAGAAATCAATCCATGAATTCACTGTCAAGGTTCT
TCTCTACCCGTTACATTTCTTTGATAATTCCTCTTTCTTTTGATTGGGTTTTATGCTTTTGT
TTGGTTGTTTGTGTTGCAGAATAGCAAAGGCCAGGACGTGGACCTTAGCACGTACAAA
GGAAAGGTTCTCCTTGTGGTTAATGTTGCTTCTAAATGGTCCTTTTCTACCTTTCATATC
TTCTCTTCTTTGAACTCGTCGTCTTTGTTTTCTTTCCCGATTCTAAATTGTTTACCTTTAT
GATTTTAAACAGTGGGTTTACGGATTCAAATTACACCCAGTTGACTGAGCTTTACAACA

AATACAAGGACAAAGGTTGGTTTCTCTTTGCTTAGCTGTGTATTTCTTTTTTTTTAGTTG
CTTAAAGCTTATGAAATCAGCAACCATGACTGCACAGTCTGCATAGCCAGTTCAAAGCT
AACTCTTCATCTTTTCTTAACACTCGAAAGTTCAAACCTTAGCTAAAATTGGACGTTATT
GCAAAAAGTTGCCGCTTCAAATTATGTCGAATGTAACTTTATTTTAACCCTTTAAGAAA
GGGTACAAATTACATAAGGAAACAAATTAATTTTTATGCCAAAATCTACTTAAAAATCCC
AATTGTGGAGTTCTAGTTTGGTTTAAATCGTAGAGGGTAGTTTGTAATAGGAACTAGCA
GCCGGAACAGGACTGTGGAAGAAGAAAGGGAATGGGATTAGGGTTTCTTTTTGAGG
AAAAATTTGCATTGAAAATGGACTTTAAACTGAAATTACATGTGCAGACCTGTGGAGC
CAGATGACATTTAATTCACAATGATGGAAACTTTGACGGTAGATCAATGATTCAGAACT
CTGTTATTAGTTTAGTACTTGTGGAAGACCTTTCTTAAATTCAAGCATCCCTATAGAGGC
TTCTATTATTACTCGACATTTATTTTTTCTTTTCCCTAGTGGCTTTGTACTTTTTCTCATAG
CTCTGTAAACAAATACCTTTTCAATTATCAGTTTAGCCAATTTGATCATAAGAAAAAAAT
GGTCGAATTATAGTTTTGCTTTCGGTTTTCTTCCCTAGATATGTTAGGACTTGATGTTTTT
TATTTTCTGATTGAAGGGTTGGAGATCTTGGCATTTCCTTGCAATCAGTTTTTGAAGCA
AGAACCAGGTACCAGCCAAGAGGCAGAAGAGTTTGCTTGCAAAAGATACAAGGCTGA
ATATCCTATTTTCAAAAAGGTAATGGGTTATATTTAGCATTGGATAATGGATCTCTTTTCT
TAGAAGTAGCGCAAGATGTTTTTTCATTGAATATGATCATTGATTGGGTTAATTGTGATT
GTAGTTGTTGATTGGATTTTGCCTTTTTCGGATTAATTTAACAACTCTGTCGGGACTGCT
ACCATAAGAAGATAGAAATAGGTGGAACTATTGATAGAACTCTGCTTCTTCATGAATAAT
ATATGAACTTATTTGAGACTTTTTGTACATGTTGATGTTCCTTGTGAATGTTTGTGATTGT
TGTTGACTGGAATGGAGGTCAGCTCATTTGCTGAGTCTTTGTTTTCTATGTACATAAACT
AGCTGCATTTGGTTTGCATTCATTGCATCTTCAATTCCAGGGGGACTTTGCATTTTAACT
GGTTACTGTTTGCTGCTAAAAATTTTATATATATAGGTTCGTTGCAACGGGCCGAATACC
GAACCTGTCTTCAAGTTCTTAAAAGCAAAGAAATCTGGTTTCTTGTGATCTAGGATAAA
GTGGAACTTCACTAAGTTTTTAGTTGATAAGGATGGTAACGTCCTCTCTCGCTATAGCC
CAACCACCACACCACTGGCCATAGAGGTATTCCCTACACCCTACTGGTACCATTTTATC
CTTGTTTCTTTTTAATTTTTTGAGGAGGGGGAATTGAACCCGAAGGTGACCATGCACCA
ACCGAGTCAAATGCTCAACTGCGGTGCATGTGTGCGAGACATTAATTTCGTGTTGTGCT
GCTTTGGTTGCTGTTGCAGGGTGACATCAAGAAAGCATTGGGAGTGGATACGTGA

GhGPX2	E1	I1	E2	I2	E3	I3	E4	I4	E5	I5	E6
bp	54	86	77	95	62	775	119	439	168	170	36

>GhGPX3 CotAD_22672 (2023 bp)
ATGGCTGAAGACGCCTCCCCAGAATCCATTTACGACTTCAGTGTCAAGGTATTCATTTC
TTCTTTTTTAGCAATTTGGATACCCATCCACTAATACTATGTCAATGTTGGGAGTGGGGA
TTGAATTTTATTTATTTTTATCCTTTAAAACTTGAATTACTTACTTCCCCCAAATTTTGTTT
ATTTGCTGCTGGGCCTGGATTTGTATTACTGTCGAATTACTTGTTGCTTTACTCTCTGCG
TGAATTTATCATCGCTCTTATTAACTATTGATCATGCGCATCAATATCTGTTTAATAAAGG
GGGAAAGGAAGTTAAAGCTTTGTTGATGTGCTTCGACTTTGCCGATAGATTTGGGAGT
TCACAGGTCAAGGAGTACATCAACAAGTCCGGTATTATACTCGATTGAGGCTTTGTTTT
CCATCTTTGCCAAGAGATTTACGAGTTCACAAAGTAGGAGCCTCTCGTGTTGCCTCAA
CAGGTATAAGCCATGAATGCGGCACATGGTTTCTGAAAAGATAGCTTATGGCAAGGGTC
TTGGCCTAGCTAGCTCAAGTGTTCCCTATAAATGATTTGTATGCCATAGGTATGGGGTTA

AGTCCAATGTGTGCAAATTCCTAGTTTGCTATAGTTTTGCCTGTTCAGGCATTTTGAGGC
ACTGTCATTATGTGAACTCATCAGGACTGTTAGTAGAGTAAGAAAGACAAAACCCCGA
CCGAAGATAATATTTGACTTGTGTGATATACCCTTTGGTCTGTAAACCTGTAGGGCTCTC
GGTAGAACTGAGGCACGTCAACAAAGTTTCAACTGAATAAACTTGATTTTTTACAATCT
GCAGGACATTCGTGGAAATGATGTAAGTTTAAGTGAATACAAAGGGAAAGTCGTTCTT
ATAGTGAATGTTGCTTCAAAATGGTAATCTGCTTGATCAGTTATATGGCCTGATTTTTTA
GCATAACTCTTCATCAATGACTTACACTTCATAGCTATTCATGTTGAATATCATTTTTCTC
CATTTGGCAGTGGTTTAACCCAATCAAACTATAAGGAACTCAATGTTTTGTATGAAAAA
TACAAAAACCAAGGTCTGTCATTTGTCATAAATTGGAGACCTTTTTATGTTCTGTCTAAT
TGTTTGAATTGAAGCTTATAAATGTTATGCAGGATTTGAGATTTTAGCATTTCCTTGCAA
CCAGTTTGGGGGGCAAGAACCAGGAACCAATGAACAAATTCAGGAAGCTACATGCTC
AATGTTCAAAGCAGAATTTCCAATTTTTATCAAGGTTGTAACCATGTATACGCGTACAAT
CTGTTATGAATTTTTATCTGTTGTTAACGTTTATTGTATTATTCATTCTTTGCTTTTGGATC
AAAGTGTACTTCTTTTAGGTCCTCTTACAATCAACAATGTAGTCTATTGAACTACAAATA
TAGCTTTCCCCTCTATGGCTCTATCTTATCCCACACTACTTATGCCAGCAATATGGTGACC
TAGCAGGAAAATAATTAGCCTCCTTAGCATGAAAATGGAACCTATGTCATAACACACAA
ATGAGTGCACTTTGCAAAACGGGTGTCAATTGATCTACACAAGCCATATCCAATGAGCT
GGGTCGGGTTGTGAGAATATGACCGTGACACAAAAGTTTCTCTCTTTAGAGAAGAGTT
GAAAGTCGATAAATATGCATACAAACCTATGGTTTCTTATACATCATATCTGACTATTGCA
GGTCGAGGTAAATGGAAAGAATGCTGCACCTCTATACAGATTTCTAAAATCAGAAAAA
GGCGGATACTTTGGAGATGCAATCAAGTGGAACTTCACAAAGTTTTTGGTAAATAAAG
AAGGCAAAGTTGTGGAGAGATATGCTCCAACCACATCACCCCTCAATATCGAGGTTAG
AGTTGTTCTATTTTTCTGATAACATTTGATACCTGTAAAAATGTATAAACTCCATTAAATC
GTTCCTTGTTACATTTCTCTCTTGCAGAAGGACATACGGGACCTACTCGGATCTTCTTAA

GhGPX3	E1	I1	E2	I2	E3	I3	E4	I4	E5	I5	E6
bp	48	789	77	108	62	79	119	447	168	93	33

>GhGPX4 CotAD_54994 (2080 bp)

ATGGGTGCTTCTGAATCAGTTCCTCAGAAATCAATCCATGAATTCACTGTCAAGGTTCT
TCTCTACCCTTTACATTTCTTTGATAATTCCTCTTTCTTTTGATTGGGTTTTATGCTTTTGT
TCTGTTGTTTGTGTTGCAGAATAGCAAAGGCCAGGACGTGGACCTTAGCATGTACAAA
GGAAAGGTTCTCCTTGTGGTTAATGTTGCTTCTAAATGGTCCTTTTCTACCATATCTTCT
CTTCTTTGAACTCGTCGTCTTTGTGTTCTTTCCCGATTCTCAATTGTTTACCTTCATGATT
TTAAACAGTGGGTTTACGGATTCAAATTACACCCAGTTGACTGAGCTTTACAACAAATA
CAAGGACAAAGGTTTGTTTCTCTTTGCTTAGCTGTGTATTTCATTTTTTTTTTTAGTTGC
TTAAAGCATATGAAATCAGCAACCATGACTGCGTAGTCTCCATAGCTAGTTCAAAGCTA
ACTCTTCATCTTTTCTTAACACTCGAAAGCTCAAACCTTAGCTAAAATTGGTCGTTATTG
AAAAAAGTTGCCGCTTCAAATTATGTCGAATATAACTTTATTTTAACCCTTTAAGAAAG
GGTACAAATTACATAAGGAAACAAATTAATTTTTATGCCAAAATCTACTTAAAAATCCCA
ATTGTGGAGGTCTAGTTTGGTTTATATCGTAGAGGGTAGTTTGTAATGGGAACTAGCAG
CTGGAACAGGACTGTGGAAGAAGAAAGGGAATGGGATTAGGGTTTTCTTTTTGAGGA
AAAATTTGCATTGAAAATGGACTTTAAACGGAAATTACATGTGCAGACCTGTGGAGCC
AGATGACATTTAATTCACAATGATGGAAACTTTGACGCTAGATCAATGATTCAGAACTC

TGTTATTAGTTTAGTACTTGTGGAAGACCTTTCTTAAATTCAAGCATCCCTTTTGAGGCT
TTTATTATTACTCTACATTTATTTTTTCTTTTACCTAGTGGCTTTGTTCTTTTTCTCATAGC
TCCGTAAACAAGTACCTTTTCAATTATCAGTTTAGCCAATTTGATCATATGAAGCAAATG
GTCGAACTATAGTTTTGCTTTCGGTTTTCTTCCCTAGATATGTTAGGACTTGATGTTTTTT
ATTTTCTGATTGAAGGGTTGGAGATCTTGGCATTTCCTTGCAATCAGTTTTTGAAGCAA
GAACCAGGTACCAGCCAAGAGGCAGAAGAGTTTGCTTGCACAAGATACAAGGCTGAA
TATCCTATTTTCAAAAAGGTAATGGGTTATATTTAGCATTGGATAATGGATCTCTTTTCTT
AGAAGTAGCGCAAGATGTTTTTTCATTGAATATGATCATTGATTGGGTTAATTGTGATTG
TAGTTGTTGATTGGATTTTGCCTTTTTCGGATTAATTTAACAACTCTGTCGGGACTGCTA
CCATAAGAAGATAGAAATAGGTGGAACTATTGATAGAACTCTGCTTCTTCATGAATAATA
TATGAACTTATTTGAGACTTTTCGTACATGTTGATGTTCCTTATGAATGTTTGTGATTGTT
GTTGACTGGGATGGAGCTCAGCTCATTTGCTGACTATTTGTTTTCTATGTACATAAACTA
GCTGCATTTGGTTTGCATTCATTGCATCTTTAATGCCAGGGGGACTTTGCATTCTAACTG
GTTACTGTTTGCTGCTAAAAATTTTATATATATAGGTTCGTTGCAACGGGCCGAATACAG
AACCCGTCTTCAAGTTCTTAAAAGCAAAGAAATCTGGTTTCTTGGGATCTAGGATAAA
GTGGAACTTCACTAAGTTTTTAGTTGATAAGGATGGTAACGTCCTCTCTCGCTATAGCC
CAACCACCACACCACTGGCCATAGAGGTATTCCCTACACCCTACTGGTGCCATTTTATC
CTTGTTTCTTTTTAAATTTTTGAGGAGGGGGAATTGAACCCGAAGGTGACCGTGCACC
AACCGAGTCAAATGCTCAACTGCGGTGCATGTGTGCGAGACATTAATTTCGTGTTGTGC
TGCTTTGGTTGCTGTTGCAGGGTGACATCAAGAAAGCATTGGGAGTGGATACATGA

GhGPX4	E1	I1	E2	I2	E3	I3	E4	I4	E5	I5	E6
bp	54	86	77	91	62	778	119	439	168	170	36

>GhGPX5 CotAD_58608 (2002 bp)
ATGGCTGAAGACGCCTCCCCAGAATCCATTTACGACTTCACTGTCAAGGTAT
ATATTTCTTCTTTTTTAGCAATTTGGATACCCATCCACTAATACTATGTCTATG
TTGGGAGTGGGGATTGAATGTTATTTATTTTTATCCTTTAAAACTTGAATTAC
TTACTTCCCCCAAATTTTGTTTATTTGCTGCTGGGCCTGGATTTGTATTACTG
TCGAATTACTTGTTGTTTTACTCTCTGCGTGTATTTATCATCGCTCTTATTAAC
TATTCATCATGCGCATCGATATCTGTTTAATAAAGGGGAAAAGGAAGTTAAA
GCATTGTTGGTGTGCTTTGACTTTGCCGATAGCTTGGGAGTCACAGGTCGA
GGAGTATATCAACAAGTCCGGTATTATCCTCGATTGAGGCTTTGTTTCCATCT
TTGCCAAGAGATTTACGAGTTCACAAAGTAGGAGCCTCTCGTGTTGCCTCA
ACAGGTATAAGCCATGAATGCGGCACATGGTTTCTGAAAAGATAGCTTATG
GCAAGGGTCTTGGCCTAGCTAGCTCAAGTGTTCCCTATAAATGATTTATATG
CCATAGGTATGGGGTTAAGTCCAATGTGTGCAAATTCCTAGTTTGCTATAGT
TTTGCCTGTTTAGGCATTTTGAAGCACTGTCATTATGTGAACTCATCAGAAC
TGTTAGTAGAGTCAGAAAGACAAAACCCCGGCCGAAGATAATATTTGACTT
GTGTGATATACCCTTTGGCTCTCGGTAGAACTGAGGCACGTCAACAAAGTT
TCAACTGAATAAACTTCAATTTTTACAATCTGCAGGACATTCGTGGAAATGA
TGTAAGTTTAAGTGAATACAGAGGGCAAGTTGTTCTTGTAGTGAATGTTGC
TTCAAAATGGTAATCTGCTCGATCAGTTATATGGCCTGATTTTTTAGCATAAC
TCTTCATCAATGACTTACACTTCATAGCTATTCATGTTAAATATCATTTTTCTC

CATTTGGCAGTGGTTTAACCCAATCAAACTATAAGGAACTCAATGTTTTGTA
TGAAAAATACAAAAACCAAGGTCTGTCATTTGTCATAAATTGGAGACCTTT
TTATGTTCTGTCTAATTGTTTGAATTGAAGCTTATAAATGTTATGCAGGATTT
GAGATTTTAGCATTTCCTTGCAACCAGTTTGGGGGGCAAGAACCAGGAACC
AATGAACAAATTCAGGAAGCTACATGCTCAATGTTCAAAGCAGAATTTCCA
ATTTTTGATAAGGTTGTAACCACGTAGACGCGTGAAATCTGTTATGAATATTT
ATCCGTTGTTAACGTTTATTGTATTATTCATTCTTTGCTTTTGGATCAAAGTGT
ACTTCTTTTAGGTCCTCTTACAATCAACAATGTAGTCTGTTGAACTACAAAT
ATGGCTTTCCCCTCTATGGCTCTATCTTATCCCACACTACTTATGCCAGCAAT
ATGGTGACCTAGCAGGAAAATAATTAGCCTCCTTAGCATGAAAATGGGACC
TATGTCATAACACACAAATGAGTGCACTTTGCAAAACGGGTGTCAATTGAT
CTACACAAGCCATATCCAATGAGCTGGGTCGGGTTGTAAGAATATGACTGT
GACACAAAAGTTTCTCTCTTAGAGAGGAGTTGAAAGTCGATAAATATGCAT
ACAAACCTATGGTTTCTTATACATCATATCTAACTATTGCAGGTCGAGGTAAA
TGGTAAGAATGCTGCACCTCTATACAAATTTCTAAAATCAGAAAAAGGCAG
ATACTTCGGAGATGCAATCAAGTGGAACTTCACAAAGTTTTTGGTGAATAA
AGAAGGCAAAGTTGTGGAGAGATATGCCCCAACCACATCACCCCTCAATAT
CGAGGTTAGAGTTGTTTTATTTTTCTGATAACATTTGATACCTGTAAAAATGT
ATGAACTCCATTAAATCGTTCCTTGTTACATTTCTCTCTTGCAGAAGGACAT
ACGAGACCTACTCGGATCTTCTTAA

GhGPX5	E1	I1	E2	I2	E3	I3	E4	I4	E5	I5	E6
bp	48	769	77	108	62	79	119	446	168	93	33

>GhGPX6 CotAD_39880 (1262 bp)

ATGGCTGCTGAGTCCTCCAAGGGGTCAGTTCATGACTTCACTGTTAAGGTTGGTTTTGG
GTATAAATTTTACCCCCACAGACGAAGTTTATTTGATTCAAATTTAGTTTTGTCTTTGATT
CAATTTTCAATGTCCCTCCCCTCCTTAAGATATGGGTTTGGTTCTTCAGGATGCAAAAG
GAAATGACGTCGATTTAAGCATTTACAAGGGCAAGGTCCTCTTGATTGTCAATGTTGCA
TCTCAATGGTACCTCTAATTTCAATTTTGTGTAACAAGGATATAGTGGATTGATTGCACA
TTTAGATTATGCTTTTCTTTGGCCTACTTGTTGGATTTTTAGCTTTAATTTCTGGACAGTT
TTGAATTTGGTGCTTAAAATGTTGATGCAGTGGCTTGACCAACTCCAACTACACTGAGC
TGAGTAAATTGTATGAGAAATATAAAGATCAAGGTTTGTTTTAGCATCCTTGTAGCTTCT
TGTTTCTGAGGACCATTCATGAGCCTTTAGTTGATTTCAGTGAAGTGAGGTGTAGACAG
CACTTGAAGTATCATGGAACTGCCATTTGATTATTTGATTTGCTGGTTTGTCGGGTCAGG
TTTCGAGATTCTGGCATTTCCATGTAACCAGTTTGGGGGTCAGGAGCCAGGGAACAAT
GAAGAAATCTTGGAGTTTGCTTGCACTCGCTTTAAAGCTGAATACCCCATATTTGATAA
GGTATATATCCTTTTGACTTTATATGAATGAAGTTGCTAATTGTACTTTATATAGTTAATGT
TTCTTAGCTTAATAACTAGTCTTTTTGGGTCTGCAAAACTCCTTATTCCGGTTGCCTATG
TGATAATATGGTGTGCAAGCCTTTGTAAAGGCTTCAATGACATTAAGATAACGAGCTCT
TCTCCTCATGTCCACATGGTTTTAGGCATGTTGGAGTTGACTTCATTTCAATGGATTGCA
GGTGGATGTGAATGGCGAGAACGCAGCTCCGATATACAAATTCCTGAAGGCTAGCAAA
GGTGGAGCTCTGGGTGGACTTCTGGGGGACGACATCAAGTGGAATTTTGCCAAGTTCC
TGGTGGATAAAGACGGACATGTTGTGGATCGTTATGCCCCCACTACTTCCCCTATTAGCA

TCGAGGTAAACCACCTCCATTTTTGCCTTTTCTTTTGGTTCATTTATACCCATCACACCA
TTCAATGATCAAAGCTCCTAATTGATTTCAGTTGATATTTGCAGAAGGATATAAAGAAA
CTGCTTGGCTGA

GhGPX6	E1	I1	E2	I2	E3	I3	E4	I4	E5	I5	E6
bp	48	121	77	143	62	145	119	241	180	99	27

>GhGPX7 CotAD_39878 (1872 bp)

ATGACGACCCTAACTCCAAATAACCCAGACTCCGTCTATGCTTTTACTGTTAAGGTTGG
TCTGTCGTTCTTTGCTCTAATCCTTTTTTTCTTATCACTGTTTAAGCTCTTCAGTAAGTTT
TAAGTCCACTTAAGAGCTTTAAGTTCACTTACTTACCCTTTTTAAAGTAAAATCTTGCTA
GGCTATGTTGTTTTGTTCTTTAAACTCATCATATCTTGTTATAAAATAGCAAATTTGGTAA
ATTTTGATGGTTGCTTGACACCTTTTCAGTATTTTTTGGGTTCTAAACTTATCACTAGTA
AGACTTCTGTTTGCGGATTACCCATTTTGCTAAGGTTGTTTAGTTACTTACTATTGACGG
GGCAATAGAAAGGAAACTAAATTAACTTGATACGGAGATGCTGACCTGAGATATCAGT
GGAGAAATGATCCTTTTTTTTTTTGTATGAAAATAACGCCAAATGCAGAAACTTTCTCA
GTTTCTTAATGCTTAACAAGGGTTCTTAATGGTTTTTAATCTAATGAGATTTTATTGAGCA
TCAGATGGAGGAGAAGAAGGTAGTGAAATTAAATTGTAATATTCTATTTCATTGCTAAC
CAATCAAATGGTTGTAAATGGTATTGTCCTGGTTTCTTTTACAGAAAAGGATTTAAAAG
GAGCATTTTGTGTATATATTTTCTCTTTTATGCATATGATCCTCTGCTTCTGTTGATAGGAT
GCTGAGGGAAACGATGTGGATCTCAATATTTACCAAGGAAAAGTGATGTTGATTGTTAA
TGTTGCTTCCAAATGGTATCTTCTTTCTATTTCTGTGATCAATATTTAATGTGTTATATTTG
CAACTGAGCCAATTGACGACAGTTTGTAGCCATGAAATTATTTTTATGACTATCTCTTCG
GTTATTTTATGACAGTGGAATGACAAATTCCAACTACACAGAACTGAACCAATTATACG
AGAAGTATAAAGATCAAGGTTTGCATTTTGAACCTTTGAGTTTGCCTGAACCTTCATTA
TCGATGTGTTTAGAAGAACAATGCAATACAACAGCCTCTACTGTTTCCTTATGTGTGCAT
ATATTTGTTGAACTTTTATCGGGATGAATTCCACTTTGAAAGATACATTGACATTACATT
GCTTGCATGGACTAAAATCTGCTTTTGTTTCTGTAAAAGGCTTGGAGATTCTTGCTTTTC
CGTGCAATCAATTTGGAGAGGAGGAACCAGGATCAAATGTTGAGATTTCTGAATTTGT
TTGCACCTGCTTTAGATCGGAATTCCCTATCTTTGATAAGGTAACTAGAACCATACTGTA
CTTGTTTTACTGTTGAAGCTTAGTAAAACTTGGGTAATTTTGCCACAATCATTGTTAGCA
TTTCTGAACTTTACAGTTTTTTTAACCAGGATTCCTATAGTTGAGGATCTACCCCGATGG
GCATACTTTAGGCACAATTTTGCTAACATGATTGCATTAAGAACACCGCTCATGAACCG
TATGAATATAATGTGTAACTTTTTATTGAAGCATCTCGTCTTGCTGTTGTGATATTAGATT
GAAGTAAATGGTGATAATGCCTCTCCACTGTACAAGTACTTAAAGTTGGGGAAATGGG
GAATTTTCGGAGATGATATCCAATGGAACTTCGCCAAGTTCTTGGTCAGTAAGGATGGT
CAAGTTGTTCATCGTTATTACCCCACCACTTCTCCACTTAGTCTTGAGGTGAACTACTTT
TTTTTTTGCCTTCCCAATTCTTATGCTTAAAAGTTTGATTTTGGCATTTTATATTGCGGTG
TTGTTATTATGAAGTGAATTTGATCTTTTGAATTATATGGTGTTATTGCAGTATGATATAA
AGAAGTTACTGGAATAA

GhGPX7	E1	I1	E2	I2	E3	I3	E4	I4	E5	I5	E6
bp	54	662	77	122	62	200	119	257	168	124	27

>GhGPX8 CotAD_59095 (1491 bp)

ATGGCTTCTCAATCTTCTAAGGGATCAGTTCATGATTTCACTGTTAAGGTTGGTTTTGGG
TTTAATTCAAATTTATACGCCCATTGATTAAATTTAATTTGTCTTTCATTTATCTCACTGGG
TTTTGTTTTTGTATTTTGGATGTTCAGGATGCAAGAGGGAATGATGTTGATTTAAGTATT
TACAAGGGCAAGGTTTTGTTGATTGTCAATGTTGCATCACAATGGTAAATTTCAATTTT
GGAGGGTTTTTTCCCCCTATTGTCCATTATTAATCATTTTTTAATTAGTAGTATCTAGACAT
AGTTTTTACTTTTGCTGCTTAAATGTTGATGCAGTGGCTTGACCAATTCCAACTACACTG
AGCTAAGTAAATTGTATGAGCAATATAAAGATCAAGGTTTGTTTTTGGCATCTTTTTGGC
TTTATACATATATATGTGTATTATTTTTTTATATATAATATATTCCAAGAGGTTCTTGTGGTT
GGAAAAAGTGGAAATGAAATTTTCAGGCATTTAGACCAAGTTGTCAGGAGGGCTTTAC
TTGAATACCATCCTGACCAAACAGGATTAGGTTCAGACTTAGTCCCAGATCGTAAAACG
ATGAGGACACTTGATTAATTGAGTTGTTACTATTTGTCCTAACTTTATATTATATTACTTGT
TACTGTCTTGTGCAGGTTTTGAGATTCTTGCATTCCCATGTAACCAGTTTGGAGGACAG
GAGCCAGGGAACAATGAGCAAATCTTAGAGTTTGCTTGCACTCGCTTTAAAGCTGAAT
ACCCCATATTTGATAAGGTATATACCTTCTAGCCATATGAATGGAAGCACTGCTTGTAGT
GTAGGTTAATATCTTTGGAAGCTTTGACTCTAGCTTACACTGAGAGGGTTGAAAATCAA
ATATCTTAAACAGACTCCTCTGTTGAACTAGGAATAGGTGAATTACTGTGTAGAGTTATC
TCAAAATCAAGCCCTTTATAATGCTGTACTAAGATTATGAAATTATTAGTATTATCAATTG
AGGAAGTTGTATGATGTCATGTGTTCTGGTTTACTCATGGGATTGTTGCTTCTACAGTGT
TATAGAACCGACCTTAATGTCGTTTAATTTGATTACAGGTTGATGTGAATGGTGAGAAG
GCTGCTCCCGTATACAAATTCCTCAAGTCTAGTAAAGGTGGACTTTTTGGGGACAGCAT
CAAGTGGAATTTTTCCAAGTTCTTGGTCGATAAGGAGGGCCATGTCTTCGATCGTTATG
CTCCCACTACTTCTCCTCTTAGCATTGAGGTAAGTCCTGTTAATTTCATGTATTTTGCTAG
TGATGTTGTAACAGGAGAAAGTGGAAATGAGAACATCCCAAGGGAAATTAAACCCTG
GAATTGACATAAGTTCTGTATTTATAGACTGAATATCTTCACATATTGGGTTATATAACAT
CCAATGGTTGTTATTTCTATTTGCAGAAGGATATTAAGAAACTGCTGGCTTGA

GhGPX8	E1	I1	E2	I2	E3	I3	E4	I4	E5	I5	E6
bp	48	101	77	111	62	281	119	321	168	176	27

>GhGPX9 Gh_D12G2260 (1451 bp)

ATGCTCGTTCGACGAAATCTAAGTGCACTTGCTGTGTCTGCTTCTCTACTTTTAGG
AAAGCGTTCCTCACCGAGTTTCAACCAAACCCTTTTGAGTTTTCCACAGATTTCAC
CAGTTTATCTGGTATCTCACTCGATCAAAACAGGGTCTCCAAGGTCTCTCGTGGG
TAGTTTGAGATTTGATCATACTATGGCTGCTGAGTCCTCCAAGGGGTCAGTTCAT
GACTTCACTGTTAAGGTTGGTTTTGGGTATAAATTTTACCCCGACAAACGAAGTTT
ATTTGATTCAAATTTAGTTTTGTCTTTGATTCAATTTTCAATGTCCCTCCCCTCCTT
AAGATATGGGTTTGGTTCTTCAGGATGCAAAAGGAAATGACGTCGATTTAAGCAT
TTACAAGGGCAAGGTCCTCTTGATTGTCAATGTTGCATCTCAATGGTACCTCTAAT
TTCAATTTTGTGTAACAAGGATATAGTGGATTGATTGCACATTTAGATTATGCTTT
TCTTTGGCCTACTTGTTGGATTTTTAGCTTTAATTTCTGGACAGTTTTGAATTTGGT
GCTTAAAATGTTGATGCAGTGGCTTGACCAACTCCAACTACACTGAGCTGAGTAA
ATTGTATGAGAAATATAAAGATCAAGGTTTGTTTTAGCATCCTTGTAGCTTCTTGT
TTCTGAGGACCATTCATGAGCCTTTAGTTGATTTCAGTGAAGTGAGGTGTAGACA

GCACTTGAAGTATCATGGAACTGCCATTTGATTATTTGATTTGCTGGTTTGTCGGG
TCAGGTTTCGAGATTCTGGCATTTCCATGTAACCAGTTTGGGGGTCAGGAGCCAG
GGAACAATGAAGAAATCTTGGAGTTTGCTTGCACTCGCTTTAAAGCTGAATACCC
CATATTTGATAAGGTATATATCCTTTTGACTTTATATGAATGAAGTTGCTAATTGT
ACTTTATATAGTTAATGTTTCTTAGCTTAATAACTAGTCTTTTTGGGTCTGCAAAA
CTCCTTATTCCGGTTGCCTATGTGATAATATGGTGTGCAAGCCTTTGTAAAGGCTT
CAATGACATTAAGATAACGAGCTCTTCTCCTCATGTCCACATGGTTTTAGGCATGT
TGGAGTTGACTTCATTTCAATGGATTGCAGGTGGATGTGAATGGCGAGAACGCAG
CTCCGATATACAAATTCCTGAAGGCTAGCAAAGGTGGAGCTCTGGGTGGACTTCT
GGGGGACGACATCAAGTGGAATTTTGCCAAGTTCCTGGTGGATAAAGACGGACA
TGTTGTGGATCGTTATGCCCCCACTACTTCCCCTATTAGCATCGAGGTAAACCACC
TCCATTTTTGCCTTTTCTTTTGGTTCATTTATACCCATCACACCATTCAATGATCAA
AGCTCCTAATTGATTTCAGTTGATATTTGCAGAAGGATATAAAGAAACTGCTTGG
CTGA

GhGPX9	E1	I1	E2	I2	E3	I3	E4	I4	E5	I5	E6
bp	237	121	77	143	62	145	119	241	180	99	27

>GhGPX10 Gh_A12G2084 (1453 bp)

ATGCTCGTTCGACGAAATCTAAGTGCACTTGCTGTGTCTGCTTCTCTACTTTTAGGAA
AGCGTTCCTCACCGAGTTTCAACCAAACCCTTTTGAGTTTTCCACAGATTTCACGAGT
TTATCTGGTATCTCACTCGATCAAAACAGGGTCTCCAAGGTCTCTCGTGGGTAGTTTG
AGATTTGATCATACTATGGCTGCTCAGTCCTCCAAGGGGTCAGTTCATGACTTCACTG
TTAAGGTTGGTTTTGGGTATAAATTTTACCCCGACAAACGAAGTTTATTTGATTCAAA
TTTAGTTTTGTCTTTGATTCAATTTTCAATGTCCCTCCCCTCCTTAAGATATGGGTTTGG
TTCTTCAGGATGCAAAAGGAAATGACGTCGATTTAAGCATTTACAAGGGCAAGGTCC
TCTTGATTGTCAATGTTGCATCACAATGGTACCTCTAAATATCAATTTTGTGTAACAA
GGATATAGTGGATTGATTGCACATTTAGATGATACTTTTCTTTGGCCTACTTGTTGGAT
TTTTAGCTTTAATTTCTGGACTGTTTTGAATTTGGTGCTTAAAATGTTGATGCAGTGGC
TTGACCAACTCCAACTACACTGAGCTGAGTAAATTGTACGAGAAATATAAAGATCAA
GGTTTGTTTTAGCATCCTTGTAGCTTCTTGTTTCTGAGGACCATTCATAAGCCTTTAGT
TGTTTTCAGTGAAGTTGAGGTGTAGACAGCACTTGAAGTATCATGGAACTGCCATTTG
ATTATTTGATTTGCTGGTGTGTCGGGTCAGGTTTCGAGATTCTGGCATTTCCATGTAAC
CAGTTTGGGGGTCAGGAGCCAGGGAACAATGAAGAAATCTTGGAGTTTGCTTGCACT
CGCTTTAAAGCTGAATACCCCATATTTGATAAGGTATATATCCTTTTGACTTTATATGA
TTGATGTTGCTAATTGTACTTTATATAGTTAATGTTTCTTAGCTTAATAACTAGTCTTTT
TGGGTCTGCAAAACTCCTTATTCCGGTTGCCTACGTGATAATATGGTGTGCAAGCCTT
TGTAAAGGCTTCAATGACATTAAGATAACGAGCTCTTTTCCTCATGTCCACATGGTTT
TAGGCATGTTGGAGTTGACTTCATTTCAATGGATTGCAGGTGGATGTGAATGGCGAGA
ACGCAGCTCCGATATACAAATTCCTAAAGGCTAGCAAAGGTGGAGCTTTGGGTGGAC
TTCTGGGGGACGACATCAAGTGGAATTTTGCCAAGTTCCTGGTGGATAAAGAGGGAC
ATGTTGTGGATCGTTATGCCCCCACTACTTCCCCTATTAGCATCGAGGTAACACACCT
CCATTTTTGCCTTTTCTTTTGCTTCATTTATACCCATCACACCATTCAATGATCAAAGC
TCCTAATCGATTTCAGTTGATATTTGCAGAAGGATATAAAGAAACTGCTTGGCTGA

GhGPX10	E1	I1	E2	I2	E3	I3	E4	I4	E5	I5	E6
bp	237	121	77	144	62	146	119	241	180	99	27

>GhGPX11 CotAD_39520 (1862 bp)

ATGACGACCCTAGCTCCAAATAACCCAGACTCCATCTATGCTTTTACTGTTAAGGTTGG
TCTGTCGTTCTTTTCTCTAATCCTTTTTTTTATCACTGTTTAAGCTCTTCAGTAAGTTTTA
AGTCCACTTAAGAGCTTTAAGTTCACTTACTTACCCTTTTTAAAGTAAAATCTTGCTAGG
CTATGTTGTTTTGTTCTTTAAACTCATCATATCTTGTTATAAAATAGCAAATTTGGTAAGA
TGGTTGCTTGACACCTTTTCAGTATTTTTTGGGTTCTAAACTTATCACTAGTAAGACTTC
TGTTTGCAGATTACTCATTTTGCTAAGGTTGTTTAGTTAATTACTTTTGATGGGGCAATA
GAAAGGAAACTAAATTAACTTGATACAGAGATGCTGACCTGAGATATCAGTGGAGAAA
TGATCTTTTTTTTGTATGAAAATAACGCCAAATGCAGAAACTTTCTTAGTTTCTTAATGC
TTAACAAGGGTTCTTAATGGTTTTTAATCTGATGAGATTTTATTGAGCATCAGATGGAGG
AGAAGAAGGTAGTGAAATTAAATTGTAATATTCTATTTCATTGCTAACCAATCAAATGGT
TGTAAATGGTATTGTCCTGGTTTCTTTTACAGAAAAGGATTTAAAAGGGGCATTTTGTGT
ATATATTTTCTCTTTTATGCATATGATCCTCTGCTTCTGTTGATAGGATGCTAAGGGAAAC
GATGTGGATCTCAATATTTACCGAGGAAAAGTGATGTTGATTGTTAATGTTGCTTCCAAA
TGGTACCTTCTTTCTGTTTCTGTGATCAATATTTAATGTGTTATATTTGCAACTGAGCCAA
TTGACGACTGTTTATTGCCATGAATTTATTTTTATGACTATCTCTTCGGTTATTTTATGAC
AGTGGAATGACAAATTCCAACTACACAGAACTGAACCAATTATACGAGAAGTATAAAG
ATCAAGGTTTGCATTTTGAACCTTTGAGTTTGCCTTAACCTTCATTATCGATGTGTTTAG
AAGAACAATGTAATACAACAGACTCTACTGTTTCCTTATGTGTGCATATATTTGTTGAAC
TTTTATCGGGATGACTTCCACTTTGAAAGATACATTGACATTACATTGCTCGCATGAACT
AAAATCTGCTTTTGTTTCTGTAAAAGGCTTGGAGATTCTGGCTTTTCCGTGCAATCAAT
TTGGGGAGGAGGAACCAGGATCAAATGTTGAGATTTCTGAATTTGTTTGCACCCGCTTT
AGATCGGAATTTCCTATCTTTGATAAGGTAACTAGAACCATGCTGTACTTGTTTTACTGT
TGAAGCTTAGTAAAACTTGGGTAATTTTGCCACAATCATTGTTCGCAGTTCTGAACTTT
ACAGGTTTTTTAACCAGGATTCATATAGTTGAGGATCTACCACGATGGGCATACTTTAG
GCACAATTTTGCTAACATGATTGCATTAAGAACACCGCTCATGAACCGTATGAATATAAT
GTGTAAATTTTTATTGAAGCATCTCTTCTTGCTGTTGTGATATTAGATTGAAGTAAATGG
TGATAATGCCTCTCCACTGTACAAGTACTTAAAGTTGGGGAAATGGGGAATTTTCAGAG
ATGATATCCAATGGAACTTCGCCAAGTTCTTGGTCAGTAAGGATGGCCAAGTTGTTCAT
CGTTATTACCCCACCACTTCTCCACTTAGTCTTGAGGTGAACCACCTTTTCTTCTGCCTT
CCCAATTCTTATGCTTAAAAGTTTGATTTTGGCATTTTATATTGAGGTGGTGTTATTATGA
AGTGAATTTGATCTTTGGATTTATATGGTGTTATTGCAGTATGATATAAAGAAGTTACTGG
AATAA

GhGPX11	E1	I1	E2	I2	E3	I3	E4	I4	E5	I5	E6
bp	54	651	77	122	62	200	119	258	168	124	27

>GhGPX12 CotAD_51884 (1469 bp)

ATGGCTTCTCAACCTTCTAAGGGATCAGTTCATGATTTCACTGTTAAGGTTGGTTTTGG

GTTTAATTCAAATTTATACCCCCATAGGTTAAATTTAATTTGTCTTTCATTTATCTCACTG
GGTTTTGTTTTTGTATTTTGGCTGTTCAGGATGCAAGAGGGAATGATGTTGATTTAAGTA
TTTACAAGGGCAAGGTTTTGTTGATTGTCAATGTTGCATCACAATGGTAAATTTCAATTT
TGGAGGGTTTTTCCCCCTATTATCCATTATTAGTCATTTTTTAATTAGTAGTATCTAGAC
ACAGTTTTTAATCTTGCTGCTTAAATGTTGATGCAGTGGCTTGACCAATTCCAACTACA
CTGAGCTGAGTAAATTGTATGAGCAATATAAAGATCAAGGTTTGTTTTTGGCATCTTTTT
GGATTTATATATATATATAATATTCCAAGAGGTTCTTGTGGTTGGAAAAAGTGGAAATGA
AATTTTCAGGCATTTAGACCAAGTTGTCAGGAGGGCTTTACTTGAATAACATCCTGACC
AAACAGGATTAGGTTCAGACTTAGTCCCAGACCGTAAAACGATGAGGACACTTGATTA
ATTGATTTGTTACTTTTTGTCCTAACTTTATATTATATTACTTGTTACTGTCTTGTGCAGGT
TTTGAGATTCTTGCATTCCCATGTAACCAGTTTGGAGGACAGGAGCCAGGGAACAATG
AGCAAATCTTAGAGTTTGCTTGCACTCGCTTTAAAGCTGAATACCCCATATTTGATAAG
GTATATACCTTCTAGCCATATGAATGGAAGCACTGCTTGTAGTGTAGGTTAATATCTTTG
GAAGCTTTGACTCTAGCTTACACTGAGAGTGTTGAAAATCAAAGATCTTAAAACAGAC
TCCTCTGTTGAACTAGGAATAGGTGAATTACCGTGTAGAGTTATCTCAAAATCAAGCCC
TTTATAATGCCTTAGTAAGATTATAAAATTATTAGTATTATCAATTAAGGAAGTTGTATGAT
ATCATGTGTTCTGGTTTACTCATGGGATTGTTAATTCTTCAGTGTTATAGAACCGACCTTT
ATGTCGTTTAATTTGATTACAGGTCGATGTGAATGGTGAGAAGGCTGCTCCCATATACA
AATTCCTCAAGTCTAGTAAAGGTGGACTTTTTGGGGACAGCATCAAGTGGAATTTTTCC
AAGTTCTTGGTCGATAAGGAGGGCCATGTCGTCGATCGTTATGCTCCCACTACTTCTCC
TCTTAGCATTGAGGTAAGTCCTGTTAATTTCATGTATTTTGCTACTGATATTGTAACAAG
AGAAAGTGGAAATGAGAACATTCCAAGGGAAATTAAACCCTGGAATTGACGTAAGTTC
TGCATTTATAGACTGAATGTCTTAACATATTGGGTTATATAACATCCAATGGTTGTTATTT
CTATTTGCAGAAGGATATAAAGAAACTGCTGGCTTGA

GhGPX12	E1	I1	E2	I2	E3	I3	E4	I4	E5	I5	E6
bp	48	101	77	111	62	258	119	322	168	176	27

>GhGPX13 CotAD_36707 (1400 bp)

ATGGGTGCTTCTGAATCAGTTCCTCAAAAATCAATCCATGAATTTACAGTCAAGGTTGG
TTTTCTTTCTTTCTGTTCTTTTGCTTTAATGTTAAGATTTTAAGTGAATGATTTTGATGTT
TGAAGGTTTTTTACTATGTTATATATTTGTGTTTGTAGGATTACAAAAACCAAGATGTTG
ACCTTAGTATGTACAAAGGAAAAGCTCTTCTTGTTGTTAATGTTGCCTCTAAATGGTTCT
TTTTCTCTTTTTTTCTAATTTTTCTTCTTCTTTTTTTAATGTAATTTTTTTAAATTTATAAAG
ATTACACCTTTGTATTTTTGAACAGTGGACTCACTGATTCAAATTATACCCAGTTGACTG
ATCTTTACAACAAATACAAGGACCAAGGTTAGTTCCTTTTATTTAGATTTTTGTAATTATT
AGTTTCTAGGGTATTTTGTGTTATATGAACAGGTAGATGATTTTTTTTACAATTTTTGTTC
CTGGATTTGTTGAAGACGGTTGTGTTTTTTTTTGTGTTTGTGATGGGAAGGACTGGGGA
TTTTGGCATTCCCATGCAATCAGTTTTTGAGCCAAGAACCTGGTACCAGTCAAGATGCA
CAAGAATTTGCTTGTACAAGATACAAGGCTGAATACCCTATTTTCCAAAAGGTAATGAA
AATATGTATGTATGTGTGTGTATCTTTCGTTCTAGCTGCATATTTTGTTTTGTTGATTTGA
ACCTAAATTAATCCCAACTTAAATTGACTGAATCTGAACATAAAACAATCCCAACAAGT
AACTTGAATTGATCCGATCCAAAAGTATAAACTCGAAATTGACCCAAACTTAAAATGAC
CATAAAATTTTACAACTTAAGACCAACCCAATCCACTAATTGACTAGGCTAACTTGAAT

ACATATGACCAGCTCATTTTTTATGAATGAATGAATGAAAGCTTTGACTGTTTATCATGT
TTGCTGCTTTCTTCAATACAGGGACATGTTGGGGACTTTGCATTTGCTAACTCATTGCTC
ATTGCTGCTAAATTATTTGTTCTATGTTTAGGTTCATGTCAATGGTCCAAAGACAGAGCC
TGTCTACAAGTTCTTAAAGACAAACAAATCTGGGTTCTTGGGAAATAGGATAAAGTGG
AACTTCACTAAGTTTTTAGTTGACAAGGATGGCCATGTCCTTGCTCGCTATGGCCCTAG
CACTACGCCATTGGCCATTGAGGTACTCCCTCCCTCCCTCCCTCCTATCTTTGTGTATCT
GCAATCTTAGGGAGTAGCAATTGTTCGTTATTTAAAAAAATTGACTTTTGCTATACCCTA
TATATGATAATGTAGACTAATTATCTGCTGTGTTGCTTTGCTTACAGGCTGACATCAAGA
AGACTTTGGGAGTGGACATGTGA

GhGPX13	E1	I1	E2	I2	E3	I3	E4	I4	E5	I5	E6
bp	54	104	77	93	62	145	119	397	168	145	36

Supplementary data 4: putative *cis*-acting regulatory elements related to stress and hormone response in promoters of *GhGPXs*.

>CotAD_10469 GhGPX1

GTAGTGGAAGGTAAAAGTCCACGTTGGTTTTCTTTTAACTACTTTTAATTTTGTTTGTAA
GCAAGTAAACTTATTTTTGGGATACGTCAAGGCCCATTTAAAACCTTGAAAAACTTCAT
GGTTGGGTTGGTTCCTGAAGTTTCTTACTGTTGTTGATGATGACTGGCTGATAATTGAGT
AGCGTGGCGCCTGGTGGGTCAATAGCTTGTACTATTTCTAGGACATATCCAGGTTGGAG
AGTTTGAGCCTTAGAGGGAAAGAAAATGCATCTGATCCTCAATAAATTTTGATACAAGT
TGGCAATTCCAAAACTAGGCTAAGCGTGGAGAAAAGTTATGGGCCTTAAATTTACAATC
TCATCATCAACTGGGGTTTCAAAATGTCCATTCAAAAACCCTAGCTTTTTTTTTTCTCTA
TTATCTAGACATGCAGTTTGTCTTTGTTCTATGTTCGAGTGTCTTCCAGAAAGGGATGAT
TTTTCAGATATGGGGTTCTAAAAAAATTGAAACTTGTTTTGTTTGTTATCTATCTTTCTTT
GCCTTGTTTTTTAGTTGTCCTAATTCTTGGCAGAATACTTAAGCTTATGTAGCTGCTGTA
AATATGGACAAATATCAATATGCAGGCTTTAAAGTGGGCGAGCCATTAGTACATGGACT
CCCATAAAGAACCTACATTACATTACAGGTTTCTATAAAACAATAGGTTTGCATGAGTTC
TGATTTATGTTCATTATAGCAAGTTGAGTTTTTCTTTTTATCTTTTCTATGATGAACAAAC
TATAATGCTTCTAACACTACTTTTTTTATTTTTCGATCCCCTTTGGGTCCATTGTTTGATC
CTCTTTTTGTGTTTTCTTATAAATTTAATGTTTCTGTTTCTGTTTGTAATAGGCTGGGGGG
TTTTTTGGTTTGAAGTTCATGAGCATGTGTGGTATGAATACGAACAACTGTTGAACTTAT
GCAAGTAGTTTTTCTCTTCTTTAGATGATGATCCAGAGGAATCATGTTTGTGAACTTGA
CATGTGAAACTATGGACAGATTTTTAGCTTATTTTGCATTTATATCTTGTTAAGGTTATAG
ATAGTGAGTTCCAATTCCCAATAGATTTGGTTAATCTTGTATTTTGATATGATTTGCCCAT
TTTTGAACATTCATTTGCCATCTGATATTTCTTCAATGAAAGGCATCTTAGACATTGCTT
CTTGGTTGTTCTAATTAATTGTCAACTAGTAATACAGATTTTGGAATATTGTGGTTCTTCT
GTAAGAATGAATGAATGAATGGATTATGGTTGGATATATTTCTGAAACTATTATATTTCAA
CTTTTGTCAACTCCTACTTCAGTTTCGCTCAGTTGTTGATTAGTTCAGAGGTAGTTGGTT
AGATAGTCATGACTCCTTCCTCAAGTTACGAAGATGGAAAAGGGCATTCAACCACGGG
CTAAGATGCTGGTGCAATTTATGATGCATGTTACGAATTCAAGGTGGAGCAGCTGCCTG
ATTCACAAGTCTTTCCTTCATTCCAGTCTTGGACAAACTCCTATGATTCCTTTCCCAATG
TCTATATTCTCCTAATAGAATCAATTGGTTTATCTTTATATTAATAGATGGTTAGAAATTAC
AGCTTATGTTTGTTCATCACTTTATCTTTAGCTCCAAGCTCAACTCCTAACTCATTGTTCT
GAGATAACAGGGTCAATCTGCTGCCCTTGGAGATAAAAGATCCTGGAATACAATTCATG
ACTGGACTAAAATGGTTTCGAATAATAGTGATTCAAGGCTGTAGGATAAGAAGCAATGA
AGCAAAGGATCAAATTTTATCTAGCCTAGGGGTAACGTCTTACCATTTATTCCACTCCCC
ACCCTCTTTTTTCCTCAGTATTTTGGTTATTTGCTTTATAATTTATATCACTTATTCTTTATC
TCTAAGTCATTTAGAAAGATTCCTTATCTGGAATATCTCATTTGATTCTCCATCTGCAAG
GTTTTATTGTGCGTCTT

ARE: TGGTTT (+) Anaerobic induction

AuxRR-core: GGTCCAT (+) Auxin

CGTCA-motif: CGTCA (+) MeJA-responsiveness

GARE-motif: AAACAGA (－) Gibberellin-responsive element

MBS: CAACTG (+, －) Drought-inducibility

TCA-element: CCATCTTTTT/CAGAAAAGGA (+, －) Salicylic acid

TGACG-motif: TGACG (－) MeJA-responsiveness

>CotAD_76066 GhGPX2

CACGGTTTGAGATGGAAAGAAAAAGGTTTTTGAAGCTTTTGGATATTTAAATAAAGGG
AAGGAGGAGATGGGGTAGTAAAGGTGGGTTCTGGCAGTGAAAAGGATAGCTATTTTTG
GTAGTGATGACAAAATAAAAATGGTCGAGTTTGACAAGTAAAGTTAATTGTGGGTTTTT
GCAGGCATTAGTTATAACTATTGAGGTTGATGATGATGCAAATTGAATTTTGGGAAAGG
TGTGTTCACATTCATATAATCACGCAGAGTTTTCTTTGCCAAAGTCAAATTTAAAATCAA
TCACCAAATAATTTAAAGCATTTATAAGATAATTGAAAAACGCGTTTAAGCATCTAAAA
AGTCTTAAAACTTGGTATTAGTAGGACTGATAGTACGTAAATAAAGTATAATTAAATGTT
GAAAAACTGGAGTGCCGACTTTTTATGAAGCAGCCAGCCAGAAAGCAAATCCTCATTT
AATGATATTTAAAACTCACAACCCCACGCCAGCTCTTTATCCCCCCATTTCCACGGTTG
GATTATTCATATTTTTTTGTTTCCCCCAAATATTAACCTTTCACATCTCTCTCTCCGTTAAA
TTTTTAGATCTATGTAAGGGTGTAAATTAACTGTTTGTGAATCGTTTATATAACGTTTGCT
TATATTCGTTTATTTAGTTAAATAAGTGAGCATGAACAAAATTTTTATGTTCGTTTAATAA
ACGAACGAACATGAACATAGGTGTGTTCGGTTCATTTATGTTCACGAACAAACTCGTTT
AAAAGCTCATTTATTGGCTATTTGTGTCTTACTTTCCATAACTTCTTTATAGAACTTTATC
TTGATCTGATTATTGTGAACATCTTTCGGATTCAAATGGGCAAAAATGTACTTCTTCCAA
TGGAAAACAAAGAAATATCAATTTAATTTACCATGATGAATAACATCACGATTATACTGA
TATGGCCTACCAAGTAACAAGTGGGCGACATGCATGAGAATGACATCACACCAAACTT
CATCAATGTAAGTCCCAAGTTCAAAAGGGATCAAGCATTGTTTGACAACACGGACTTC
TGAACCTTCGTTAAGCCATTGAAGATGGTATGGTGTAAGGTGTTTGCTACATGGCTGTA
ACACTCCTAACCCATATCCGTCGTCAGATTAGGGTTACTAGGTATGAACAGATATAGCAC
AATTGAGTATAATTAAAACAAAGTCATAATTCACAAAGTGATTTAAAGAAATAAATATAT
TTTACTTCATAAAATACATCAACCGAAAATGCAAAGTCAAACATCATTTTAATCTCAAA
ATATTATAGACACAAAACCAATTTTTTTTAATCAAATTTATTTATAAATACCAAAACCGTA
TGTACATGTTCAAAATTATTTATACAAAAACTATTTACAAAAAGTTTTTCATTTTATTACC
AGATAACAATGCATTTCATAGCACATTTTAAACATATATATAATTGAACATTCATTTAATTT
ACTTAGAATATGTTAGTAAAATAATTCCAAAATTTCGCATAAATACTTCCATTTATAAGTA
TACTATATATATACCTATATATATAATCATATAGAACTAACTCAAACATACCACATAACCAT
GCATCAATATACCAAGTCTCATATAATACCAAACCACAACCACAATATGAGTAATTTATC
AATCAAACCAAAACCAACTAAAACAACTAATTTAGCCACATTACATGGCTTTAATATAG
ATATATTAACAAAATAACAAATTCAACTAGTCTTAGACTATACATGCCATAGACTCAAAA
GGAACTTCAACTTAAAATACCATTATCTATCGATAGTGTGATAAGCTTTTCTGACGATCC
CCGATCTCGTATCAACTTCAAAAATCTATAAAACAAAGAAAATAAACAGAAACAAACA
TAAACAGTAAGCCATTGATAGCTCAATAAGTTATTAGCAATCAAATTAAAGAATCATAA
AATTTTATAAGCCAATTTATTTTAAACAC

ARE: TGGTTT (－) Anaerobic induction
CGTCA-motif: CGTCA (+, －) MeJA-responsiveness
GARE-motif: AAACAGA (+) Gibberellin-responsive element
LTR: CCGAAA (+, －) Low-temperature responsiveness
MBS: TAACTG (+) Drought-inducibility
P-box: GACCAAACTCGT/GCCTTTTGAGT/CCTTTTG (+, －) Gibberellin-responsive element
TGACG-motif: TGACG (+, －) MeJA-responsiveness

>CotAD_22672 GhGPX3
GTTATCAATATGAAGCGGAGAAGATGGTGATGGGTATGTGTGAAATTGAAAATACAGA
AACAACGCAAATCCCAGAAATAGCAGAGAAACCAAATTGCTAAATTTCCAGTCCCAAT
ACATTTTTTTTTAATTTACTATAGTTTCCTGCCTTCCACAATGCACTGCTGCTACCAAATT
TCAGTGTTTATTAATGACTTGACTCGTAAGATTTGATTCTAGAACAAGAAGTAATTATAA
AAGTTGTTGCCATATATTTTTAAACTACTCACTGCATTATTAATAAATTTAATTTTTATCAC
TTTATTTTAAAAAATTACAAAATAGTCACTAAACAATTTAAAAGTTTTTATTTAAGCCAC
TAGACTATATGAAAGTTTTTATTTAAGATACTACGCTGTTAAGCTTTTTCTTTTTAAAGTT
TCTTGGAGCTCAAATATAAACTTTCGAATAATTCAATGACTTAAATGAAACTTTTAAATA
GTTCAGTGATCATTTTGTAATTTTTTAAAGTTGAGTGCTCAAAACGTAGACTTATTAATA
ATTTAGTGACCTTAAGTGTTGTTTACCTTATTTTTATTTATCTATTATTTTTTTTAAAATAAT
AGTTTTTTTTCAAATATTTCTAACTCTTCTCAATTTAATATTAAGTAAATTAGTTATTTGG
AAAAAATAAAACAAGTTAATCCTATTAATTTAAAAAATAAGTAATTAAAAACAATTAATA
ATATCGTCAATATTTTTCATTAATTTATCCTATTTAATTTCTCATTTTTAAAATAAACACATA
TTAAATTGTTTTATTTTGTTTTATTCTTTTGAGAGGGATTAATTTTCTAAATATTAAAACTA
AAATAAACCAAATAGTTCTTTTTATAATTTTTAATCCAGTAAATATTTTTTTAGGATAAAT
ATTAATATTTTTTGGGTTTTTTTTCAATAGTCCTTTGTGTTCATATTATTGATGAAAAGTTT
TTTCTATTCATGTTTTATATTTAAATTGTTTTTTAATTACTAATTGATACTATTTAAATAATT
TATAATTTGATTAACCTATATTTTAATTCTTAAATTTGTATTTAATTGGTAATTAATTTTTTT
GACTAAAAACTCTTTAAAGATCTTGAATTTTTTCAAAAAAAATTCTTTTTGTTTTTAGTC
AATTGAGCTCTTAAATGTCAAAATTTTAATCAATTATGTCCTTTTAACTGCTAAAATTTAC
AATTAAAAATAAAAAAGTAATGATAACTGTTTTGGAGGTTGTTTTCGCCACATGGCACA
TCAAGATTGTGTCACATGATAAATTTTGGTATTTTATATTTAAAATCATTAAAATTATTAA
AAATTTACACAAAAATATTGATAATATTAAAAAATTAAAAACTTATAAATATAAATGACA
CAAAAATTGTATATATTATAAAAATTATTAAAAAAAGCAAGAAACATTAAAAATATTAAA
ATAATAAAATATATAAATTAAAAATATAGAAAATTGTTTAAAATATTATAAAAATGTAGGA
AAGTGCAAAATATTTATAAAAATAAAAAATTATAAAAAAATTATTTCAAATTATTCAAAA
AATAATAGAAAATTATAAAAAGTTTCATATAAAATATAAAAAATTTTAAAAAACCTACAA
TTTTCTAACCAACCTCAAGGAGATTCATGTATACGAGAACAATAGATAGACTCAGAGAT
CAAATGATGGTTAGATTTTTTATGATTGTCCCCCTTGATTTTTATAGTTTTTATTTTTAATA
TTTTTTTAGATTTAAATTTATAGTTTTTATTTTTAATATTTTTTAGATTTTTCTTATAAATTTA
TATATTTTAAAATTTTTCATATGTCATTATATTATTGGTTGTCAGTGCCAAACCATTAACCG
AGTTGGTACTTTTTTTAAATACCTAATCAACTTCATCAAAAAATTTTATTATATGGCTCTG
ATAACTAATAATACATTGTCCCATGGCATCCACAAATTATAACATGTGGCAA
ARE: TGGTTT (-) Anaerobic induction
CGTCA-motif: CGTCA (+) MeJA-responsiveness
ERE: ATTTCAAA (+) Ethylene-responsive element
MBS: TAACTG (+) Drought-inducibility
TCA-element: GAGAAGAATA (+) Salicylic acid responsiveness
TGACG-motif TGACG (-) MeJA-responsiveness

>CotAD_54994 GhGPX4
CACGGTTTGAGATGGAAAGAAAAAGGTTTTTGAAGCTTTTAGATAATTAAATAAAGGG
AAGGAGGGGATGGGGTAGTAAAGGTCGTTTCTGGCAGTGATAAGGATAGCTATTTTTG
GTAGTGATGACAAAGTAAAAATGGTTGAGTTTGACAAGTAAAGTTAATTGTGGATTTTT
GCAGGCATTAGTTATAATTATTGAGGTTGATGATGAAGCAAATTGAATTTCGGGAAAGT
TGTGTTTACATTCATATAATCACGCAGGGTTTTCTTTGCCAAAGTCAAATTTAAAATCAA
TCACCAAATAATTTAAAGCATTTATAATACAATCATTGAAAAACGCGTTTAAGCATCTAA
AAAGTTTTAAAACTTGGAATTAAGAGGACTGATAGTACGTAAATAAAGTATAATTAAAT
GTTGAAAAACTGGAGAGCCGACTTTTTATGAAGCAGCCAACCAGAAAGCAAATCCTCA
TTTAATGATATTTAAAACCCACAACCCCACGCCAGCTCTTTATCCCCCCATTTCCGCTGT
TGCATTATTCAATTTTTTTTTTTGTTTCCCCCAAATATTAAGCTTTCACATCTCTCTTTCC
GTTAAATTTTTAGATACATGTAAGGGTGTAAATGAACACAAGATTTGATGAATTATTCGT
AAATTATTTATATAATGTTTATTTATGTTCCTTTATTAAGTTAAATAAGTGAGTATGAATAA
AATTTTTATGCCTGTTTAATAAACGAACGAACATGAATATAGGTGTGTTCGGTTCGTTTA
TGTTCACGAATAAACTCGTTTAAAAGCTCATTTATTGGTTGTTTGTGTCTTACTTCCTAT
AACTTCCTTATAGAACTTCATCATCTTGATTTTATCATTGTGAACATCTTTCAGATTCAAA
GGGGTGAAAATGTACTTCTTTCCATAGAAAACGAAGGAATATCAATTTAATTTACCATG
ACGAATAACATCACGGTCGTACTGCTATGGTCTACCAAGTAACAAGTGGGCGGACATG
CATGGGAACGACATCACACCAAACTTCATCAATGTAAGTCCCAAGTTCAAAAGTGATC
AAGCATTGTTTTAGCACACAGACTTTTGAACCTTCGTTAAGCCATTGAAGATGGTATGG
TGTAAGGTGTTTGCTACATGGCTAAGCAAGTGAATCCATAAGGTAGCTGCTCACAAATT
TAGCACAGCTTCTACTATCAATGATAAATGAACAAAGTTTACATTTAATCAAACACATCG
AGTGGAATATATTACTCCTTTGAAGATCATGATCATCCCTCAATTGGATGTTGAGGGTGC
GCCTCACAACTAGACAACGGGAATGAGTTAGTGGTTCCACCAACTCTTATTCACCATAA
AACTCACCTCCATCATCGACATCATTAATTAATTCGGGCATATCTATATTTTTTTTTATCTG
AATCAGAGGTATACTCACCAATTCTTTTATCAACAAAAGTCTAGTGTTGGCACAATCAC
GACTATAGTGCCCACGCCCTTTGCACTTAAAACATTCAATTTCACGAGCTTTTTGTTGTT
TTGGTGAAGAAGTAGTAAAAGTGGGTTGCTTAAAAGATGTAGAAGAAGGTTTTGTAGG
AGCCTCACTTTTCCACTCATAAGGTTTAGTCTCATCATGTTTTGGCAGCAATTCATTAGT
AGCATAAGGAGGTTTTGAATTCTTGAAAGTAGAATTAGAACTCGTACCTCTATAATATTG
TGATGCACCAAATGGAAGAGATTGACATTGTTGAAATTGTTGCTCAATTTCAATGGCCT
TGTGTACCGCATCCTGAAGATCAATGTAGGTTTGAAGATTAAGAGCATCGACTATCAAA
ATGTTCAAGCCATCTACGAACCGTACCATGGTAGTCTCGTCATCTTCTCATACATTTTTT
TCTTTGCATAAGCATCTCTATCTCCTTAAAGTATCCATCAACAAATCGATTACCTTGAAC
AAGGCGTTGAAGTTGTCGCTTAATATCCCTATAATA

CGTCA-motif: CGTCA (+, －) MeJA-responsiveness
LTR: TTTCGG (－) Low-temperature responsiveness
TGA-element: AACGAC (+, －) Auxin-responsive element
TGACG-motif: TGACG (+, －) MeJA-responsiveness

>CotAD_58608 GhGPX5

TTATAAAAAAAAAAGAATGCACATGATTGAATGTAATGAAGAAAGAAAAAGAGAATTA
GCGAGAGGAATAAAAAGGCGCAGGTAAAACAAAAGAGAAATTTTTAGAGTCTCGTTC
TTTATTTTCAAAAAAGACTTACCACATATTATAATTTGTGGATGTCATGTGACATTGTGTT
ATTAGTTATCAGAGTCATATAATAAATTTTTTTGATGAAGTTGATTAGGTATTTAAAAAAA
TACCAACTTGGTTATTGGTTTGGCACTAACAACAAATAATATAATGGTGCAGGAAAAGT
TTTAAAATATATAAATTTATAACAAAAATCTAAAAAACTATAAAAATCAAGGGGGACAA
TCATAAAAAATTTAACCATCATTTGATCTCTGAAGCTTTCTGTTGTTCTCGTATACACGA
ATCTCCTTGAGGATATATAGAAAATTGTAGGTTTTTTAAATTTTTTATATTTTATTTGATTT
TTTATAATTTTCTATTATTTTCTATTTTTATAAATATTTTGCACTTTCCTATATTTTTATAACA
TTTTAAATAATTTTCTCTATTTTTAAATTTATATATTTTATTATTTTAATATTTTTAGTGTTTC
TTGCATTTTTTAATAATTTTTATAATATATACAATTTTTGTGTCATTTATATTTATAATTTTTT
AATATTATTAATCTTTTGTATAATTTTTTTAATAATTTTAATGATTTTAAATATAAAATACCA
AAATTTCTCACCTGACACAATCCTGATGTGCCATGTGGTGAAAATAACCTCCAAAACA
GTTATCATTACTTTTTTATTTTTGATTGCAAATTTTATCGGTTAAAAAGACATAATAGATT
GAAATTTTGACATTTAAGAGCTCAATTGAGTAAAAATAAGAAGAATTTTTTTTGAAAAA
AATCAAGATCTTTAAAGATTTTTTAGTAAAAAAAGTTAAGTACCAATTAAATACAAATTT
AAGAATTAAAAAATATGTTAATCAAATTATAATTATAAATTATTTAAATAGTATCAACTAG
TAATTAAAAAACAAATTAAATTTAAAACATGAATATAAAAAAACTTTTCATCAATAATAT
GAACAAAAAAACACCCAAAAAATATTAATAATTTATCCTAAAAAATATTTACTGGATTA
AAAATGATAAAAAGAACTATTTGGTTTATTTTAGTTTTGCTATTTAGAAAATTAATCCCT
CTCGAAAGAATAAAACAAAATAAAACAATTTAATCTCTATTTATTTTAAAAATGAGAAA
TTAAATAGGATAAATTAATGAAAAATATTGACGATATTATTAATTATTTTTAGTTGCTCATT
TTTTAAATTAATAGGACTAAATTATTTTATTTTTTTCGAAGAACTAATTTACTTAATATTAA
ATTGAGAAGAATTGGACATATTTGAAAAAAAATATTATTATTTTTTAAAAATAGATAAAT
AAAAATAAGGTAAACAACATTTAATGTCATTAAATTATTACTAAGTCTATATTTTGATCAT
TCAATTTTAAAAAGTTATAAAATAATCACTGAATTATTTAAAAATTTTATTTAAGTCGTTA
AATTATTCGAAAGTTTTTATTTGATCTCCAAGTAATATCTTAAATAAAAACTTTTAAATAG
TGTAATAACTTAAATAAAAAATTTTAAAATGTTTAGTGATTATTTTATAATTTTTAAAATT
AAGTAATAAAAAATAAATTTATTAATAATGTAGTGAGTAGTTTAAAAATATACGGTAACA
ACTTTTATAATTATTTGTAGTTCTAGAATCAAATCTTACGAGTCAAGTCATTAATAAACA
CTGAAATTTGCTAGCAGCAGTGCATTGTGGAGGGCAGGAAACTATAGTAAATTAAAAA
AAAAATGTATTGGGACTGGAAATTTAGCAATTTGGTTTCTCTGCTATTTCTGGGATTTGC
GTTGTGTCTGTATTTTCAATTTCACACATACCCATCACCATCTTCTCCGCTTCATATTGAT
AACA

ARE: TGGTTT (+) Anaerobic induction
CGTCA-motif: CGTCA (－) MeJA-responsiveness
GARE-motif: TCTGTTG (+) Gibberellin-responsive element
MBS: TAACTG (－) Drought-inducibility
TCA-element: GAGAAGAATA (+) Salicylic acid responsiveness
TGA-element: AACGAC (－) Auxin-responsive element
TGACG-motif: TGACG (+) MeJA-responsiveness

>CotAD_39880 GhGPX6
ATTTCAATATTTTGTTGGTGCCTTCTCTGGTGGAACATAATATATTGTGATATAGGTTCAG
GATTGTCCTCCTTATATTACCTTTTTCCTTAAAGACACTCATGTGCTACCACTCATCACCA
TAATCATCTCAACAACAACCTTAGCCTTTTTTCCACTACATAGATTGTTTCTTGGATCTC
CCCGTTGTGTACTTTTTAGGACCATATCCTTTATAAGCATCATAAAATGGGGTCGCACTA
GTGCAAGTTTTAGTTTTATAGACTTGTCTGCCTACATTTCAGCAGAAAAGATTGTCATGT
TTCATCTGTTTTAAAACAGAGCTCAAGACTAGTCCTGAGATTTGCCTTTCTGCTCAAAC
CCAACATATATTTTATTACTTGCAGTGCATCCCGTGACCTACACCACTCCGGTATATGTTA
TTCAGTGACAAAAGTTTTTGTTTCTATAAATTACCACTTCAAGATTGATTGCTAATCTCA
GAATCTTTTGAAAGACATTGAAATTTGATAAAAGTTTATTGGATGTAGGTTTTGGATGA
GAAGGATCGCACTAGGTTATTGGGTCCGCACAAGAAAGTGCAGCAATGGATTGAGAAC
ACAAGAAATGCCACAAATCCTCACTTTGATGAAGTACATAGAGTGATCATGCTGGCCA
AAGAAAGACAACAACATCAGCGATTAAAGGCAGCAAAAAATGAAGGCGGATCCAACA
TGAAAAAGCCGTTGGTTTCTAGAATGTGAGGGAAAACCGTATTACCAAGTACTTATTTC
CAGATGATAAACAATTATTAGCTTGTATAAACACGATACATGTTTTACCTATGGTTTATAC
TGCAAGTCCTATGGTTAGATTTATACTGTTGTCCTAACTCTAGTATCTATTGTTGCAAATT
TTCATTCTCAAAAGCTGTGTTGCTTGATCAGTATCACTAATGGATATTATTTGTTGCCCTT
GCTCTAAGGGTTTTGCACTTTTGCATCGTATTTTGGTCCCCCTACCCTACCACTAGCAGA
TCTCTATAGGGTACACCAAGCAGCCAAAATGCCTGCTGAAAAAACTGTACCTTAATCAT
TAAAAAGGCTACAAAAATCCCATATGGTCGCTTGTCATAAAGAAGTTTTTACTTTAAAC
CAAAAATAGAAATAACCAAATTCTAGCTATTTGACTTACACAGATATATGAACTAGCTG
GCTCCTGAAGCTCAACTGTATCAACATTCAGAGCTGTCATGTTCATGTCATTGACCTTAT
TACAAGCGAATGTTTTACAGTTATTTGATAAAGTGCTCTATAGTGAAAAACCATTAACTA
TTCTGGTCTATGGGGACTCAAATGCCCAGCTTGAAAAGTTTCAAAGACGCATTTTCCTT
AACAATGTGAATGGAATCGAATGAATTCTAGGACTGGGTTTTATGTTATCTTTTCGACCA
TTTATCTAGTTCTACACGTATTTGAGCATATATCACTTTCCATTTATATCATAAATGTCACA
TTTAAGAATCTAAACTGCTTAACAAGATCGAATAGTGTTTTCATCCACACATAGCTTCCT
TGTATCGAAAATCCCTTCTGGAGAACCACAGAAAGTTGCTAAATTGGCTGCCTCCATTA
CGTATTCTCTGCTGCAAGAGATCCTGATCCTTACTCACTTCTCCGTATTCAACGACTCGA
CAGATGGCCCTGATTTATCATACGTGTTAAGAGATGAAAAGTCCACGTGTAGACACCAT
CTTCACAATCCTACTAACCCCCACCACCTGGCCAAGATTTTTCACAAGGCTCCAAAAG
ATTCCTGTTTTACTTGAAATATGCTCGTTCGACGAAATCTAAGTGCACTTGCTGTGTCTG
CTTCTCTACTTTTAGGAAAGCGTTCCTCACCGAGTTTCAACCAAACCCTTTTGAGTTTT
CCACAGATTTCACCAGTTTATCTGGTATCTCACTCGATCAAAACAGGGTCTCCAAGGTC
TCTCGTGGGTAGTTTGAGATTTGATCATACTA

ABRE: TACGTG/CACGTG (+, －) Abscisic acid responsiveness
ARE: TGGTTT (+, －) Anaerobic induction
GARE-motif: AAACAGA (+, －) Gibberellin-responsive element
MBS: CAACTG/TAACTG (+, －) Drought-inducibility
P-box: GCCTTTTGAGT/CCTTTTG (+) Gibberellin-responsive element
TGA-element: AACGAC (+) Auxin-responsive element

>CotAD_39878 GhGPX7
TTTTACCACTTAATATTCTTCTATAATTGATTAGATTTGCGCTACCAGTAGAGTTGCTTCT
AGGTTAAAAATTACTCAAAAAGTGTGACCAGATTATTTGCTGTGTCGACCTGAGATCCC
AAGTTTTTTTCTAAATCACAGACTCCGGGCAACTGTACCTGGAGTTAGTTTAGATTCAA
GTCCTTCGGAAAAGTATCCTTACCTGCTTTGTTGTACAGACTTTGAAAGATTGTCAAGA
TGCTCTATTGCAACTACAAGAAGAAATGCCCAACGATGGCAAACCAGCACGGATTACT
CCGGACGCCAAGGAATGCATTAGCAAGCTCCGCAATGAGTTCCAGGTGAAAATGTCAG
ATGACTTGAGCACTTCACTTATACTGACTGGAGCCTTTCTAGAAGCATTGAAGTTGGTA
AACAATTTGTTGACCATGCTAAAGGTATTTGTTTATCTTTCCGAATGCTAGTAACTCTCT
TTTATATATATAAGTCTATGCCAAGGAAGATAAAAATATATTTTCTCTTGTAATTTGACAG
AAGAAGCAGCAAAAGCAACAAAGATTATTGGTAATTCAATCCCTTAAAGAGATTCAGA
ACGAAGTTACAAAAGTTTTGGATGTTCTGGGATTGCAGCCACCTTGCTCTTATAACGAG
GTGACGGGATTTACTTATTAAATGATGGTACAGATTCATGCCTTCTGTAAAAGTTAACAC
TAAAGATGAATTTCTTCTAAACAGGTTTTGCTGCAATTAAAGGAGAAAGCATTAACGA
GAGCAGGGTTGGTGGAAGATGATGTGATTCGTCTAATTAAAGAGCGAGCTGAGGTGAG
GAGAAACAAAGACATCTTGAAAAGTGATCAGATGAGAGCTCATTTGCAAGCAAAGGG
CATTGCACTCATGGATGTAGGCACGGAAACAATTTGGAGACCTTGTGTTCCTGTTCAAC
AAGACTCGGAAATAGTGCCATCAGAGGGCCAGAAGGTCCCTCCCAAGCCTGAAAGTG
CATAAGAATGCAGATCTTGATTCAAGTTCACCAAAAAAAAACATATTGAATTTGCTTCGT
ATGGTGGTTTTTATTGTCAAAACAATTTGGTGGGATCAAGGAAATTTCTAGACGGTTTT
GATTCATAATTTATAAAATGTCTTCCCGTTTCTGACCTAGTAAACTTGTGTAATGAACCA
AGTTCTTTCACGATTGGATTGATTGCCTTGCTTCTATTTGGCCTTCAGCTGCACAGAGTG
AGCCAGATTTTTTCTTTTCTGAATACGGTACTTTAGTAGTGTTGTATACTGGTAAATAAC
AGGCATGAATATGGTGGTTATATTGTCCTTTTTAAAGTTTGGTTCATATTAGGCATGATTA
TGGTATTCTCAAAATTTGAGAGGGTTTGGGCAAAAGATTAAGCCTTTTAGGATCGGGCT
TGTTTGTTTAAGGTTCGAGCTTGGTTTGGTGTGATCCGTTTTAAGTTTTAATATTGTATAT
TATGTTATTTTATATATAATGTAATTTAGAACACATTAAGAAAAATAAATTTATATTAAATA
TAGAAAATTATTTTAATGTAACCGTTAAAAAAATATTAATATGACTATATAAGTATTTTTA
ATAAATAAAAAATATATAAAATTATTAAATATTAAAATAATTTTAAGAAAATAAAAAATAA
TATGGATGGATCTAAAAGGAAATTGAGTTAGTTTTTGTAAATACGAGAGGTTCGAACAA
AAATTTATTCTCAAATTTTTAGGCGGACTAAATCTGAAAAAATATGAAATATGTTACTAT
CGGATTGAAGCCCGTCTTAGCTCGACCCACCTTTTACGTGCAACTTCTTAAAGACTGAA
TTGGGCTCAGAGGGTGAAAAACTTGTACACGTGTAAGCAGATTAAAGCATCAGATGTG
GAGACAGCATGATTCATGCCATGGAGATGGAATAGGGTAATCTAAAAGCCTGAATGGTT
TTCCAGTCTGGGACAAACAAGACAACGATTAGTATTACTCAA

ABRE: TACGTG/ CACGTG (+, －) Abscisic acid responsiveness
ARE: TGGTTT (+, －) Anaerobic induction
CGTCA-motif: CGTCA (－) MeJA-responsiveness
MBS: CAACTG (+) Drought-inducibility
TCA-element: CAGAAAAGGA (－) Salicylic acid responsiveness
TGACG-motif: TGACG (+) MeJA-responsiveness

>CotAD_59095 GhGPX8

TCTTTCTTATGTAAATTAGTTATGGAACGTCCTATAACTAACCCTTACCGATCGAACAAC
CTACGAAACGTTCGTGATTTAGAACTCCGGCAGCTTTGCGTTCTAGAAGAGCCTAGCT
CGAACCAATGCCCTCAAACGTTCAGGACATTTAAATCCGATTACTACTTCCCTTGACCG
AACCAAATAGCAATCCCCACTTGGCATGCCAATGTGTTCACGAAAAATCAATTAGACAA
TCGATCCTTTCGGAATTCCAACCGTACGTCTAGCCACACTAACTCAAACGACGTCCTTT
TTGACTTAGTATTGACTTGACTTTGTGAGTTGACAAAGTCATACTCTTAATCCAGAGAA
ACAATAAATATCGAAAATTGGGAGTTAAACAGCTCGGGTTCGTAACTCACGGGTTTTTG
ACAGGGTTAACACCGGCTTAAAGCTGAAAGGGGTTTAGTGTGGCATGAATTTAATCAT
GCTTGAAGGTTGTGAATTGTGTTTTGGCTTTGTGTGAAGGGTGATGGAATTTTGGAAA
GGAAAGGGGACTTGGAGAGCAATTGACCCAATTGTGGAAAAGAAACAAAAATTGGAT
ACAAAGCTACAAAATGCAACGTCAAATTGAAGAGAAAAGAAATAATATTCCAACTCCA
AATTGAAAAAAGAAATACAAGAGAGAGAGTAATTCTATTCCAAGTAGAAGAAGGAATA
CAAATTAAAGGATGATTTTTCTAGAAAACTAAAAGCCCCTATTTATATACATAGGGTTAT
TAAAAATAGCCTATTCTAAATTAATAAAATAATAATAATAATAATAATAATAAATAAAACT
AAATAAAATAAAATAATATCTTCTATTTTTAGATTTTTACAAAATCAAAATTGATGTTAAA
TCCTTGGCCTTCCCATCTTTATGATTTGGCCCCAAGTCAACGATTTGTCTTTCAATTTGG
TCCCTTTTTGCTCGTTTTTTACCAATTGCATCCCTGACAAGATTAAATCATAAAAAACAC
CAATTAAGCAGGGATTAATTCAGAAATAAACCAAATTAAACATAAGAATTATGTAAATT
AGTGTGTTTATCAGTTATGCCATATAAGAACTGTTTCCTTTTCTGTAATCTTATATTATTAT
AATATATATATAGAAAATATTTGTTAATATGTCGATAGAGTTTTTTGACTTCAAATGCAGG
ATTAGGGAGCTGATAAGTGTCTTATAAGGTTTATTAGATATATTATATATGCTTTAAGTCTT
TGTATTCCTTACATATTTGAAATTTAGTCCCCTTACTTTTATTATAAGGAATTTAATCCCTC
TACTTTCTAGATTTAGAAAAGCAAGTTGAATTGTTAACACATTTCTTTTGTTAAATTTAA
TATCGTTATATTGTCATTTTTTTAGTTACATGACTACCAAGTGAGTTTTTTTTTTAATTTCA
AAATATCATATCAATAAATTTAACAAAAAAAAATTAAGATGTTAATAATTGGATTTTAATT
TTGAAATCTGAAAAATAGAATAATTAAATTCTAAAAAATAAAAGTATAAGGATTAAATTC
TGAATTTATAAAACTACTGAGACTTATAGTATATTATAACAAATTTTAAACCCAAGAAAT
GCAACTGTTTAACCAATTGAGAAATGGGCAAAGCGCAAAATGTTCCGTTCACACGCCC
AAAACTCCAAAACAAGGAAAAGAGGCGCCCCATGACGTAGTCCCTGATGCAAATACG
GCCCCAATTTATCTATCCGATGTGGGAACATATGAAAATTCCACGTTGTAAATTTTGCCT
TCACGGTCATAAAAACGCGATACCCACACGACGTTTTGACCGTCGGCTCAAAATCCTAT
AAATTAACGCTTCATGGTTCCATTTTCAACTTGTATTAGCCTCGTTTAAAAAATAAATAA
ATTAAATCTTGATATAGTTGCTCTGCTTTTGCTTCTCTTATACTGAGTTCCAAGCAAAAC
CCTTTTTTTGTTTTTCGCCTGATTCCGCTGTTA

ARE: TGGTTT (－) Anaerobic induction

CGTCA-motif: CGTCA (+, －) MeJA-responsiveness

ERE: ATTTCAAA (+, －) Ethylene-responsive element

LTR: TTTCGG (－) Low-temperature responsiveness

MBS: TAACTG/ CAACTG/CGGTCA (+, －) Drought-inducibility

TCA-element: CCATCTTTTT/CAGAAAAGGA (+, －) Salicylic acid responsiveness

TGA-element: AACGAC (+) Auxin-responsive element

TGACG-motif: TGACG (+, －) MeJA-responsiveness

>Gh_D12G2260　　GhGPX9
TTGAATCCACAAGCAGCTGCTGAAGCTGAGATAATTCTGTCCTCATCTCTTTCGAAGCT
AGAGTCCTTTTGGCTCAAGGGTAATGGACGTTTTTTGCTAGGCGGGAACCAACCATCC
ATAGCAGATCTTAGCCTTGTTTGCGAGTTAATGCAACTTGAGGTACCTTTTTGTGACTT
GATTTTTTGTCCAAATTTCAATATTTTGTTGGTGCCTTCTCTGGTGGAACATAATATATTG
TGATATAGGTTCAGGATTGTCCTCCTTATATTACCTTTTTCCTTAAAGACACTCATGTGCT
ACCACTCATCACCATAATCATCTCAACAACAACCTTAGCCTTTTTTCCACTACATAGATT
GTTTCTTGGATCTCCCCGTTGTGTACTTTTTAGGACCATATCCTTTATAAGCATCATAAAA
TGGGGTCGCACTAGTGCAAGTTTTAGTTTTATAGACTTGTCTGCCTACATTTCAGCAGA
AAAGATTGTCATGTTTCATCTGTTTTAAAACAGAGCTCAAGACTAGTCCTGAGATTTGC
CTTTCTGCTCAAACCCAACATATATTTTATTACTTGCAGTGCATCCCGTGACCTACACCA
CTCCGGTATATGTTATTCAGTGACAAAAGTTTTTGTTTCTATAAATTACCACTTCAAGAT
TGATTGCTAATCTCAGAATCTTTTGAAAGACATTGAAATTTGATAAAAGTTTATTGGATG
TAGGTTTTGGATGAGAAGGATCGCACTAGGTTATTGGGTCCGCACAAGAAAGTGCAGC
AATGGATTGAGAACACAAGAAATGCCACAAATCCTCACTTTGATGAAGTACATAGAGT
GATCATGCTGGCCAAAGAAAGACAACAACATCAGCGATTAAAGGCAGCAAAAAATGA
AGGCGGATCCAACATGAAAAAGCCGTTGGTTTCTAGAATGTGAGGGAAAACCGTATTA
CCAAGTACTTATTTCCAGATGATAAGCAATTATTAGCTTGTATAAACACGATACATGTTTT
ACCTATGGTTTATACTGCAAGTCCTATGGTTAGATTTATACTGTTGTCCTAACTCTAGTAT
CTATTGTTGCAAATTTTCATTCTCAAAAGCTGTGTTGCTTGATCAGTATCACTAATGGAT
ATTATTTGTTGCCCTTGCTCTAAGGGTTTTGCACTTTTGCATCGTATTTTGGTCCCCCTAC
CCTACCACTAGCAGATCTCTATAGGGTACACCAAGCAGCCAAAATGCCTGCTGAAAAA
ACTGTACCTTAATCATTAAAAAGGCTACAAAAATCCCATATGGTCGCTTGTCATAAAGA
AGTTTTTACTTTAAACCAAAAATAGAAATAACCAAATTCTAGCTATTTGACTTACACAGA
TATATGAACTAGCTGGCTCCTGAAGCTCAACTGTATCAACATTCAGAGCTGTCATGTTC
ATGTCATTGACCTTATTACAAGCGAATGTTTTACAGTTATTTGATAAAGTGCTCTATAGT
GAAAAACCATTAACTATTCTGGTCTATGGGGACTCAAATGCCCAGCTTGAAAAGTTTCA
AAGACGCATTTTCCTTAACAATGTGAATGGAATCGAATGAATTCTAGGACTGGGTTTTA
TGTTATCTTTTCGACCATTTATCTAGTTCTACACGTATTTGAGCATATATCACTTTCCATTT
ATATCATAAATGTCACATTTAAGAATCTAAACTGCTTAACAAGATCGAATAGTGTTTTCA
TCCACACATAGCTTCCTTGTATCGAAAATCCCTTCTGGAGAACCACAGAAAGTTGCTAA
ATTGGCTGCCTCCATTACGTATTCTCTGCTGCAAGAGATCCTGATCCTTACTCACCTCTC
CGTATTCAACGACTCGACAGATGGCCCTGATTTATCATACGTGTTAAGAGATGAAAAGT
CCACGTGTAGACACCATCTTCACAATCCTACTAACCCCCACCACCTGGCCAAGATTTTT
CACAAGGCTCCAAAAGATTCCTGTTTTACTTGAAAT
ABRE: TACGTG/ CACGTG (+, －) Abscisic acid responsiveness
ARE: TGGTTT (+, －) Anaerobic induction
GARE-motif: AAACAGA (+, －) Gibberellin-responsive element
MBS: CAACTG/ CAGTTA (+, －) Drought-inducibility
P-box: CCTTTTG (+) Gibberellin-responsive element
TGA-element: AACGAC (+) Auxin-responsive element

>Gh_A12G2084 GhGPX10

AATCCACAAGTAGCTGCTGAAGCTGAGATAATTCTGTCCTCATCTCTTTCAAAGCTAGA
GTCCTTTTGGCTCAAGGGTAATGGGCGTTTTTGCTAGGCAGGAACCAACCATCCATAG
CAGATCTTAGCCTTGTTTGCGAGTTAATGCAACTTGAGGTACCTTTTTGTGACTTGATTT
TTTGTCCAAATTTCAATCTTTTGTTGTTCCCTTCTCTGGTGGAACATAATGTATTGTGATA
TAGGTTCAGGATTGTCCTCCTTAGGTTACCTTTTTCCTTCAAGACACTCATGTGCTACCA
CTCATCACCATAATCATCTCCACAACAACCTGAGCCTTTTTCCCACTACATAGATCGTTT
CTTGGATCTCCCCGTTGTGTACTTTTTAGGACCGCATCCTTTATAAGCATCATAAAATGG
GGTCGCACTAGCGCAAGTTTTAGTTATGGACTTGTCTGCCTACATTTCAGCAAAAAAGA
TTGTCATGTTTCATCTGTTTTAAAACAGAGCTCAAGACTAGTCCTGAGATTTACCTTTCT
GCTAAAACCTACATATATTTTATTGCTTGCAGTGCTTCCCCCTGACCTACACCACTCCGG
TATATGTTATTCGGTGACAAAAGTTTTTGTTTCTATAAATTACCTCTTCAAGATCGATTGC
TAATCTCAGAATCTTTGGAAAGATATTGAAATTTGATAAAAGTTTATTGGATGTAGGTTT
TGGATGAGAAGGATCGCACTAGGTTATTGGGTCCGCACAAGAAAGTGCAGCAATGGAT
TGAGAACACAAGAAATGCCACAAGTCCTCACTTTGATGAAGTACATAGAGTCATCATG
CTGGCCAAAGAAAGACAACAACATCAGCGGTTAAAGGCAGCAAAAAATGAAGGCGG
ATCCAACATGAAAAGGCCGTTGGTTTCTAGAATGTGAGGGAAAACCGTATTACCAAGT
ACTTATTTCCAGATGATAAGCAATTATTAGCTTGTATAAACACGATACATGTTTTACCTAT
GGTTTATACTGCAAGTCCTATGGTTAGATTTATACTGTTGTCCTAACTCTAGTATCTATTG
TTGCAAATTTTCATTCTCAAAAGCTGTGTTGCTTGATCAGTATCACTAATGGATATTATTT
GTTGCCCTTGCTCTAAGGGTTTTGCACTTTTGCATCGTATTTTGGTCCCCCTACCCTACC
ACTAGCAGATCTCTATAGGGTACACCAAGCAGCCAAAATGCCTGCTGAAAAAACTGTA
CCTTAATCATTAAAAAGGCTACAAAAATCCCATATGGTCGCTTGTCATAAAGAAGTTTTT
ACTTTAAACAAAAACTAGAAATAACCAAATTCTAGCTATTTGACTTATACAGATATATGA
ACTAGCTGGCTCCTGAAGCTCAACTGTATCAACATTCAGAGCTGTCGTGTTCATGTCAT
TGACCTTATTACAAGCGAATGTTTTACAGTTATTTGATAAAATGCTCTATAGTGAAAAAC
CATTAACTATTCTGGTCTATGGGGGCTCAAATGCCCAGCTTGAAAAGTTTCAAAGACGC
ATTTTCCTTAACAATGTAATCAGTGAATGGAATCGAATGAATTCTAGGACTGGGTTTTAT
GTCATCTTTTCGACTATTTATCTAGTTCTACACGTATTTGAGCATATAGCACTTTCCATTTA
TATCATAAATGTCACATTTAAGAATCTAAATTGCTTAACAAGATCGAATAGTGTTTTCAT
CCACACATAGCTTCCTTGTATCTAAAATCGCTTCTGGAGAACCACAGAAAGTTGCTAAA
TTGGCTGCCTCCATTACGTATTCTCTGCTGCAAGAGATCCTGATCCTTACTCACCTCTCC
GTATTCAACGACTCGACAGATGGCCCTGATTTATCATACGTGTTAAGAGATGAAAAGTC
CACGTAGACACCATCTTCACAATCCTACTAACCCCCACCCCCGGGCCAAGATTTTTCAC
AAGGCTCCAAAAGATTCCTGTTTTACTTGAAAT

ABRE: TACGTG (+, －) Abscisic acid responsiveness

ARE: TGGTTT (+, －) Anaerobic induction

GARE-motif: AAACAGA (+, －) Gibberellin-responsive element

MBS: TAACTG/CAACTG (+, －) Drought-inducibility

P-box: CCTTTTG (+) Gibberellin-responsive element

TCA- element: CCATCTTTTT (－) Salicylic acid responsiveness

TGA-element: AACGAC (+) Auxin-responsive element

>CotAD_39520 GhGPX11

TTACCACTTAATATTCTTCTATAATTGATTAGATTTGCGCTACCAGTAGAGTTTCTTCCAG
GTTAAAAGTTACTCAAAAAGTCTGACCAGATTATTTGTTGAGTCAACCTGAGATCCCAA
GTTTTTTTTTCTGAATTACAGACTCCAGGCAAATGTACCTGGAGTTAGTTTAGATTCAA
GTCCTTCGGAAAAGTATCCTTACCTGCTTTGTTGTACAGACTTTGAAAGATTGTCAAGA
TGCTCTATTGCAACTACAAGAAGAAATGCCCAACGATGGCAAACCAGCTCGGATTACT
CCGGACGCCAAGGAATGCATTAGCAAGCTCCGCAATGAGTTCCAGGTGAAAATGTCAG
ATGACTTGAGCACTTCACTTATACTGACTGGAGCCTTTCTAGAAGCATTGAAGTTGGTA
AACAATTTGTTGACCATGCTAAAGGTATCTGTTTATCTTTCCGAATGCTAGTAACTCTCT
TTTATATATATATAAGTCTATGCCAAGGAAGATAAAAATATATTTTCTCTTGTAATTTGAC
AGAAGAAGCAGCAAAAGCAACAAAGATTATTGGTAATTAAATCCCTTAAAGAGATTGA
GAACGAAGTTACAAAAGTTTTGGATGTTCTTGGATTGCAGCCACCTTGGTCTTATAACG
AGGTGATTGGATTTATTTATTAAATAATGTTACAGATTCATGCCTTCTGTAAAAGTTAAC
ACTTAAGATGATGAATTTCTACTAAACAGGTTTTGCTGCAATTAAAGGAGAAAGCATTG
ACGAGAGCCGGGTTGGTGGAGGATGATGTGATTCGTCTAATTAACGAGCGAGCTGAAG
TGAGGAGAAACAAAGACTTCTTGAAAAGTGATCAGATGAGAGCTCATTTGCAAGCAA
AGGGCATTGCACTCATGGATGTAGGCACGGAAACAATTTGGAGACCTTGTGTTCCTGT
TCAACAAGACTCGGAAATAGTGCCATCAGAGGGCCAGAAGGTCCCTCCCAAGCCTGA
AAGTGCATAAGAATGCAGATCTTGATTCAAGTTCACCAAAAAAAAAAAACATATTGAAT
TTGCTTCGTATGGTGGTTTTTATTGTCAAAACAATTTGGTGGGATCAAGGAAATTTCTAG
ACGGTTTTGATTCATAATTTATAAAATGTCTTTCCCTTTCTGACCTAGTAAACTTGTGTAA
TGAACCAAGTTCTCTAACGATTGGATTGATTGCCTTGCTTCTATTTGGCCTTCAGCTGCA
CAGACAGCCAGCCAGATTTTTTCTCTTCTGATTACAGTACTTTAATAACAGGCATGAATA
TGGTGGTTATATTGTCCTTTTTAAAGTTTGGTTCAGATTAGCAATGATTATGGGTCACGC
TACCCACTTATGCCCAACCCAAAGGTCTCCCAAATTATTCGGGTAAAAGATTAGGCCCT
TCAGACTTGGGTTTAAGCATTTAAGGTTTGAGCTGAGTCTCGTTTGATCTGTTTTAAGTT
ATCATATTATATATTATGTTATTTTTATATAATTTAGAGTAAATGAAAAAAATAAACTTATA
CTAAATATAGAATATTATTTTAATGTAGCAGTTAAAAAAAATATCAAGATGAATATATAAT
TATTTTTAATAAATAAAAAATATATAAAATTATTAAAAATTAAAATAATATAATATAATTTT
TAAAAAAAATAGAAAATAATATGAACGGATTTAAAATGAAATTTAATTAATTTTTGTAAA
TATAAGAGGGTTCTAAAGCAGATTAAACTGAGAAAGTATGAAATATGTTACTATTGTATT
TAAGGCCGTCTTAATTCGACCCATGAACACGAGTTTATGATAAGCTGTTAATGTCGGTCT
ACGATCAGAGGGTGAAAAACTTGTACACGTGTAAGCAGATTAAAGTATCAGATGTGGA
GACAGCATGGTTCATGCCATGGAGATGGAATAGGGGAATCTAAAAGCCTGAATGGTTT
TCCAGTCTGGGACAAACAAGACAACGATTAGTATTACTCAA

ABRE: CACGTG (－) Abscisic acid responsiveness

ARE: TGGTTT (+, －) Anaerobic induction

CGTCA-motif: CGTCA (－) MeJA-responsiveness

GARE-motif: AAACAGA (－) Gibberellin-responsive element

MBS: TAACTG (－) Drought-inducibility

TCA-element: CAGAAAAGGA/TCAGAAGAGG/GAGAAGAATA (－) Salicylic acid responsiveness

TGACG-motif: TGACG (+) MeJA-responsiveness

>CotAD_51884 GhGPX12

ACATATCTTAGCTTTTTCTTCTATAGAGAATCTTTTTCTAGAAAACTCTAGAAAAACGAA
ATAATTAGTTTGACAAACTTTTTAAGGTTTAAGTCTGCTCTTAGTCCTTATACTCTTTGC
AGATTTAAAATTTGGTCCCTCTATCTTGAATTCCAAGATCTCTTTGGCTTCTGATTTAATC
AAAATTCAAGTGCAATCGATGATAAGAGGATAAACATACCTCATCGCACTCGAACCCAC
ATTCTCCTGCACTGGCAACAATCCACAACTCCAAATTCTTAATTGAAAACCTTATACCTT
TGATTAAACATTTTTATTTGTTTATTTGACCTTTCAATCATGTAATATTTAATCTTGCGAAC
ATTTTTGTTTAAATGTATCATAATTTAATTAAACAAATTTATTTAATTGAATTTTAATTATT
AAAATAAAAAGTCTAAATTCATAAATTAAAAACAAAAAACTAAATTTTGTAGGTTAAAA
TTAGTTTGGATTCTCGCAACGGTGCACAAATTATTTGTGTGTATTAATATTATAAATTTTT
TTGTTTGAATTAATTAACGATTTGAAAAAATATAAATTTAATTTAATCAATGTTATTAAAA
TGTGTTTTCATTTAAAAAAATATTTTAAGTTAAAATTTTACCATATATTTGTGTTAATTTGT
TTTATAATTTGTATTTCCTATAATTTATTTAATGTAATTATAAATAGAATTATAATTTTGTATT
TTTAGAGTATATATCCACACTCATTTCAATTTATGCACATTTAGAGAGTGTACTATTTAAG
TTAAATGTCACAAAAATATTGATGTAAAAATTAAGTAACACTTCTTGTAATCATATAAAA
ATATGACATGTAATAATATATTGAAAATAAAATAAGCTTAAATGCATTGATATTGTGAAAT
TTGGCGAGATATCAACAAAAACATAAGGAATTTTTTATCCAAATTAGTTTCTATATTTTAA
TGCGGAATTTGTTTTGAATTAATTTTTTTAAATCGTTAACAGTTAGACTTGGTGACAAAT
GACAATTTAGAAGTTTCCTTTTCTCTAACCTCATATTATTATAATATATATATATGAAAAAT
ATTTGTTACTATGTCCGTAAAGTTTTTAGACTTTAAATGCAGAATTAGGGAGTTTATAAG
TGTCTTATAAGGTTTATTAGATATATTAGATATGCTCTAAGTCTTTGTATTCCTTGCATATT
TTAAATTTAGTCCCCTTACTTTTATTATAAATAACTTAATCCTTCTACTTTTTAGATTTAAA
TAAGCAAGTTTAATTGTTAACACCGTTAAATTTCTTTTGTTAAATTTAATATAATTTATATT
GTCATTTTTTTAGTTACATGACTATCAAGTGTGTTTTTTTTTTAATTTCAAAATATCATATC
AATAAATTTGACATAAAAAATTAAGATGTTAACAATTGAATTTTAATTTTGAAATCTGAA
AAATAGAATAATTAAATTCCAAAAAATAAAATATAAGGATTAAATTATAAATTTATGAAA
CTACTGAGACTTATAGTATATTTTAACAAAATTTAAACCCAAGGAATGCAACAGTTTAA
CCAATTGGGAAATGGGTAAAGCGCAAAATGTTCCGTTCACACGCCCAAAACTCCAAAA
CAAGGAAAAGAGGCGCCCCATGACGTAGTCCCTGATGCAAATACGGCCCCAATTTATC
TATCCGATGTGGGGATATATGAAAATTCCACGTTGTAAAATTTGCCTCACGGTCATAAAA
ACGCGATACCCACGCGACGTTTTGACCGTCGGCTCAAAATCCTATAAATTACGCTTCAT
GGTTCCCTTTTCAACTTGTATTAATTAGACTCGTTTGAAAAATAAATTAATAAAATCTTG
ATATAGTTGCTCTGCTTTTGCTTCTCTTATACTGAGTTCCAAGCAAAACCCCTTTTTGTT
TTTCGTCTGATTCAGCTAAGGTCTTCTTTGGGTAGTTTGAGATTTGAGTTATTCATCATA
CT

ERE: ATTTCAAA (+, －) Ethylene-responsive element
MBS: TAACTG/CGGTCA (+, －) Drought-inducibility
TCA-element: CAGAAAAGGA (－) Salicylic acid responsiveness
TGACG-motif: TGACG (+) MeJA-responsiveness

>CotAD_36707　　GhGPX13
GGTTTTGGTTGGGACGAGCATAGGCAGCTCGTTGTTGCTGAAGATGCAGTTTGGGAAT
CCTATGTAAAGGTAAATGTATTTCAAGTATTTATTTGTTTTTTTACAAAACTTATAACTAA
TATGATTTTCTCATTTTTTTAGAGTCACAAAGAAGCCGCTCAGTTCAGACATCGTTCTTT
CCCTTACTACAACCAGCTTACTGCCATATACGCAAGAGATCGAGTGACTGGGAAAGAC
GCTCAAATAGCTGCTGATGTTCTTGAAAAAATACATGCTGAGGATGTACCTACTACAGA
TATGAATGAAGAGAGAAACACATTCTATGACTGCGAAGCTTACGTCTCTTTAGACAACA
TGGATGTTTCTGGTACGGAGCCGCGAGGAGATAGAGACCAAGGGGGTTCCTCATCTTC
AAACAAGAGAAAGAAGAAATCTGATGCTCGTGATAATGTGTATTCTCCATTTGATGAGG
TTGCCACTTTGTTGGCCGAAAACATCAAGGCCGTTGGCGATCAAATCAGTAGGAGTAT
TGCCTCCGAGGTGGTAGTTCAGCAGAAGTCAGAAGAATATCAAAAGATGCGAAGAGAA
AGCTTCAAATTTATATTCATCCTTATGGGAAATTGAAGGTTTATCCGACGATCAGCGGTA
TGAAGCTTTGAGTAAAATTCCAGATCATCCAACTCAAATGATCGTTTTCTTTAGTTTACC
TTCTGTTGCGCGATTAGAATGGGTCAGAAGATTTCTTTCTCACCATTAAAAATCAATGTT
CAAACTTTTTGATGTTGTAAAACTATATAACATATGGATTATGATGTAGAATTTCATTTTC
ATCTAACCTTTTCTTAATATAAGTTAATTATGTTTATGCAGAATATAATTCTCAAGTTATAT
TTTGATTTTAGTAAATTCATATAAGAACATTTTATTATTAAGAATTTTATGAAATTATATAT
CATTATTAAATTATATTTAATATTAATTATTTTAAATTGTATAAATTCATATAAGAAAATTTA
TTATCAAGAATTTTATGAAATTATATATTATTATTAAATTATATGTAATAATAATTATTTTAA
ATTATACAAATTCATATAAGATAATTTATTAGTTATAATTATTTTAAATTTTATTAAATCATA
TATTTTAATTAAAATATATTTAATATTAATTATTTCAAATTTTCTTTTATAGTATCATATATTA
TGATTTGAGTAAATTCATATAAGAATATTAATTATTAAGAATTTTATTAAATTATATATTTTA
ATTATAATATATTTAATAATAATTATGTTTAAATATAATTAAATTATTTATTATTGATAATAAT
AATCTTATTAAAATTTAAATAACAATAACAATAATCATTTACCAAAACAAATTTATGCTA
AGGGTATTCTAGTCATTTTAGTTTTTTCCATTATGCTATTACACATCTATTTCATTCAACCA
AACACAAGATTACTATTACGCTTTTATTCCATTACATTCAACCAAATAATTGATTTGTTAT
TACACCTTTATTACATTACGCCTCTAATCCAATACAGCGAACCAAACGTGTCCTAAGTTT
TAAATTAGGTTTGCTAATTCATTACGGTGGACCAAACTTTCGTGCAGAAGAAGTCAAA
AAAAGAGGAAGAAGATAACTTCGGTGCTCCAAATTTGGTGAAATAAACGATAATTTCA
TTAACTAATTTAATAATAAGGTGTCATTTTTCATTACATAATCTTTTCATTTTTTTAATTTT
AAACCCAAAAAAGAAATCAATTTTTAAAAAAAATATTGCATAAATCTTCTTTACATGGT
AAGCTAATAATTTTCCCAGTCTGGCAAACCCAGCTAATAACAATAACCATAAAAACCCA
ATCTCTTGATTCTCTTCCCATATATATATATATTGCAGCCAAAAAAACAGCTACAAAAAAAC
CCAAATCACAGAGAAAAGTTCACAAAGAACCCAAAGTTGTTAACTTTTTTGATAACCC
AAAACACAAAA

ERE: ATTTCAAA (+) Ethylene-responsive element

GARE-motif: TCTGTTG (+) Gibberellin-responsive element

LTR: CCGAAA (+) Low-temperature responsiveness

TCA-element: GAGAAGAATA/CCATCTTTTT (+, －) Salicylic acid responsiveness

Supplementary Table 1. A list of the primers used in all experiments.

Genes		Primers
**qRT-PCR**		
UBQ7	F	5' GAAGGCATTCCACCTGACCAAC 3'
	R	5' CTTGACCTTCTTCTTCTTGTGCTTG 3'
GhGPX1	F	5' CTGCTTCTGGGTTTGTCTTTAAC 3'
	R	5 'GGGTATCCTTCCCATCAATATCC 3'
GhGPX2	F	5' CCCACCTTTACTACCCCATCT 3'
	R	5' GATTTCTGAGGAACTGATTCAGAC 3'
GhGPX3	F	5' TATGCTCCAACCACATCACC 3'
	R	5' ATGGGCACACCGCTTATAC 3'
GhGPX5	F	5' TACGAGACCTACTCGGATCTTC 3'
	R	5' CTTACACTGGGAACGCAACTA 3'
GhGPX8	F	5' CTTCTCTTATACTGAGTTCCAAGCA 3'
	R	5' CCTCTTGCATCCTTAACAGTGA 3'
GhGPX13	F	5' AGACAGAGCCCGTCTACAA 3'
	R	5' TGGCCATCCTTGTCAACTAAA 3'
pYES2::GhGPX		
GhGPX1	F	5' CCCAAGCTTTGGCTTCCATGTCTTTCTCTGC 3' (*Hin*d III)
	R	5' CCCGGATCCTCATGTCGCGAGGAGCTTCT 3' (*Bam*H I)
GhGPX2	F	5' CCCAAGCTTATGGGTGCGTCTGAATCAGTTC 3' (*Hin*d III)
	R	5' CCCGGATCCTCACGTATCCACTCCCAATGC 3' (*Bam*H I)
GhGPX3	F	5' CCCAAGCTTATGTCCGCTGAAGACGCCTCC 3' (*Hin*d III)
	R	5' CCCGGATCCTAAGAAGATCCGAGTAGGT 3' (*Bam*H I)
GhGPX6	F	5' CCCAAGCTTATGTCCGCTGCTGAGTCCTCC 3' (*Hin*d III)
	R	5' CCCGGATCCTCAGCCAAGCAGTTTCTTTA 3' (*Bam*H I)
GhGPX7	F	5' CCCAAGCTT ATGTCCACGACCCTAACTCCA 3' (*Hin*d III)
	R	5' CCCGGATCCTATTCCAGTAACTTCTTTATAT 3' (*Bam*H I)
GhGPX8	F	5' CCCAAGCTTATGTCCGCTTCTCAATCTTCTA 3' (*Hin*d III)
	R	5' CCCGGATCCTCAAGCCAGCAGTTTCTTA 3' (*Bam*H I)
GhGPX13	F	5' CCCGGATCCATGGGTGCTTCTGAATCAGT 3' (*Bam*H I)
	R	5' CCCGAATTCTCACATGTCCACTCCCAAAG 3' (*Eco*R I)
pHBT::GhGPX		
GhGPX1	F	5' CCCGGTACCATGGCTTCCATGTCTTTCTC 3' (*Kpn* I)
	R	5' CCCGGATCCTGTCGCGAGGAGCTTCTGAA 3' (*Bam*H I)
GhGPX3	F	5' CCCGGTACCATGGCTGAAGACGCCTCCC 3' (*Kpn* I)
	R	5' CCCGGATCCAGAAGATCCGAGTAGGTCC 3' (*Bam*H I)
GhGPX5	F	5' CCCGGTACCATGGCTGAAGACGCCTCCC 3' (*Kpn* I)
	R	5' CCCGGATCCAGAAGATCCGAGTAGGTCC 3' (*Bam*H I)
GhGPX6	F	5' CCCGGTACCATGGCTGCTGAGTCCTCC 3' (*Kpn* I)
	R	5' CCCGGATCCGCCAAGCAGTTTCTTTAT 3' (*Bam*H I)
GhGPX7	F	5' CCCGGTACCATGACGACCCTAACTCCAA 3' (*Kpn* I)
	R	5' CCCGGATCCTTCCAGTAACTTCTTTATATC 3' (*Bam*H I)
GhGPX8	F	5' CCCGGTACCATGGCTTCTCAATCTTCTA 3' (*Kpn* I)

	R	5' CCCGGATCCAGCCAGCAGTTTCTTAA 3' (*Bam*H I)
Cloned genes		
CotAD_39521	F-1	5' GATCCCTCAAAGCAAGAAACAA 3'
	R-1	5' GCCCACCACCATGAATGTCAAATTT 3'
	F-2	5' GGACGACCTGGGTGGCACATAG 3'
	R-2	5' TTATGCACTTTCAGGCTTGGGAG 3'
GhGPX4	F-1	5' TGTCTGCAGATGGGTGCTTCTGAATCAGTT 3'
	R-1	5' GGGACTAGTCTTTTTACAAACTCGAGCAGC 3'
	F-2	5' TGCACAAGATACAAGGCTGAATATC 3'
	R-2	5' CTACTTTTTACAAACTCGAGCAGCG 3'
GhGPX9/10	F	5' ATGCTCGTTCGACGAAATCTAAGTG 3'
	R	5' TTATTCACTCAAAGCCTGAGGTGAC 3'
GhGPX11	F	5' ATGACGACCCTAGCTCCAAATAACC 3'
	R	5' CCCTTATTCCAGTAACTTCTTTATA 3'
GhGPX12	F	5' CCCGGTACCATGGCTTCTCAATCTTCTA 3'
	R	5' CCCGGATCCAGCCAGCAGTTTCTTAA 3'
